# CEGA: a method for inferring natural selection by comparative population genomic analysis across species

**DOI:** 10.1186/s13059-023-03068-8

**Published:** 2023-10-03

**Authors:** Shilei Zhao, Lianjiang Chi, Hua Chen

**Affiliations:** 1 CAS Laboratory of Genomic and Precision Medicine, Beijing Institute of Genomics, Chinese Academy of Sciences, Beijing, 100101 China; 2 China National Center for Bioinformation, Beijing, 100101 China; 3 School of Future Technology, College of Life Sciences and Sino-Danish College, University of Chinese Academy of Sciences, Beijing, 100049 China; 4 CAS Center for Excellence in Animal Evolution and Genetics, Chinese Academy of Sciences, Kunming, 650223 China

**Keywords:** HKA test, Natural selection, Allele frequency spectrum, Ancestral inference, Closely related species

## Abstract

We developed maximum likelihood method for detecting positive selection or balancing selection using multilocus or genomic polymorphism and divergence data from two species. The method is especially useful for investigating natural selection in noncoding regions. Simulations demonstrate that the method outperforms existing methods in detecting both positive and balancing selection. We apply the method to population genomic data from human and chimpanzee. The list of genes identified under selection in the noncoding regions is prominently enriched in pathways related to the brain and nervous system. Therefore, our method will serve as a useful tool for comparative population genomic analysis.

**Supplementary Information:**

The online version contains supplementary material available at 10.1186/s13059-023-03068-8.

## Background

Comparative analysis of genomic sequences from multiple species is useful for studying the origin and evolution of novel traits [1]. In recent years, with the development of sequencing technology, population genomic data of numerous species have become available. Integrative evolutionary analysis of both between-species divergence and within-species polymorphism, aka comparative population genomics, potentially has higher power and achieves more accurate inference of parameters by using more data information and is thus in increasing demand for population and comparative genomic studies. Some well-known methods include the McDonald-Kreitman (MK) test [2] and Hudson-Kreitman-Aguadé (HKA) test [3]. The MK test identifies recurrent selection on a protein-coding gene by evaluating the excess or deficiency of nonsynonymous divergent sites over synonymous divergent sites using within-population polymorphic sites as a neutral control. The HKA test also involves comparing genomic divergence between two species to polymorphism data within a species. The HKA test does not classify mutations into nonsynonymous and synonymous sites; instead, it compares the ratios of divergent sites over polymorphic sites across different loci. Therefore, the HKA test requires polymorphism and divergence data from multiple genomic regions but is applicable to noncoding genomic regions.

Both the HKA and MK tests use the chi-squared test or Fisher exact test to evaluate the fit of data to the null hypothesis of neutrality. The chi-squared test has limited power and provides no insights into the selective process without inferring parameters. Multiple methods were proposed to tackle the problem of low power. Some methods modify the HKA test and include more summary statistics [4], and other more complicated parametric methods model the data pattern by incorporating the evolutionary processes. The MKPRF approach [5] extends the MK test using the Poisson random field framework [6]. The method was developed by assuming that the entries of the MK table follow independent Poisson distributions, and the expected values of the entries are predicted theoretically with a population genetic model composed of multiple parameters, including selection intensity. MKPRF was later extended to high-dimensional MKPRF by exploiting patterns of polymorphism and divergent sites of multiple species (HDMKPRF [7]). Analogously, MLHKA [8] is a model-based method for HKA. It explicitly models the numbers of divergent sites between two species and polymorphic sites in a population of a single species and applies a maximum likelihood ratio test to detect directional selection. Gronau et al. developed a similar approach, INSIGHT [9], which models the polymorphism pattern within a single population using an empirical approach by treating the allele frequency of the mutant under selection as an unknown parameter; INSIGHT uses a hidden Markov model to identify the putatively selected genomic regions. Parametric methods explicitly model the effect of selection on the genetic polymorphism pattern, and thus, in addition to being significant tests, these methods are useful for inferring parameters of the selective sweep processes. Numerous studies have concentrated on applying these methods to analyze genomic data to characterize the essential parameters of natural selection, such as the distribution of fitness effects and the rate of adaptation [10–16].

In addition to directional selection, HKA-type methods are also useful and applied extensively to identify balancing selection. Balancing selection favors heterozygous genotypes in populations (species) and tends to increase the genetic diversity within a population (species) and shared polymorphic sites between populations (species). Other than HKA-type methods, several new methods have been developed recently to identify loci under balancing selection using genomic polymorphisms. Two composite likelihood ratio tests (${T}_{1}$ and ${T}_{2}$) were developed for detecting long-term balancing selection using the expected allele frequencies and the fixation probability of nearby mutations [17], which require extensive simulations under a known demographic history. The summary statistics ${\beta }^{\left(1\right)}$ and ${\beta }^{\left(2\right)}$ were proposed to detect balancing selection based on the clustering pattern of multiple mutations with similar frequencies around the selected alleles [18, 19]. Simulations demonstrated that $\upbeta$ statistics outperform the other existing methods, including HKA and composite likelihood ratio tests [17, 18].

In this paper, we present a new parametric approach, CEGA, for detecting natural selection in the comparative population genomic framework. CEGA takes multiple genomic sequences from two species. It has several advantages over existing approaches. First, CEGA models within-species polymorphisms and between-species divergent sites and thus can analyze both coding regions and noncoding regions, satisfying the growing need for studies on regulatory and noncoding genomic regions. Second, CEGA explicitly models the shared genetic polymorphisms among closely related species, which are ignored in existing methods, and appropriate for analyzing species data with a wide range of divergence times. Thus CEGA has higher power to detect selection than existing methods, especially for closely related species. Third, CEGA is computationally very efficient and can analyze large-sample genome-wide data within several hours, while the existing parametric methods, e.g., MLHKA, require intensive computation due to the inclusion of Markov chain Monte Carlo (MCMC) approaches. Fourth, CEGA can identify both positive selection and balancing selection and outperforms the existing methods in terms of power for detecting selection; furthermore, the method can accurately infer evolutionary parameters, including selection intensity, providing more insights into the selection process.

We applied the method to population genomic data of humans and chimpanzees and identified a set of genes under lineage-specific positive selection in humans and enriched in gene regulatory pathways, metabolism, and immune-system-related pathways. Furthermore, the method identified 342 novel genes with selection signals only in regulatory and noncoding regions, including the human accelerated regions. Multiple genes in this set are functionally critical in the brain and nervous systems. We also compiled a list of genes under balancing selection, of which a high proportion are related to the immune system, including the well-known major histocompatibility complex (MHC) loci. We expect CEGA to be a useful tool for evolutionary comparative genomic analysis.

## Results

### Model

**Fig. 1:**
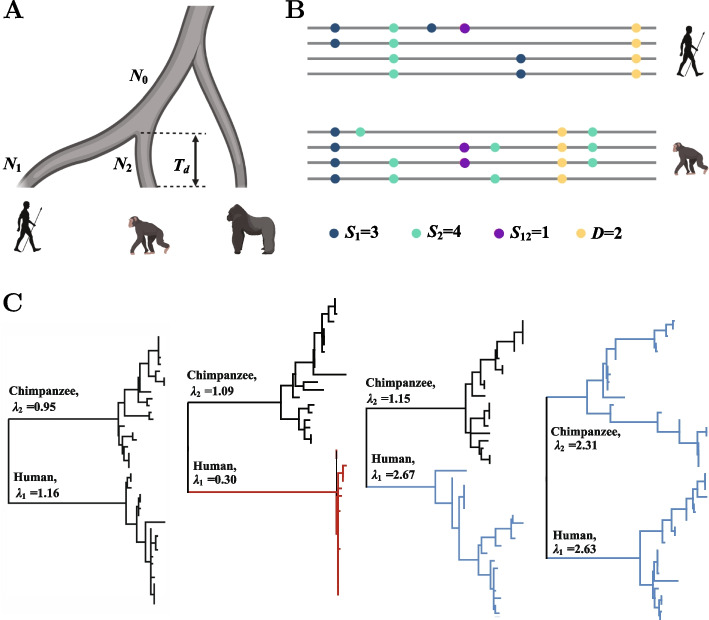
Illustration of the CEGA method. **A** Parameters of the CEGA model. Global parameters: divergence time ${T}_{d}$, effective population sizes of the two differentiated species ${N}_{1}$ and ${N}_{2}$ and of the common ancestor ${N}_{0}$. Locus-specific parameters: scaling coefficients ${\lambda }_{1}^{l}$ and ${\lambda }_{2}^{l}$ of ${N}_{1}$ and ${N}_{2}$. **B** Four locus-specific summary statistics of the observed data, ${S}_{1}$, ${S}_{2}$, ${S}_{12}$, and $D$. **C** Examples of gene genealogies of samples under positive selection (red) and balancing selection (blue). The genealogies were constructed from simulated data using MEGA (v. 11)

Suppose that ${n}_{1}$ and ${n}_{2}$ aligned genomic sequences are collected from two species (Fig. 1A). The genomes can be divided into $L$ loci or regions according to physical positions or biological functions for identifying locus-specific effects of natural selection. For each locus $l$, the between-species divergence and within-species polymorphism pattern of the two species can be summarized into four summary statistics, including the polymorphic sites within species 1 (${S}_{1}^{l}$), polymorphic sites within species 2 (${S}_{2}^{l}$), shared polymorphic sites of both species 1 and 2 (${S}_{12}^{l}$) and divergent sites that are fixed but with different alleles in species 1 and species 2 (${D}^{l}$) (Fig. 1B). In the PRF framework, the four types of sites are assumed to be independent and follow a Poisson distribution with the mean parameterized according to population genetic models [6], and correlations between the summary statistics are known to have a weak effect on inference [3, 8].

The population genetic parameters include two categories. The global demographic parameters are shared among genome-wide loci, including the divergence time of the two species ${T}_{d}$ and the effective population sizes of the ancestral species (${N}_{0}$) and of the two descendent species (${N}_{1}$ and ${N}_{2}$). The locus-specific parameters include the mutation rate ${\mu }^{l}$ and two scaling coefficients ${\lambda }_{1}^{l}$ and ${\lambda }_{2}^{l}$ of ${N}_{1}$ and ${N}_{2}$ at locus $l, 1\le l \le L$. ${\lambda }_{1}^{l}$ and ${\lambda }_{2}^{l}$ are added to model the locus-specific effect of natural selection and will be discussed in more detail in the following paragraphs. The expected values of the aforementioned four types of mutation sites are derived explicitly as a function of the parameters $\Gamma =\{{N}_{0},{N}_{1},{N}_{2},{T}_{d},{\mu }^{l},{\lambda }_{1}^{l},{\lambda }_{2}^{l},1\le l\le L\}$, as shown in Eqns. 4, 5, 6 and 7.

### Polymorphism pattern (${S}_{1}$, ${S}_{2}$, ${S}_{12}$, and $D$) under neutral evolution

The expected values of ${S}_{1}$, ${S}_{2}$, ${S}_{12}$, and $D$ are obtained from the analytical equations of the joint allele frequency spectrum (JAFS) of multiple populations derived using coalescent theory [20]. ${S}_{i,j}\left({n}_{1},{n}_{2}\right)$ denotes an entry of JAFS, representing the number of sites with $i$ copies of the derived allele in a sample of ${n}_{1}$ haplotypes from Population 1 and $j$ copies of the derived allele in a sample of ${n}_{2}$ haplotypes from Population 2. The JAFS for two species (populations) is obtained by summing two components: the “ancient” segregating sites that arose in the ancestral population and the “new” segregating sites that arose in the two descendant populations,

1 $$\begin{array}{c}\mathbb{E}\left({S}_{i,j}\left({n}_{1},{n}_{2}\right)\right)=\mathbb{E}\left({S}_{i,j}^{a}\left({n}_{1},{n}_{2}\right)\right)+\mathbbm{1}\left(j=0\right)E\left({S}_{i,0}^{n}\left({n}_{1},0\right)\right)\\ +\mathbbm{1}\left(i=0\right)E\left({S}_{0,j}^{n}\left(0,{n}_{2}\right)\right)\end{array}$$

where $\mathbbm{1}\left(\cdot \right)$ is the indicator function and ${\mathbb{E}}\left({S}_{i,j}\left({n}_{1},{n}_{2}\right)\right)$ denotes the JAFS of two species. The “ancient” segregating sites ${\mathbb{E}}\left({S}_{i,j}^{a}\left({n}_{1},{n}_{2}\right)\right)$ are

2 $$\begin{aligned}\mathbb{E}\left({S}_{i,j}^{a}\left({n}_{1},{n}_{2}\right)\right)&=\sum\limits_{{m}_{1}=1}^{{n}_{1}}\sum\limits_{{m}_{2}=1}^{{n}_{2}}{g}_{{n}_{1},{m}_{1}}\left({T}_{d}\right){g}_{{n}_{2},{m}_{2}}\left({T}_{d}\right)\\ &\quad \times \sum_{\{0\le {k}_{1}\le {m}_{1},{k}_{1}\le i\}}\sum_{\{0\le {k}_{2}\le {m}_{2},{k}_{2}\le j\}}p\left({k}_{1}\to i\left|{n}_{1},{m}_{1}\right.\right)\\ &\quad\times p\left({k}_{2}\to j\left|{n}_{2},{m}_{2}\right.\right)\\ &\quad\times p\left({k}_{1},{k}_{2}\left|{m}_{1},{m}_{2}\right.\right)\mathbb{E}\left({S}_{{k}_{1}+{k}_{2}}^{0}\left({m}_{1}+{m}_{2}\right)\right)\end{aligned}$$

where ${g}_{{n}_{1},{m}_{1}}\left({T}_{d}\right)$ is the distribution of ancestral lineages at time ${T}_{d}$ of the ${n}_{1}$ haplotypes at present; $\mathrm{p}\left({k}_{1}\to i\left|{n}_{1},{m}_{1}\right.\right)$ is the Polya-Eggenberger distribution; and ${\mathbb{E}}\left({S}_{{k}_{1}+{k}_{2}}^{0}\left({m}_{1}+{m}_{2}\right)\right)$ is the expected number of segregating sites in the common ancestral population ${N}_{0}$, which is ${\mathbb{E}}\left({S}_{{k}_{1}+{k}_{2}}^{0}\left({m}_{1}+{m}_{2}\right)\right)=2{N}_{0}\mu /\left({k}_{1}+{k}_{2}\right)$.

The newly occurring (“new”) segregating sites in the two descendant species ${\mathbb{E}}\left({S}_{i,0}^{n}\left({n}_{1},{n}_{2}\right)\right)$ are

3 $$\begin{aligned}\mathbb{E}\left({S}_{i,0}^{n}\left({n}_{1},{n}_{2}\right)\right) &=\mathbb{E}\left({S}_{i}^{n}\left({n}_{1}\right)\right)\\ &=\sum_{{m}_{1}=1}^{{n}_{1}}{g}_{{n}_{1},{m}_{1}}\left({T}_{d}\right){\mathbb{E}}\left({S}_{i}\left({n}_{1}\right)\left|{m}_{1}\right.\right)\\ &=\sum _{{m}_{1}=1}^{{n}_{1}}{g}_{{n}_{1},{m}_{1}}\left({T}_{d}\right)\frac{\left({n}_{1}-i-1\right)!\left(i-1\right)!}{\left({n}_{1}-1\right)!}\\ &\quad \times \sum\limits_{k={m}_{1}}^{{n}_{1}}k\left(k-1\right)\left(\genfrac{}{}{0pt}{}{{n}_{1}-k}{i-1}\right)\mathbb{E}\left({T}_{k}\left|{m}_{1}\right.\right)\mu ,\\ &\qquad 0 < i\le {n}_{1} \end{aligned}$$

where $\mu$ is the mutation rate and ${\mathbb{E}}\left({T}_{k}\left|{m}_{1}\right.\right)$ is the conditional coalescent time. The details of the exact form can be found in Chen (2012) [20].

The expected values of ${S}_{1}$, ${S}_{2}$, ${S}_{12}$, and $D$ can then be obtained by summing the corresponding entries of ${\mathbb{E}}\left(S\left({n}_{1},{n}_{2}\right)\right)$ directly,

4 $$\begin{array}{c}\mathbb{E}{S}_{1}=\sum\limits_{i=1}^{{n}_{1}-1}{\mathbb{E}}\left({S}_{i,0}\left({n}_{1},{n}_{2}\right)\right)+\sum\limits_{i=1}^{{n}_{1}-1}{\mathbb{E}}\left({S}_{i,{n}_{2}}\left({n}_{1},{n}_{2}\right)\right)\end{array}$$

5 $$\begin{array}{c}\mathbb{E}{S}_{2}=\sum\limits_{j=1}^{{n}_{2}-1}{\mathbb{E}}\left({S}_{0,j}\left({n}_{1},{n}_{2}\right)\right)+\sum\limits_{j=1}^{{n}_{2}-1}{\mathbb{E}}\left({S}_{{n}_{1},j}\left({n}_{1},{n}_{2}\right)\right)\end{array}$$

6 $$\begin{array}{c}\mathbb{E}{S}_{12}=\mathbb{E}{S}_{12}^{\prime}+\sum\limits_{i=1}^{{n}_{1}-1}\sum\limits_{j=1}^{{n}_{2}-1}{\mathbb{E}}\left({S}_{i,j}\left({n}_{1},{n}_{2}\right)\right)\end{array}$$

7 $$\begin{array}{c}\mathbb{E}D=\mathbb{E}\left({S}_{0,{n}_{2}}\left({n}_{1},{n}_{2}\right)\right)+\mathbb{E}\left({S}_{{n}_{1,}0}\left({n}_{1},{n}_{2}\right)\right)\end{array}$$

where ${\mathbb{E}}{S}_{12}^{\prime}$ is the expected number of recurrent mutations that occur at the same locus simultaneously in the two species since the divergence time. Note that the above equations are valid for populations with constant sizes and work for small samples from each population, making them applicable for most existing comparative population genomic data. For samples with large sizes or from populations with temporally variable sizes, the formulae from Chen and Chen (2013) can be adopted [21].

### Modeling lineage-specific positive selection and balancing selection

The above equations are for polymorphism patterns under neutrality. The hitchhiking effect of one-wave directional selection can be modeled by approximating the hitchhiking effect with a sampling formula [20] or a linear transformation [22] or by assuming that the sites are causal mutants under direct selection [23]. CEGA focuses on the numbers of segregating and fixed sites under recurrent selective sweeps. We use the two scale coefficients ${\lambda }_{1}$ and ${\lambda }_{2}$ of ${N}_{1}$ and ${N}_{2}$ to model the effect of lineage-specific selection on genetic polymorphism and divergence. When recurrent positive selection acts on a gene locus in species $j$, the polymorphism level within species $j$ is reduced due to the hitchhiking effect, and the divergence between the two species is increased due to the increased fixation rate, which is similar to the pattern caused by a decreased effective population size in species $j$. We thus can model the effect of positive selection on genetic diversity and divergence by scaling the effective population size ${N}_{j}$ with a factor ${\lambda }_{j}^{l}<1.0$ for the selected locus. Former theoretical studies provided a detailed derivation of ${\lambda }_{j}$ as a function of recombination rate, selection intensity, and the frequency of beneficial mutations, which can be further used to infer these parameters of the underlying selective process ([24, 25] and others, see details in the section “Parametric inference of recurrent sweeps”). In contrast, balancing selection acting on locus $l$ can increase the polymorphism within species and decrease the divergence between species, resulting in a pattern identical to ${\lambda }_{j}>1.0$ (Fig. 1C). Under neutrality, ${\lambda }_{1}$ and ${\lambda }_{2}$ have a specific value of 1.

### Maximum likelihood inference and significant test

We employ a two-step approach for maximum likelihood inference of the parameters. In the first step, we assume ${\lambda }_{1}^{l}=1$ and ${\lambda }_{2}^{l}=1$. We estimate the global parameters of the model, including ${N}_{0}$, ${N}_{1}$, ${N}_{2}$, and ${T}_{d}$ with genome-wide data. In the second step, we focus on inferring the locus-specific parameters ${\lambda }_{1}^{l}$ and ${\lambda }_{2}^{l}$ and mutation rate ${\mu }^{l}$, and keep all the global parameters fixed at the value inferred in the first step. Further details on the likelihood functions can be found in the “Methods” section.

Two methods are used to assess the significance of a test. The first one, denoted as CEGA-$\lambda$, uses the genome-wide distribution of $\lambda$ values as the null distribution under neutrality. The distribution of $\lambda$ is skewed, and we employ Box-Cox transformation to align it with a standard normal distribution (Additional file 1: Figs. S1 and S2). The significance of $\lambda$ can be directly obtained from quantiles of the normal distribution (see details in the Supplementary information).

The second approach is to use the likelihood ratio test (CEGA-LRT, Additional file 1: Fig. S3). The null hypothesis is: ${\lambda }_{1}^{l},{ \lambda }_{2}^{l}=1,$ and ${\mu }^{l}$ is free. To test if species 1 is under selection, the alternative hypothesis is set to be: ${\lambda }_{2}^{l}=1,$ ${\lambda }_{1}^{l}$ and ${\mu }^{l}$ are free. To test if species 2 is under selection, the alternative hypothesis is: ${\lambda }_{1}^{l}=1,$ ${\lambda }_{2}^{l}$ and ${\mu }^{l}$ are free (see details in the Supplementary information). We compared the performance of the two significance tests on detecting selection signals. CEGA-$\lambda$ outperforms CEGA-LRT for both positive selection and balancing selection (Additional file 1: Figs. S4 and S5). The following analysis is based on the CEGA-$\lambda$ unless otherwise specified.

### Parametric inference of recurrent sweeps

In addition to detecting selection, it is of great interest to infer parameters related to the selective process, e.g., the intensity of selection acting on a local genomic region. $\lambda$ can be approximated with $\lambda \approx H/{H}_{neu}=N/{N}_{neu}$, the relative ratio of reduced effective population size attributed to selection and the effective population size under neutrality. Following [25–27] (see “Methods” for details), focusing on a selected mutant which is with *c* distance (recombination fraction, in units of Morgan) away from the focal neutral locus, we can obtain the reduction of expected heterozygosity due to the hitchhiking effect from a single selective sweep,

8 $$\begin{array}{c}h\left(c\right)=\frac{2c}{s}{\alpha }^{-2c/s}\Gamma \left(\frac{-2c}{s},\frac{1}{\alpha }\right)\end{array}$$

where *s* is the selection intensity, $\Gamma$ is the incomplete gamma function and $\alpha =2Ns$. $h\left(c\right)$ can be viewed as the probability of the neutral locus avoiding the hitchhiking effect by recombination during the selective sweep process. We can obtain ${k}_{h}\left(c\right)$, the expected number of selected substitutions that drag the neutral locus to fixation through hitchhiking effects in $2N$ generations,

9 $$\begin{array}{c}{k}_{h}\left(c\right)={2Nm}_{f}\left(1-h\left(c\right)\right)\end{array}$$

Here $N$ is the effective population size, ${m}_{f}$ is the expected number of fixed advantageous substitutions (per generation). For the coalescent process of two lineages of the focal neutral locus, the expected coalescent time is $1/\left(1+{K}_{h}\left(c\right)\right)$, which lead to the expected heterozygosity $H=4N\mu /\left(1+{k}_{h}\left(c\right)\right)$. $\lambda$, the mean of $H/{H}_{neu}$, can be obtained by averaging heterozygosity over the 2 ${L}^{\prime}$ neutral loci of the whole region,

10 $$\begin{array}{c}\lambda =\frac{1}{{L}^{\prime}}\sum\limits_{l=1}^{{L}^{\prime}}\frac{1}{1+{K}_{h}\left(l\rho \right)}\end{array}$$

where $\rho$ is the recombination rate per nucleotide. Equation 10 links $\lambda$ to a function of selection intensity and recombination rate of a focal region, providing the feasibility of inferring the selection intensity (a more detailed explanation can be found in “Methods”).

### Power to detect positive selection

**Fig. 2:**
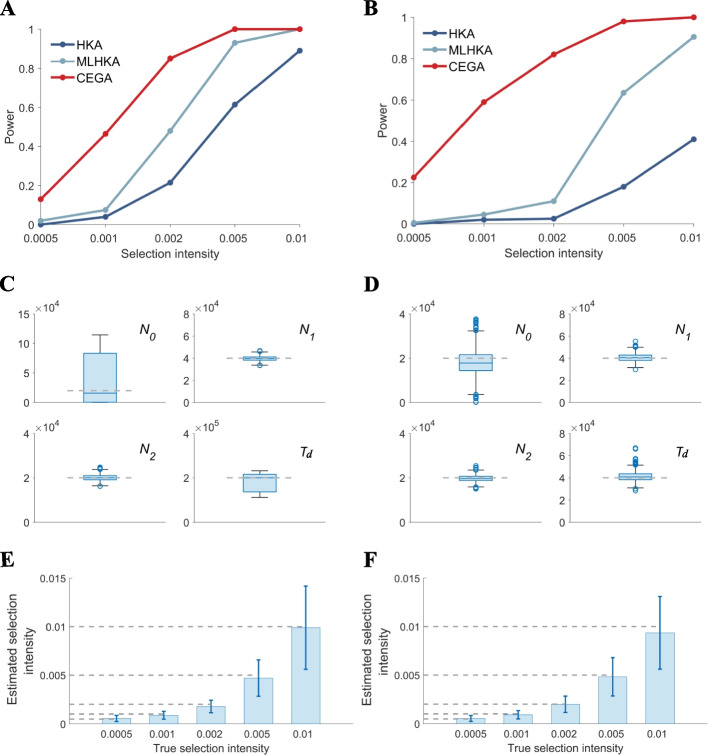
Performance of detecting positive selection. **A**, **B** Proportion of significant results as assessed by simulation with long-term divergence (200,000 generations ago, **A**) and short-term divergence (40,000 generations, **B**). **C**, **D** Accuracy of estimation of global parameters for simulations with long-term divergence (**C**) and with short-term divergence (**D**). **E**, **F** Accuracy of estimation of selection intensity for simulations with long-term divergence (**E**) and short-term divergence (**F**). The true values of parameters are indicated with dashed lines (**C**–**F**). Error bars correspond to standard deviations (**E**, **F**)

We evaluated the performance of CEGA in detecting positive selection using data simulated under different selection intensities and demographic histories (see details of simulation in the “Methods” section) and compared it with that of HKA and MLHKA. The results showed that CEGA outperforms HKA and MLHKA over the whole range of selection intensity values and under both scenarios with divergent times of 200,000 generations and 40,000 generations (Additional file 1: Figs. 2A and 2B). It is prominent that CEGA significantly outperforms the other two methods under low selection intensity and recent divergence (Fig. 2B). This is attributed to explicit modeling of the “ancient” segregating sites arising before the split of two populations by CEGA. Ancient sites are related to the four summary statistics ${S}_{1}$, ${S}_{2}$, ${S}_{12}$, and $D$ and are informative for inferring the parameters ${N}_{0}$, ${N}_{1}$, ${N}_{2}$, and ${T}_{d}$. This is especially important when the two species are closely related. As shown in Additional file 1: Figs. 2C and D, the estimates of ${N}_{0}$ and ${T}_{d}$ are much more accurate for scenarios with recent divergence than those with deep divergence.

### Inference accuracy of selection intensity for recurrent sweeps

To evaluate the performance of CEGA in estimating selection intensity, we conducted a new forward simulation with recurrent sweeps. The selected locus is with the length of 50 bp located in the middle of the whole segment, and the neutral region is with the length of 2 ${L}^{\prime}=$ 10 kb. The other parameters are kept consistent with previous simulations (see “Methods” for more details). Two hundred samples were generated for each selection intensity level. As shown in Additional file 1: Figs. 2E and F, CEGA provides unbiased and relatively precise estimates of selection intensity for both two scenarios with deep and recent divergence time. The results demonstrate that the theoretical model of recurrent sweeps effectively characterizes the hitchhiking effect on the reduction of genetic heterozygosity [25, 27]. However, it should be noted that the inferred selection intensity values exhibit a large variance for large *s*, which is likely attributed to the randomness of the number of fixed advantageous substitutions ${m}_{f}$. Overall, in addition to serving as a test for natural selection, CEGA also enables efficient inference of selection intensity.

### Power to detect balancing selection

**Fig. 3:**
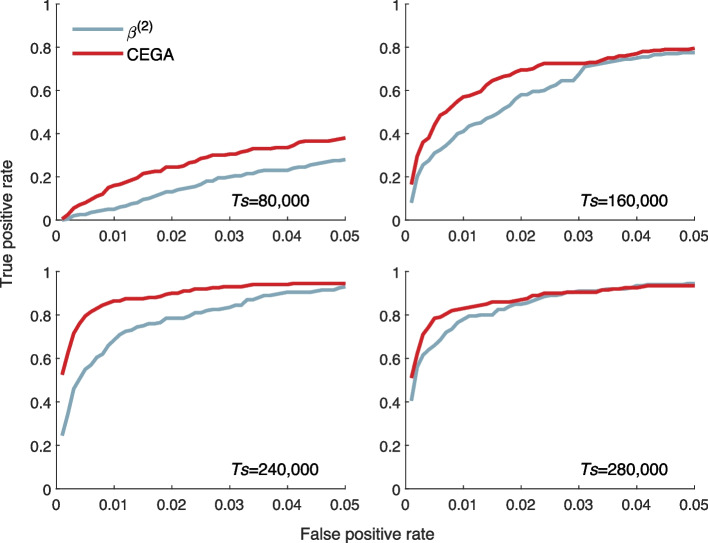
ROC curves of CEGA and BetaScan2 for detecting balancing selection signals at different selection onset times *Ts* = 80,000 and 160,000 (after the species split) and *Ts* = 240,000 and 280,000 (before the species split). The other parameters are selection intensity $s=0.001$ and overdominance coefficient $h=2$; the haploid sample sizes are ${n}_{1}={n}_{2}=20$

We compare the performance of CEGA with that of ${\beta }^{\left(2\right)}$ (implemented with BetaScan2) in detecting balancing selection (see the “Simulation” section) since ${\beta }^{\left(2\right)}$ has higher power to detect balancing selection than other existing methods, including HKA, T1, and ${\beta }^{\left(1\right)}$ [19]. The data are generated with the procedures in the “Methods” section. We set a window with a size of 2 kb for running CEGA. We maximized the performance of ${\beta }^{\left(2\right)}$ by assuming the values of all parameters except the selection coefficient; e.g., divergence time and the mutation rate were the true values, and the unfolded allele frequencies of single-nucleotide polymorphism (SNP) loci were known without uncertainty. In Fig. 3, we show the receiver operating characteristic (ROC) curves for scenarios with the selection coefficient *s* = 0.001 and the overdominance coefficient $h=2$. CEGA-InSel outperforms ${\beta }^{\left(2\right)}$ in all four scenarios with different selection onset times, including *Ts* = 80,000 and 160,000 for selection beginning after the species split and *Ts* = 240,000 and 280,000 for selection beginning before the species split. The mean power under a 1% false positive rate (FPR) is 0.6062 for CEGA and 0.4813 for ${\beta }^{\left(2\right)}$. The power under 1% FPR for the four scenarios is 0.16, 0.57, 0.865, and 0.83 for CEGA and 0.05, 0.41, 0.685, and 0.78 for ${\beta }^{\left(2\right)}$, respectively. More results of comprehensive simulations with different parameters can be found in Additional file 1: Fig. S7.

### Adaptive evolution in the human lineage

We applied CEGA to whole-genome sequencing data from nine *Homo sapiens* and nine *Pan troglodytes* [28]. The whole genome was divided into 2,416,717 windows with a window size of 10 kb and a step size of 1 kb. The effective population sizes of ancient species, humans, and chimpanzees are inferred to be ${N}_{0}=\mathrm{24,001}$, ${N}_{h}=\mathrm{21,369}$, and ${N}_{c}=\mathrm{29,461}$, respectively, and the divergence time is ${T}_{d}=\mathrm{214,935}$ generations ago. The ratio of ${N}_{c}/{N}_{h}=1.3787$ is close to 1.3978 estimated by Zhao et al. [7]. A total of 6937 and 7446 windows were identified as being under positive selection in the human and chimpanzee lineages, and correspondingly, 1145 and 1081 genes (including the 10 kb upstream regions) overlapped with the positively selected windows.

Gene-set enrichment analysis of the 1145 human-specific positively selected genes was performed with KOBAS-i [29], and the results are shown in Table 1. The significant level of enrichment of pathways or gene sets is evaluated by KOBAS-i using the hypergeometric test and Fisher’s exact test. The number of background genes used in Homo sapiens is 39,244. The most significant pathway is gene expression (transcription), with a corrected *p* value of $2.74\times {10}^{-7}$, including 84 significantly selected genes. Multiple top significantly enriched pathways are related to gene expression, including generic transcription (73 genes, corrected *p* value $=4.28\times {10}^{-7}$), RNA polymerase II transcription (76 genes, corrected *p* value $=1.93\times {10}^{-6}$), and transcriptional regulation by TP53 (26 genes, corrected *p* value = 0.0023) (Additional file 2: Table S2). This is consistent with previous studies showing that evolutionary changes in gene expression regulation played an essential role in the origin and development of *Homo sapiens* [7, 30, 31].

**Table 1:** Enrichment analysis of genes under positive selection in humans

Term	Input (Background)	Corrected *p* value
**Pathway (top 15 terms)**		
Gene expression (transcription)	84 (1448)	2.74E − 07
Immune system	109 (2096)	2.74E − 07
Generic transcription pathway	73 (1193)	4.28E − 07
RNA polymerase II transcription	76 (1316)	1.93E − 06
Metabolism	103 (2075)	6.27E − 06
Adaptive immune system	48 (748)	5.72E − 05
Metabolic pathways	74 (1433)	1.25E − 04
Chemical carcinogenesis	13 (82)	3.28E − 04
Metabolism of xenobiotics by cytochrome P450	12 (76)	7.49E − 04
Metabolism of proteins	91 (2012)	9.01E − 04
Post-translational protein modification	69 (1412)	1.13E − 03
Innate immune system	55 (1043)	1.28E − 03
Drug metabolism—cytochrome P450	11 (72)	1.89E − 03
Cell cycle	38 (629)	1.89E − 03
Transcriptional regulation by TP53	26 (359)	2.34E − 03
**Disease (top 15 terms)**		
Schizophrenia	20 (181)	1.37E − 04
Obesity-related traits	44 (691)	1.87E − 04
Longevity	9 (32)	2.44E − 04
Platelet counts	11 (77)	2.91E − 03
Age-related macular degeneration	10 (64)	3.29E − 03
Alzheimer’s disease	8 (47)	8.16E − 03
Other diseases	17 (204)	8.33E − 03
Mental and behavioral disorders	16 (189)	9.83E − 03
Alzheimer’s disease (late onset)	8 (50)	1.00E − 02
Autism	5 (15)	1.00E − 02
Metabolite levels (HVA/MHPG ratio)	5 (17)	1.45E − 02
Congenital disorders of metabolism	36 (695)	2.06E − 02
Gambling	4 (10)	2.10E − 02
Nervous system diseases	42 (859)	2.11E − 02
Bipolar disorder	12 (131)	2.39E − 02
**GO (top 15 terms with < 500 background genes)**		
Glutamatergic synapse	27 (354)	9.44E − 04
Signaling receptor activity	20 (214)	9.90E − 04
Neuron projection	26 (336)	1.04E − 03
Transcription factor binding	25 (325)	1.58E − 03
Glucuronosyltransferase activity	7 (23)	1.71E − 03
Brain development	20 (231)	2.24E − 03
Ubiquitin protein ligase binding	23 (294)	2.34E − 03
Protein kinase binding	30 (461)	3.39E − 03
Amyloid-beta binding	11 (80)	3.55E − 03
Neuronal cell body	26 (376)	4.17E − 03
Neuron projection development	13 (116)	4.72E − 03
Postsynaptic density	20 (251)	5.02E − 03
Flavone metabolic process	4 (5)	5.45E − 03
Chaperone cofactor-dependent protein refolding	7 (32)	6.36E − 03
mRNA 3'-UTR binding	10 (73)	6.69E − 03

1145 genes (with upstream 10 kb) that overlap with positively selected windows (*p* value < 0.0005) are included in the enrichment analysis. Only the top terms from the enrichment results are shown in the table, and the full list of terms is shown in Additional file 2: Tables S2-S4

Immune system-related pathways are another class of pathways enriched for human-specific positive selection signals, including immune system (109 genes, corrected *p* value $2.74\times {10}^{-7}$), adaptive immune system (48 genes, corrected *p* value $5.72\times {10}^{-5}$), innate immune system (55 significant genes, corrected *p* value 0.0013), immunoregulatory interactions between a lymphoid and a nonlymphoid cell (12 significant genes, corrected *p* value 0.0209), B-cell receptor signaling pathway (9 genes, corrected *p* value 0.0307), and signaling by interleukins (32 genes, corrected *p* value 0.0329) (Additional file 2: Table S2). The adaptive evolution of genes of the immune system may be driven by exposure and resistance to human-specific pathogens.

The third class of significantly enriched pathways is related to metabolism (103 genes, corrected *p* value $=6.27\times {10}^{-6}$), including metabolism of xenobiotics by cytochrome P450 (12 genes, corrected *p* value $=7.49\times {10}^{-4}$), proteins (91 genes, corrected *p* value $=9.01\times {10}^{-4}$), drug metabolism—cytochrome P450 (11 genes, corrected *p* value 0.0019), drug metabolism-other enzymes (11 genes, corrected *p* value 0.0034), porphyrin and chlorophyll (8 genes, corrected *p* value = 0.0051), steroid hormone biosynthesis (9 genes, corrected *p* value = 0.0076), pentose and glucuronate interconversions (7 genes, corrected *p* value = 0.0079), lipids (39 genes, corrected *p* value = 0.0089), and glucuronidation (6 genes, corrected *p* value = 0.0100) (Additional file 2: Table S2). These metabolism-related pathways cover a wide range of physiological processes. Some may be of fundamental function, and some may reflect human evolution driven by the shifts in diet and nutrition during the process of hominin evolution.

We found that multiple enriched terms in the disease category may reflect the specific cognitive features of humans compared to other living apes. These terms include schizophrenia (20 genes, corrected *p* value $1.37\times {10}^{-4}$), Alzheimer’s disease (8 genes, corrected *p* value 0.0082), mental and behavioral disorders (16 genes, corrected *p* value 0.0098), Alzheimer’s disease (late onset) (8 genes, corrected *p* value 0.0100), autism (5 genes, corrected *p* value 0.0100), nervous system diseases (42 genes, corrected *p* value 0.0211), bipolar disorder (12 genes, corrected *p* value 0.0239), and Alzheimer’s disease (cognitive decline) (6 genes, corrected *p* value 0.0629) (Additional file 2: Table S3). Notably, among the gene ontology (GO) terms, the human-specific positively selected genes are enriched in brain development (20 genes with a corrected *p* value of 0.0022, Additional file 2: Table S4 and Additional file 1: Figs. S13-S32), which included *B3GNT5, CCDC39, CASP2, IMMP2 L, ADGRL3, NFIB, SYT1, KCNAB1, MEIS2, AK4, PTPRG, CLN5, CNTNAP2, PITPNM1, MACROD2, TMX2, MTOR, OXCT1, PBX2*, and *ATXN1*, serving as an interesting candidate list for further functional investigation. Consistent with the pathway enrichment results, the terms of diseases related to metabolism are also significant, including obesity-related traits and congenital disorders of metabolism (Additional file 2: Table S3).

### Adaptive evolution in the noncoding regions of the human lineage

A subset of 342 genes under positive selection showed signals only in the noncoding regions (Additional file 2: Table S5). We further performed gene enrichment analysis on these genes (Table 2, Additional file 2: Tables S6-S8). Interestingly, multiple significant terms in pathway, disease, and GO categories are related to the brain and nervous system, including neuronal system (13 genes, corrected *p* value 0.0066), dopaminergic synapse (6 genes, corrected *p* value 0.0325), schizophrenia (9 genes, corrected *p* value 0.0058), Alzheimer disease (cognitive decline) (4 genes, corrected *p* value 0.0300), brain connectivity (2 genes, corrected *p* value 0.0458), learning or memory (6 genes, corrected *p* value 0.0054), synapse (14 genes, corrected *p* value 0.0054), postsynapse (7 genes, corrected *p* value 0.0054), regulation of neuron apoptotic process (4 genes, corrected *p* value 0.0055), neuron projection (12 genes, corrected *p* value 0.0062), GABA-ergic synapse (6 genes, corrected *p* value 0.0062), brain morphogenesis (4 genes, corrected *p* value 0.0066), dendrite (13 genes, corrected *p* value 0.0079), axon cytoplasm (5 genes, corrected *p* value 0.0128), postsynaptic membrane (7 genes, corrected *p* value 0.0159), vocalization behavior (3 genes, corrected *p* value 0.0222), neurotransmitter receptor activity (5 genes, corrected *p* value 0.0301), and anchored component of presynaptic membrane (2 genes, corrected *p* value 0.0379). The above terms are associated with 69 unique genes.

**Table 2:** Enrichment analysis of the noncoding regions of genes under positive selection in humans

Term	Input (Background)	Corrected *p* value
**Pathway (top 10 terms)**		
Metabolism	36 (2075)	6.23E − 03
Porphyrin and chlorophyll metabolism	5 (42)	6.23E − 03
Neuronal system	13 (402)	6.60E − 03
Retrograde endocannabinoid signaling	8 (148)	6.60E − 03
EGF receptor signaling pathway	7 (114)	7.40E − 03
Signaling by interleukins	16 (619)	9.10E − 03
Diseases of signal transduction	12 (374)	9.63E − 03
Cell cycle	16 (629)	1.01E − 02
Oocyte meiosis	7 (128)	1.17E − 02
Negative regulation of the PI3K/AKT network	6 (96)	1.49E − 02
**Disease (top 10 terms)**		
Obesity-related traits	21 (691)	4.41E − 04
Schizophrenia	9 (181)	5.76E − 03
Height	12 (395)	1.31E − 02
Immune response to smallpox (secreted IL-2)	3 (13)	1.69E − 02
Myocardial infarction (early onset)	3 (14)	1.92E − 02
Metabolite levels (HVA/MHPG ratio)	3 (17)	2.61E − 02
Bone mineral density (hip)	3 (18)	2.87E − 02
Alzheimer’s disease (cognitive decline)	4 (46)	3.00E − 02
Renal function-related traits (BUN)	3 (19)	3.00E − 02
Bone mineral density	5 (85)	3.16E − 02
**GO (top 20 terms with < 500 background genes)**		
Learning or memory	6 (60)	5.45E − 03
Transmitter-gated ion channel activity involved in regulation of postsynaptic membrane potential	5 (35)	5.45E − 03
Synapse	14 (420)	5.45E − 03
Postsynapse	7 (93)	5.45E − 03
Regulation of neuron apoptotic process	4 (17)	5.54E − 03
Regulation of translation	6 (63)	5.54E − 03
Myoblast differentiation	4 (18)	5.72E − 03
Transcription factor binding	12 (325)	5.72E − 03
Flavone metabolic process	3 (5)	5.72E − 03
Neuron projection	12 (336)	6.23E − 03
GABA-ergic synapse	6 (70)	6.23E − 03
Flavonoid glucuronidation	3 (6)	6.23E − 03
Brain morphogenesis	4 (21)	6.60E − 03
Coumarin metabolic process	3 (7)	7.40E − 03
Glucuronosyltransferase activity	4 (23)	7.40E − 03
Neuron projection development	7 (116)	7.92E − 03
Dendrite	13 (420)	7.92E − 03
Xenobiotic glucuronidation	3 (8)	8.26E − 03
Positive regulation of catalytic activity	6 (82)	8.82E − 03
Gamma-aminobutyric acid signaling pathway	4 (27)	1.01E − 02

342 genes with promoter (10 kb upstream) or intron regions overlapping with positively selected windows (*p* value < 0.0005) are included in the enrichment analysis (excluding 639 genes that also overlap with exons). Only the top terms in the enrichment results are shown in the table, and the full list of terms is shown in Additional file 2: Tables S5-S8

Among these genes, *MAD1L1* (Additional file 1: Fig. S33) shows significant selection signals in intron 18 with the data pattern ${S}_{1}=0$, ${S}_{2}=65$, ${S}_{12}=0$, *D* = 139, ${\lambda }_{1}<0.001$, *p* value $<{10}^{-20}$. *MAD1L1* is known as human accelerated region 3 and is one of the 49 human genomic segments that are conserved throughout vertebrate evolution but starkly divergent in the human lineage and thus may have played a key role in human evolution [32, 33]. Genome-wide association studies (GWASs) indicate that *MAD1L1* is related to multiple traits, including self-reported educational attainment, bipolar disorder, and schizophrenia [34–38]. The intronic SNP rs11764590 of *MAD1L1* is associated with bipolar disorder via functional alterations in the reward system [35], an intermediate phenotype for bipolar disorder. rs4236274 and rs4332037 in the intron regions of *MAD1L1* have also been reported to be significantly associated with bipolar disorder in GWASs [34, 36, 37].

The peak of − log10(*p* value) in *ZEB2* overlaps with the promoter/enhancer GH02J144502 (Additional file 1: Fig. S34) [39]. Compared with that of other apes, the brain of humans features a large volume, which is approximately 3.5 times larger than that of the chimpanzee brain [40]. A recent study revealed that *ZEB2* is responsible for the difference in the duration of brain expansion in humans and other great apes, leading to a larger brain in humans [41].

Four mTOR-related genes associated with intracranial volume and intellectual disability [42], namely, *PPP2R5A, PPP2R5C, AKT2*, and *MTOR*, are among the top list of positively selected genes in the human lineage (Additional file 1: Figs. S35-S37, and S29). Specifically, *PPP2R5A, PPP2R5C*, and *AKT2* show selection signals only in the regulatory regions. mTOR-controlled signaling pathways regulate many integrated physiological functions of the nervous system, e.g., neuronal development, synaptic plasticity, memory storage, and cognition. mTOR signaling is also known to be associated with autism and other neurological and psychiatric disorders, suggesting its role in the recent evolution of the human brain [43–46]. In some recent single-cell and organoid studies, mTOR signaling was proven to regulate the morphology of outer radial glia in the development of the human cerebral cortex, which is a critical component of the human brain [47, 48].

**Fig. 4:**
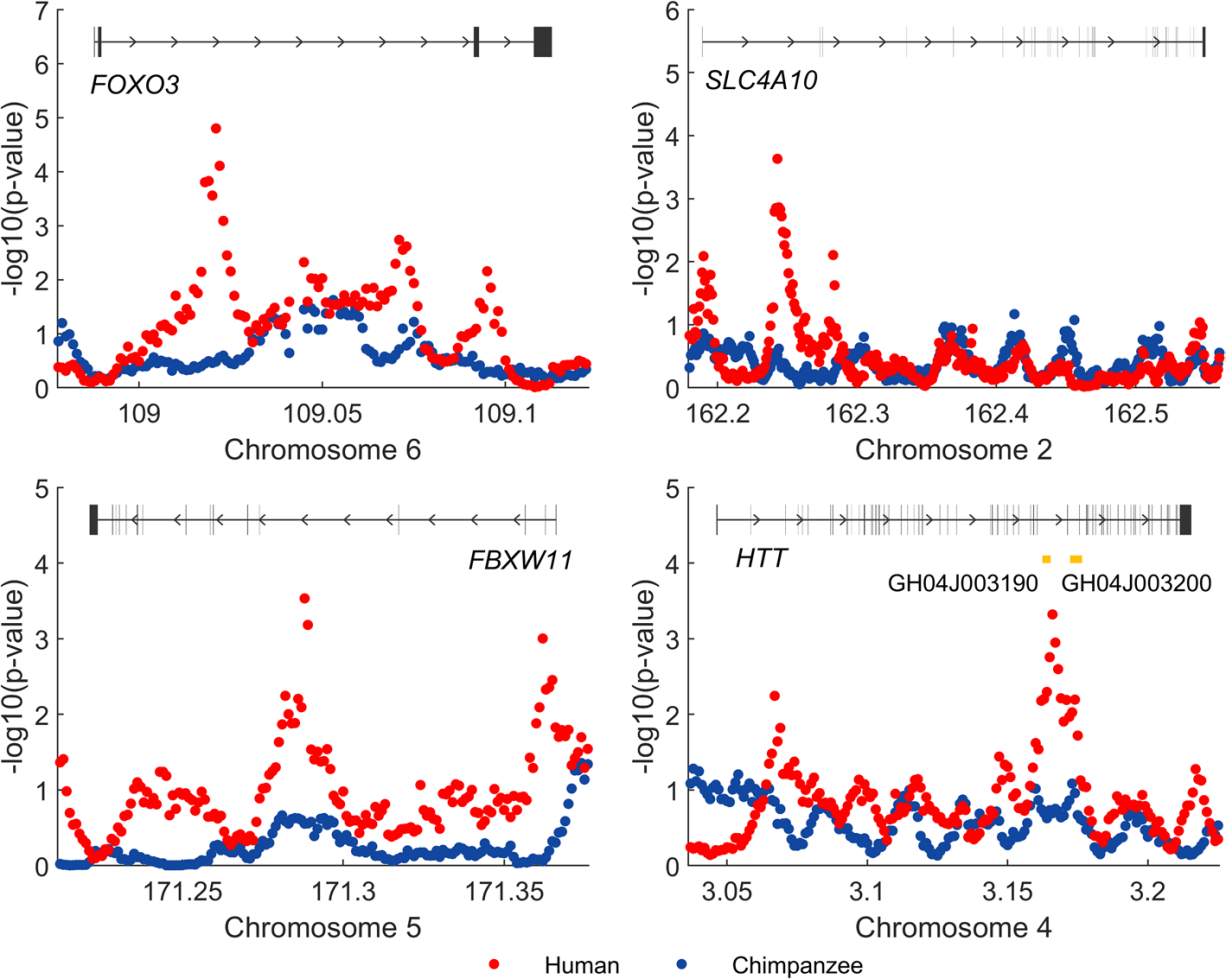
Four genes of the brain morphogenesis pathway show signals of positive selection only in noncoding regions in the human lineage. This pathway is significant in the gene-set enrichment analysis, with a corrected *p* value of 0.0066. Red dots: − log10(*p* value) of normalized $\lambda$ values in humans; blue dots: − log10(*p* value) of normalized $\lambda$ values in chimpanzees. Top panel: gene structure annotation and identified promoter and enhancers

Adaptive evolution in noncoding regions of the human genome may play important roles in shaping human brain morphogenesis. Four genes, namely, *FOXO3*, *SLC4A10*, *HTT*, and *FBXW11*, were identified as being under positive selection in the human lineage (Fig. 4), with a corrected *p* value of 0.0066 for the gene-set enrichment analysis. In contrast, there is no evidence of accelerated evolution within the noncoding regions of these genes in chimpanzees after the split of the two species.

*FOXO3* may help regulate the long-term regenerative potential of neural stem/progenitor cells (NSPCs) under age- or injury-related brain environmental changes such as elevated oxidative stress [49]. *FOXO3* is related to brain weight, according to mouse experiments [50, 51], probably by affecting the neural stem cell pool. The cerebral cortex underlies the higher-order cognition of humans, and GWASs indicate that intronic variants of *FOXO3* are correlated with the surface area of the human cerebral cortex [52, 53], cortical thickness [53, 54], brain volume [55], vertex-wise sulcal depth [54], intelligence [56, 57], and schizophrenia [58, 59].

*SLC4A10* plays an essential role in regulating the intracellular pH of neurons, the secretion of bicarbonate ions across the choroid plexus, and the pH of the brain extracellular fluid. Physiology and behavior, such as synaptic plasticity, learning, and neurodegeneration, can be dramatically altered through pH-sensitive receptors and channels when pH fluctuates [60–64]. Significantly decreased expression of *SLC4A10* helps explain reduced cerebrospinal fluid (CSF) formation and turnover in Alzheimer’s disease (AD), resulting in impaired clearance of toxic metabolites and neuroinflammation [65]. Moreover, *SLC4A10* knockout (KO) mice have decreased brain ventricle sizes, indicating reduced CSF production [66, 67]. The human brain is characterized by its high metabolic cost, consuming approximately 20% of oxygen intake while accounting for only 2% of body mass [68, 69]. Metabolic intensity may coevolve with a pH-regulating capacity, resulting in positive selection of genes such as *SLC4A10*. Variants of *SLC4A10* correlate with cognitive performance [70], cortical surface area [71], etc.

*FBXW11* is one of the top differentially expressed genes in the prefrontal cortex between AD cases and controls and is among the hub genes in the protein‒protein interaction network [72]. Genetic variation in *FBXW11* correlates with cortical surface morphology [73]. *HTT* correlates with Huntington’s disease, a neurodegenerative disorder. The knockdown of *HTT* in neuroepithelial cells of the neocortex results in disturbed cell migration, reduced proliferation, and increased cell death [74]. GWASs demonstrate that genetic variations in *HTT* are associated with vertex-wise sulcal depth [54], mathematical ability [70], etc. The leading window among those with positive selection signals on *HTT* overlaps with the elite enhancer GH04J003190 [39].

### Balancing selection

Seventy-nine genes were identified as being under balancing selection in both humans and chimpanzees by CEGA. The results of KOBAS-i gene-set enrichment analysis (Table 3) demonstrate that immune system-related pathways are under long-term balancing selection, among which the pathway “translocation of *ZAP-70* to immunological synapse” (6 genes, corrected *p* value $=1.58\times {10}^{-9}$) is the most significantly enriched. This is consistent with the findings of previous studies [75].

**Table 3:** Enrichment analysis of genes under balancing selection in both the human and chimpanzee lineages

Term	Input (Background)	Corrected *p* value
**Pathway (top 15 terms)**		
Translocation of ZAP-70 to immunological synapse	6 (18)	1.58E − 09
Phosphorylation of CD3 and TCR zeta chains	6 (21)	2.20E − 09
PD-1 signaling	6 (22)	2.20E − 09
Generation of second messenger molecules	6 (32)	7.94E − 09
Olfactory transduction	11 (448)	5.21E − 08
Costimulation by the CD28 family	6 (65)	2.25E − 07
Asthma	5 (31)	2.83E − 07
Allograft rejection	5 (38)	6.23E − 07
Graft-versus-host disease	5 (41)	8.07E − 07
Interferon gamma signaling	6 (90)	8.19E − 07
**Disease (top 15 terms)**		
Lymphoma	4 (12)	6.85E − 07
Nephropathy	4 (19)	2.07E − 06
Cervical cancer	3 (8)	1.63E − 05
Alzheimer's disease (late onset)	4 (50)	4.36E − 05
Systemic sclerosis	3 (17)	9.19E − 05
Hypothyroidism	3 (34)	5.65E − 04
Dilated cardiomyopathy	3 (35)	6.03E − 04
Hepatitis B	2 (7)	1.46E − 03
Ulcerative colitis	4 (138)	1.64E − 03
Sjögren’s syndrome	2 (8)	1.67E − 03
**Go (top 10 terms with < 500 background genes)**		
MHC class II protein complex	6 (15)	1.28E − 09
Integral component of lumenal side of endoplasmic reticulum membrane	6 (28)	6.22E − 09
MHC class II receptor activity	5 (10)	7.08E − 09
Clathrin-coated endocytic vesicle membrane	6 (32)	7.94E − 09
Transport vesicle membrane	6 (41)	2.71E − 08
ER to Golgi transport vesicle membrane	6 (53)	8.39E − 08
Endocytic vesicle membrane	6 (66)	2.28E − 07
Interferon-gamma-mediated signaling pathway	6 (71)	2.83E − 07
Peptide antigen binding	5 (31)	2.83E − 07
Olfactory receptor activity	10 (427)	2.83E − 07

79 genes that overlap with windows under balancing selection (*p* value < 0.0005) in both the human and chimpanzee lineages are included in the enrichment analysis. Only the top terms in the enrichment results are shown in the table, and the full list of terms is shown in Additional file 2: Tables S14-S16

**Fig. 5:**
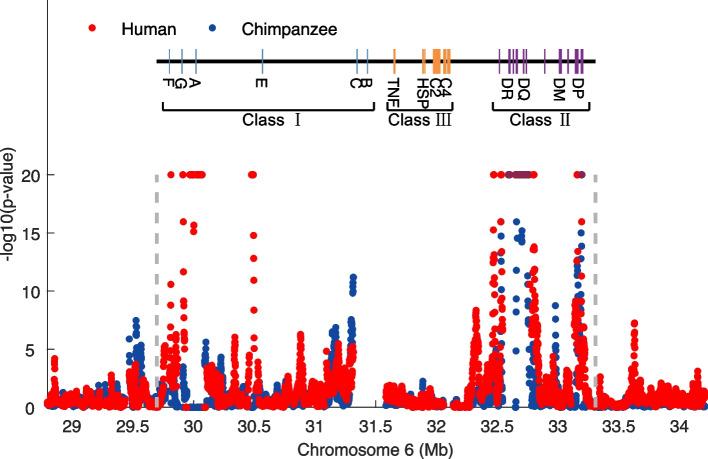
Balancing selection signals in the MHC region of humans and chimpanzees. Red dots: − log10(*p* value) of normalized $\lambda$ values of humans; blue dots: − log10(*p* value) of normalized $\lambda$ values of chimpanzees. Top panel: gene structure annotation

Specifically, we found strong evidence of balancing selection on MHC class II genes in humans (HLA genes) and chimpanzees (Patr genes) (Fig. 5). The shared genes under balancing selection in human and chimpanzee lineages are significantly enriched in the GO terms MHC class II protein complex (corrected *p* value $=1.28\times {10}^{-9}$) and MHC class II receptor activity (corrected *p* value $=7.08\times {10}^{-9}$). The highly polymorphic MHC alleles retained by balancing selection may be caused by the immune response to a wide range of pathogens [76]. The genes under balancing selection in humans are also significantly enriched in the GO term MHC class I protein complex (corrected *p* value = 0.0287 in humans, Additional file 2: Table S20). Interestingly, in chimpanzees, we instead detected a signature of strong positive selection around the Patr-A gene, which is the counterpart of human MHC I genes (*p* value < ${10}^{-20}$, Additional file 2: Table S9). Multiple lines of evidence indicate that chimpanzees experienced an ancient selective sweep leading to severe reduction of the MHC class I repertoire [77–80]. According to functional studies, HIV-1/SIV-like retrovirus may be responsible for the loss of diversity [81]. The positively selected chimpanzee genes are also significantly enriched in the reactome pathway HIV infection (corrected *p* values of 0.0741 in chimpanzees and 0.2627 in humans, Additional file 2: Tables S2 and S10).

In addition to MHC/HLA, several other genes also demonstrate signals of balancing selection in the noncoding regions. *IGFBP7* (Additional file 1: Fig. S38) shows significant balancing selection signals in introns with the data pattern ${S}_{1}=84,{S}_{2}=108$, ${S}_{12}=9$, $D=30$, ${\lambda }_{1}=3.87$, ${\lambda }_{2}=3.71$, with *p* values of $6.86\times {10}^{-5}$ in humans $1.15\times {10}^{-4}$ in chimpanzees. The region overlaps with the enhancer GH04J057050. *IGFBP7* was also identified as being under ancient balancing selection in previous research, with shared SNPs of humans and chimpanzees occurring in a likely enhancer [75]. *IGFBP7* plays a role in innate immunity [82] and can promote the formation of type II rosettes [83]. Another region showing significant signals of balancing selection in the human lineage in our study is the *ABO* groups (Additional file 1: Fig. S39), with the data pattern ${S}_{1}=107$, ${S}_{2}=27$, ${S}_{12}=1$, $D=52$, ${\lambda }_{1}=4.21$, *p* value $2.34\times {10}^{-5}$. The *ABO* locus has been hypothesized to be under balancing selection for a long time [84, 85].

## Discussion

There are several potential improvements that can be made to the current approach. Instead of using the four summary statistics for sequences from the two species, an alternative approach could be to utilize the conditional allele frequency spectrum of a species with two outgroups [86] or use the full joint allele frequency spectrum (JAFS) of multiple species ($\mathrm{p}$) [20], which contains ${n}^{p}$ entries of summary statistics and provides more information. However, the data fitting process becomes challenging, and computational intensity increases rapidly with the sample size. Another improvement worth considering is to extend the method to analyze the joint data pattern across multiple species, similar to the HDMKPRF method [7]. As we demonstrated in this paper, CEGA models the joint site pattern in two species, in contrast to MLHKA, which models the polymorphism in a single population, and boosts the power to detect selection by gaining more information. In addition to the increased power, this novel method can be extended to multiple populations allowing for pinpointing the occurrence of selection at different stages and in turn construct a temporal map of natural selection across multiple species.

CEGA approximates the demographic history of different species with constant effective population sizes. The approximation is reasonable since CEGA focuses on recurrent selective sweeps or balancing selection over a relatively long-term period. Simulations of five non-equilibrium demographic scenarios were carried out to evaluate the simplified model of CEGA, including ancient severe bottleneck, ancient mild bottleneck, recent severe bottleneck, recent mild bottleneck, and recent exponential growth (see Supplementary Section 6 for detailed parameter settings). As shown in Additional file 1: Figs. S8-S10, the polymorphic and divergent site patterns are approximately equivalent to those from constant-size model with the effective population size inferred using CEGA. This suggests the robustness of the method to simplified demographic history models. While approximating with constant effective population sizes works well for non-equilibrium demographic histories, CEGA is capable of accommodating more complex demographic models if it is necessary. A parametric model with changing population sizes can be fitted using the joint allele frequency spectrum methods ([20, 23, 87] and others), and then the expected values of the four summary statistics under the inferred demographic model can be obtained using former theoretical results and incorporated into the likelihood function of CEGA [21, 88].

Another practical issue arises from the fact that the method was developed based on the model assumption of random mating species and recombinant genomes. For certain species with distinct breeding histories, such as a selfing species, or species exhibiting very low or zero recombination rates, the method is not applicable.

CEGA is computationally efficient, making it feasible to apply to genome-wide data analysis. Additionally, CEGA provides multi-threaded mode, which allows for parallel processing and further improves the efficiency. As an example, it takes 8 h for CEGA to analyze the whole genome of nine Homo sapiens and nine Pan troglodytes with 2,416,717 sliding windows of 10 kb (using 40 threads, Intel(R) Xeon(R) Gold 6230 CPU @ 2.10GH).

The application of CEGA to the genomic data of humans and chimpanzees identified a list of genes under positive selection and balancing selection. Importantly, a subset of genes with signals only in the noncoding regions in the human lineage are significantly enriched in pathways related to the brain and nervous system, including brain morphogenesis, synapse activity, learning or memory, and brain disease, suggesting their critical roles in the development and functionality of human cognition. This set of genes serves as a foundation for further investigation, which may provide insights into the origin of human-specific phenotypes.

## Conclusions

A comparative population genomic method, CEGA, is developed for detecting directional selection and balancing selection using both within-species genomic polymorphism and between-species divergence. CEGA is based on the HKA framework and the JAFS from coalescent theory [20]. Although multiple methods have been developed for evolutionary comparative genomic analysis, CEGA complements existing methods with multiple advantages. For example, CEGA does not assume models of protein codon substitution, making it applicable to analyses of both coding regions and noncoding regions, and thus it is especially useful for investigating the evolution of regulatory regions. CEGA also has higher power than existing methods over a wide range of selection intensity values for populations with ancient and recent divergence times. Furthermore, it provides inferred parameters of the evolutionary process. CEGA is computationally efficient and can be used to analyze large samples of genomic data. CEGA thus provides a useful tool for analyzing population genomic data from two species or populations.

## Methods

### Two-step maximum likelihood estimation of parameters

After the derivation of expected values of ${\mathbb{E}}{S}_{1}$, ${\mathbb{E}}{S}_{2}$, ${\mathbb{E}}{S}_{12}$, and ${\mathbb{E}}D$, we infer model parameters and detect natural selection by implementing the following two-step maximum likelihood estimation. In the first step, we estimate the global model parameters ${N}_{0}$, ${N}_{1}$, ${N}_{2}$ and ${T}_{d}$ by maximizing the likelihood function,

11 $$\begin{aligned}L\left({N}_{0},{N}_{1},{N}_{2},{T}_{d}\left|{S}_{1},{S}_{2},{S}_{12},D\right.\right) &= \prod\limits_{l=1}^{L}\mathrm{Pr}\left({S}_{1}^{l}\left|{\mathbb{E}}{S}_{1}^{l}\right.\right){\text{Pr}}\left({S}_{2}^{l}\left|{\mathbb{E}}{S}_{2}^{l}\right.\right)\\ &\qquad {\text{P}}{\text{r}}\left({S}_{12}^{l}\left|{\mathbb{E}}{S}_{12}^{l}\right.\right){\text{P}}{\text{r}}\left({D}^{l}\left|{\mathbb{E}}{D}^{l}\right.\right)\end{aligned}$$

where ${\text{Pr}}\left(\cdot \right)$ denotes the probability function of the Poisson distribution.

When estimating the global parameters using Eq. 11, we assume that the global mutation rate is known, and ${\lambda }_{1}^{l}=1$ and ${\lambda }_{2}^{l}=1$ are set for all loci. After the global parameters are inferred, we implement the second step to estimate the locus-specific parameters ${\lambda }_{1}^{l}$ and ${\lambda }_{2}^{l}$ and mutation rate ${\mu }^{l}$ by maximizing the likelihood function over the three parameters with the other parameters fixed to values inferred in the first step,

12 $$\begin{array}{c}L\left({\lambda }_{1}^{l},{\lambda }_{2}^{l},{\mu }^{l}\left|{S}_{1}^{l},{S}_{2}^{l},{S}_{12}^{l},{D}^{l},{N}_{0}={\widehat{N}}_{0},{N}_{1}={\widehat{N}}_{1},{N}_{2}={\widehat{N}}_{2},{T}_{d}={\widehat{T}}_{d}\right.\right)\\ ={\text{P}}{\text{r}}\left({S}_{1}^{l}\left|{\mathbb{E}}{S}_{1}^{l}\right.\right){\text{P}}{\text{r}}\left({S}_{2}^{l}\left|{\mathbb{E}}{S}_{2}^{l}\right.\right){\text{P}}{\text{r}}\left({S}_{12}^{l}\left|{\mathbb{E}}{S}_{12}^{l}\right.\right){\text{P}}{\text{r}}\left({D}^{l}\left|{\mathbb{E}}{D}^{l}\right.\right),{\text{f}}{\text{o}}{\text{r}}1\le l\le L\end{array}$$

where ${\mathbb{E}}{S}_{1}^{l}$, ${\mathbb{E}}{S}_{2}^{l}$, ${\mathbb{E}}{S}_{12}^{l}$, and ${\mathbb{E}}{D}^{l}$ are calculated with Eqns. 4, 5, 6, and 7 as a function of ${\lambda }_{1}^{l}{N}_{1}$, ${\lambda }_{2}^{l}{N}_{2}$ and the locus-specific mutation rate ${\mu }^{l}$.

### Parametric inference of selection intensity of recurrent selective sweeps

In this section, we show how to connect the parameter $\lambda$ with the selection intensity of recurrent selective sweeps acting on a genomic region. $\lambda$ is the ratio of effective population size under recurrent selective sweeps to the effective population size under neutrality. We consider a genomic segment of length $2{L}^{\prime}+{m}_{s}$ bases that undergoes recurrent selective sweeps, and beneficial mutations occur at the ${m}_{s}$ bases located in the middle of the segment. All beneficial mutations are with fixed selection intensity of *s* (the heterozygote individuals are with the fitness of 1 + *s*). These assumptions can be easily extended to more general cases. We assume that ${m}_{s}$ is small enough to avoid fixation of multiple advantageous mutants simultaneously. Under the above assumptions, $\lambda$ can be derived as a function of selection intensity *s* of recurrent selective sweeps following previous studies [25–27].

First, for a neutral locus linked to a selected mutant, the reduction in the expected heterozygosity caused by the hitchhiking effect from a single selective sweep is [25, 27]

$$h\left(c\right)=\frac{2c}{s}{\alpha }^{-2c/s}\Gamma \left(\frac{-2c}{s},\frac{1}{\alpha }\right),$$

where *c* is the recombinational distance (in units of Morgan) between the neutral locus and the selected substitution, $\Gamma$ is the incomplete gamma function, *s* is the selection intensity, $\alpha =2Ns$, and *N* is the effective population size. $h\left(c\right)$ is equal to the “escape probability” that the neutral locus avoids the hitchhiking effect by recombination occurred between the neutral locus and the selected mutant during the selective sweep process [25].

We further investigate the hitchhiking effects from recurrent sweeps. Since the fixation probability of a new advantageous allele under selection is approximately [89]

13 $$\begin{array}{c}{p}_{f}=\frac{1-{e}^{-2s}}{1-{e}^{-4Ns}}\end{array}$$

The expected number of fixed advantageous substitutions (per generation) within the local segment is

14 $$\begin{array}{c}{m}_{f}=2N\mu {m}_{s}{p}_{f}\end{array}$$

where $\mu$ denotes the mutation rate per nucleotide site per generation. We consider the accumulated effect of these fixed advantageous substitutions at the neutral locus. Then, the expected number of selected substitutions (per 2*N* generations) that drag the neutral locus to fixation is

$${k}_{h}\left(c\right)={2Nm}_{f}\left(1-h\left(c\right)\right).$$

In the coalescent framework, as the process traces back in time, the occurrence rate of a coalescent or a hitchhiking event of the neutral locus is ${1+k}_{h}\left(c\right)$ per 2*N* generations. The expected coalescent time is then ${1/(1+k}_{h}\left(c\right))$. Since the expected heterozygosity *H* is known to be the probability of observing two distinct alleles in the two copies of the neutral locus), we have

15 $$\begin{array}{c}H=2\times 2Nu\times \frac{1}{1+{k}_{h}\left(c\right)}=\frac{4Nu}{1+{k}_{h}\left(c\right)}\end{array}$$

$\lambda$ of the single neutral locus can be approximately equal to $H/{H}_{neu}=1/\left(1+{k}_{h}\right)$. Finally, the expected value of $\lambda$ is the mean of $H/{H}_{neu}$ across all sites within the local segment [25, 27]:

$$\lambda =\frac{1}{{L}^{\prime}}{\sum }_{l=1}^{{L}{\prime}}\frac{1}{1+{K}_{h}\left(l\rho \right)},$$

where $\rho$ denotes the recombination rate per nucleotide site per generation.

The above results can be easily extended to recurrent selective scenarios with more general assumptions, e.g., the selected substitutions occurring randomly along the whole region (see [25]).

### Simulation

Genomic sequences were simulated using the forward simulator SLiM 3.6 [90]. We simulated genomic data under two scenarios of demographic history for the three species shown in Fig. 1A. In scenario I, the simulation process started from the common ancestor of human, chimpanzee, and gorilla (${N}_{a}$), with 100,000 generations of burn-in to achieve the equilibrium state. The common ancestor of humans and chimpanzees (${N}_{a}$) existed for 120,000 generations with an effective population size of ${N}_{0}=\mathrm{10,000}$. After that, it split into two species, humans and chimpanzees, with effective population sizes of ${N}_{h}=\mathrm{10,000}$ and ${N}_{c}=\mathrm{20,00}0$, respectively. The two species then evolved for another 200,000 generations. Twenty chromosomes were randomly sampled from each species. In scenario II, all the demographic parameters were identical to those in scenario I except that the split time of the two species was 40,000 generations ago (see details of the forward simulations in the Supplementary information). Scenarios I and II correspond to distantly related species and closely related species, respectively.

Three types of genome segments of 100 kb (under neutrality, positive selection and balancing selection) were simulated with a point mutation rate of $\mu =2.5\times {10}^{-8}$ per bp and a recombination rate of $1\times {10}^{-8}$ per bp. For a positively selected segment, 1% of the new mutations were set to be beneficial. For a segment under balancing selection, one variant under balancing selection is located at the center of the segment.

For positive selection, data were simulated with five different selection intensities *s* = 0.0005, 0.001, 0.002, 0.005, and 0.01. The occurrence of positively selected mutants started 200,000 generations ago in scenario I and 40,000 generations ago in scenario II.

For balancing selection, the selection intensity was set to $s=0.001$. The overdominance coefficient of the mutation was set to *h* = 2. The selection onset times were 80,000 and 160,000 generations ago in the human lineage and 240,000 and 280,000 generations ago in the common ancestor lineage. If the mutation under selection was lost due to random sampling, the simulation process was restarted.

To evaluate the performance of CEGA in detecting positive selection, we integrated 19 neutral segments with one positively selected segment (we used 10-kb segments in the center of the simulated segments, the same below). Two hundred samples were generated for each selection intensity. MLHKA and CEGA were tested with the same data set. We used 20 segments since this is the maximum number of segments restricted by MLHKA, although CEGA can handle many more segments to obtain a more accurate estimate of the global model parameters.

Two hundred samples were generated for each selection onset time to evaluate the performance of CEGA in detecting balancing selection. For each simulated data set, 19 neutral segments were simulated together with one segment under balancing selection. We also tested the performance of CEGA on segments with different sizes, including 500 bp, 1 kb, 2 kb, 4 kb, 6 kb, and 10 kb. When evaluating the FPR of the methods, 20 neutral segments were generated for each simulated data set, and 1000 samples were generated.

### Implementation of MLHKA, HKA, and BetaScan2

MLHKA was downloaded from https://github.com/rossibarra/MLHKA [8]. The MCMC chain of MLHKA was run for 1,000,000 iterations, and the initial values of parameters were set to real values to accelerate the convergence of the chain. The convergence of MCMC was tested by comparing the results from several MCMC chains with different initial values of parameters and seeds.

The HKA test was implemented using the slightly modified version of Wang and Hey (1996), which was proven to have a higher power by testing the largest deviation values, regardless of species or locus and regardless of whether the observation corresponds to polymorphism or divergence [91].

BetaScan2 was downloaded from https://github.com/ksiewert/BetaScan [19]. Standardized Beta2 scores were calculated with the true divergence time and mutation rate (-B2 -DivTime 10.0 -std -Theta 0.001). The unfolded allele frequency with substitutions was analyzed by assuming that the ancestral states of mutations were known. We set a window size of 1000 bp (default value).

### Data filtering

We applied CEGA to genomic sequences of humans and chimpanzees from Prado-Martinez et al. [28]. The data were generated via next-generation sequencing (NGS) technology with an average sequencing depth of 25. The details of the SNP calling pipelines and filtering criteria can be found in the original article. After excluding several individuals based on further criteria described in Cagan et al. [92], the final data set in our analysis includes nine *Pan troglodytes* and nine *Homo sapiens*. We used a strict filtering strategy as described by Cagan et al. [92] to avoid artifact bias in analyzing genomic data. Genome segments with tandem repeats, segmental duplication, genomic gaps, and structural variants were excluded according to UCSC tracks [93]. We only analyzed the autosomal regions. When estimating global model parameters in the first step of parameter inference, we excluded CpG islands to reduce the shared polymorphic sites that are recurrent mutations from identical by state processes rather than identical by descent processes [75]. We also excluded gene regions and their upstream and downstream flanking regions of 10 kb to minimize the effect of selection on the estimation of global parameters. The genomic locations of CpG islands were downloaded from the UCSC genome browser, and the gene regions were obtained from UCSC refGene.

### Genomic data analysis

In the first step of parameter estimation, we excluded gene coding regions and the 10-kb flanking regions (upstream and downstream) to minimize the bias caused by natural selection. In the second step of inferring local parameters, we divided the genome into sliding windows with a window size of 10 kb and a step size of 1 kb. We excluded windows with a remaining length < 2 kb after quality filtering; windows with ${S}_{1}+{S}_{2}+{S}_{12}+D<50$ were excluded from the analysis due to limited information. A total of 2,416,717 windows (84.10% of the 2,873,545 total windows) with a mean length of 8856 bp were kept for further analysis.

We corrected the skewness of the distributions of ${\lambda }_{1}$ and ${\lambda }_{2}$ using the Box-Cox method (Additional file 1: Figs. S2 and S3). The corrected distributions were converted to a standard normal distribution. Windows with a normalized $\lambda$ value <  − 3.2905 were identified as under positive selection, and windows with a normalized $\lambda$ value > 3.2905 were identified as under balancing selection.

### Supplementary Information

**Additional file 1: 1.** Forward simulations. **2.** Box-Cox transformation of *λ*. **3.** Likelihood ratio test. **4.** The accuracy of the estimation of *λ *[96]. **5.** The performance of CEGA on detecting balancing selection. **6.** Robustness to different demographic models. **7.** Selection signals detected by LRT. **8.** Genes under selection.

**Additional file 2: Table S1.** The List of Genes under Human-specific Positive selection. **Table S2.** Pathway Enrichment Analysis of Genes under Human-specific Positive Selection. **Table S3.** Disease Enrichment Analysis of Genes under Human-specific Positive Selection. **Table S4.** GO Enrichment Analysis of Genes under Human-specific Positive Selection. **Table S5.** The List of Genes under Human-specific Positive selection in the Non-coding Regions. **Table S6.** Pathway Enrichment Analysis of Genes under Human-specific Positive Selection in the Non-coding Regions. **Table S7.** Disease Enrichment Analysis of Genes under Human-specific Positive Selection in the Non-coding Regions. **Table S8.** GO Enrichment Analysis of Genes under Human-specific Positive Selection in the Non-coding Regions. **Table S9.** The List of Genes under Chimpanzee-specific Positive selection. **Table S10.** Pathway Enrichment Analysis of Genes under Chimpanzee-specific Positive Selection. **Table S11.** Disease Enrichment Analysis of Genes under Chimpanzee-specific Positive Selection. **Table S12.** GO Enrichment Analysis of Genes under Chimpanzee-specific Positive Selection. **Table S13.** The List of Genes under Balancing selection in both the Human and Chimpanzee Lineage. **Table S14.** Pathway Enrichment Analysis of Genes under Balancing Selection in both the Human and Chimpanzee Lineage. **Table S15.** Disease Enrichment Analysis of Genes under Balancing Selection in both the Human and Chimpanzee Lineage. **Table S16.** GO Enrichment Analysis of Genes under Balancing Selection in both the Human and Chimpanzee Lineage. **Table S17.** The List of Genes under Balancing selection in both the Human Lineage. **Table S18.** Pathway Enrichment Analysis of Genes under Balancing Selection in the Human Lineage. **Table S19.** Disease Enrichment Analysis of Genes under Balancing Selection in the Human Lineage. **Table S20.** GO Enrichment Analysis of Genes under Balancing Selection in the Human Lineage.

**Additional file 3.** Peer review history

## Data Availability

The genomic sequences of humans and chimpanzees are downloaded from http://biologiaevolutiva.org/greatape/ [28]. The source code of CEGA is available on GitHub: http://github.com/ChenHuaLab/CEGA [94] and Zenodo: 10.5281/zenodo.8360249 [95]. Both repositories are released under the GPL-2.0 license.
