# Supplementary material for: CEGA: a method for inferring natural selection by comparative population genomic analysis across species
Source: Genome Biol. 2023 Oct 3;24:219. doi: 10.1186/s13059-023-03068-8 (PMC10548728; doi:10.1186/s13059-023-03068-8)
Supplement: Supplementary file 1 — Additional file 1: 1. Forward simulations. 2. Box-Cox transformation of λ. 3. Likelihood ratio test. 4. The accuracy of the estimation of λ [96]. 5. The performance of CEGA on detecting balancing selection. 6. Robustness to different demographic models. 7. Selection signals detected by LRT. 8. Genes under selection. [file 13059_2023_3068_MOESM1_ESM.pdf]

Additional file 1: Supplementary Information

Shilei Zhao[a,b,c,1], Lianjiang Chi[a,b,1], Hua Chen[a,b,c,d,*]

[a] Beijing Institute of Genomics, Chinese Academy of Sciences, Beijing 100101, China
[b] China National Center for Bioinformation, Beijing 100101, China
[c] School of Future Technology, College of Life Sciences and Sino-Danish College, University of Chinese Academy of Sciences, Beijing 100049, China
[d] CAS Center for Excellence in Animal Evolution and Genetics, Chinese Academy of Sciences, Kunming 650223, China

1. Forward simulations

The forward simulations are conducted using the software SLiM 3.6 [90]. The SLiM code template of the simulation of neutral segments, positively selected segments, and balancing selected segments are shown in sections 1.1-1.3, respectively.

1.1. Simulation of neutral segments

```
initialize() {//start of the SLiM code template
    //mutation type
    initializeMutationType(”m1”, 0.5, ”f”, 0.0); // neutral
    // genome element
    initializeGenomicElementType(”g1”, c(m1), 1);
    //set recombination rate
    initializeRecombinationRate(1e-8);
    initializeMutationRate(2.5e-8);
    initializeGenomicElement(g1, 0, 99999);
}
1 {
    sim.addSubpop(”p1”, 10000);
}
```

*Corresponding author: chenh@big.ac.cn
[1] These authors contributed equally.

```
// the first 100000 generations are burn in steps.
100000 {
    sim.addSubpopSplit("p2", 10000, p1);
    sim.addSubpopSplit("p3", 10000, p1);
    p1.setSubpopulationSize(0);
}
220000 {//substituted by 120000 for short term divergence
    sim.addSubpopSplit("p4", 20000, p2);
    sim.addSubpopSplit("p5", 10000, p2);
    p2.setSubpopulationSize(0);
}
//selection happened in p5
420000 late() {//substituted by 160000 for short term divergence
    p3.outputMSSample(20,T,"*","simdata_p3",T);
    p4.outputMSSample(20,T,"*","simdata_p4",T);
    p5.outputMSSample(20,T,"*","simdata_p5",T);
} //end of the SLiM code template
```

The simulated neutral segments are with a length of 100 kb, with the recombination rate 1×10^{-8} and mutation rate 2.5×10^{-8}. All mutations generated in the region are neutral. The first $100,000$ generations are burn-in steps in one simulated population, p1 (the common ancestor of p3, p4, and p5), and the effective population size is 10,000 individuals. At the $100,000$ generation, p1 splits into p2 (effective population size 10,000 individuals) and p3 (effective population size 10,000 individuals). The species p2 is the common ancestor of p4 and p5, and the split time is at the $220,000$ generations. The effective population sizes of p4 and p5 are $20,000$ and $10,000$ individuals, respectively. p3, p4, and p5 are the extant species. At the $420,000$ generations, 20 chromosomes are sampled from each species. The divergence time between p4 and p5 in the above template is $T = 420,000 - 220,000 = 200,000$ generations ago

(backward in time). We also simulate neutral segments under a short divergence time; the generations (bold font) are substituted by 120, 000 and 160, 000, respectively (The corresponding divergence time is $T = 160,000 - 120,000 = 40,000$ generations ago). We repeat the simulation 1,000 times for the scenarios with long-term or short-term divergence time.

1.2. simulation of positively selected segments

```
initialize(){//start of the SLiM code template
    // mutation type
    initializeMutationType("m1", 0.5, "f", 0.0); // neutral
    initializeMutationType("m2", 0.5, "f", 0.002);// homozygous selection advantage
    // genome element
    initializeGenomicElementType("g1", c(m1), 1); //
    initializeGenomicElementType("g2", c(m2), 1);
    //set recombination rate and mutation rate
    initializeRecombinationRate(1e-8);
    initializeMutationRate(2.5e-8);
    initializeGenomicElement(g1, 0, 4999);
    initializeGenomicElement(g2, 5000, 5099);
    initializeGenomicElement(g1, 5100, 14999);
    initializeGenomicElement(g2, 15000, 15099);
    initializeGenomicElement(g1, 15100, 24999);
    initializeGenomicElement(g2, 25000, 25099);
    initializeGenomicElement(g1, 25100, 34999);
    initializeGenomicElement(g2, 35000, 35099);
    initializeGenomicElement(g1, 35100, 44999);
    initializeGenomicElement(g2, 45000, 45099);
    initializeGenomicElement(g1, 45100, 54999);
    initializeGenomicElement(g2, 55000, 55099);
```

```
    initializeGenomicElement(g1, 55100, 64999);
    initializeGenomicElement(g2, 65000, 65099);
    initializeGenomicElement(g1, 65100, 74999);
    initializeGenomicElement(g2, 75000, 75099);
    initializeGenomicElement(g1, 75100, 84999);
    initializeGenomicElement(g2, 85000, 85099);
    initializeGenomicElement(g1, 85100, 94999);
    initializeGenomicElement(g2, 95000, 95099);
    initializeGenomicElement(g1, 95100, 99999);
}
1 {
    sim.addSubpop("p1", 10000); }
    // the first 100000 generations are burn in steps.
100000 {
    sim.addSubpopSplit("p2", 10000, p1);
    sim.addSubpopSplit("p3", 10000, p1);
    p1.setSubpopulationSize(0);
}
220000 {//substituted by 120000 for short term divergence
    sim.addSubpopSplit("p4", 20000, p2);
    sim.addSubpopSplit("p5", 10000, p2);
    p2.setSubpopulationSize(0);
}
//selection happened in p5
fitness(m2,p1) {
    return 1.0;
}//
fitness(m2,p2) {
    return 1.0;
```

```
}//
fitness(m2,p3) {
    return 1.0;
}//
```

fitness(m2,p4) {

return 1.0;

}//

```
420000 late() {//substituted by 160000 for short term divergence
    p3.outputMSSample(20,T,"*","simdata_p3",T);
    p4.outputMSSample(20,T,"*","simdata_p4",T);// Chimpanzee
    p5.outputMSSample(20,T,"*","simdata_p5",T);// Human
} //end of the SLiM code template
```

We also simulate scenarios with long and short divergence times for positively-selected segments. We set homozygous selection advantage to 0.001, 0.002, 0.004, 0.01, 0.02, and the heterozygous effect $h = 0.5$, which means the heterozygote selection advantages are $s = 0.0005$, 0.001, 0.002, 0.005, 0.01. The segments have a length of 100 kb, with 99 kb under neutrality and 1 kb under positive selection. The positively selected regions scatter in ten areas, corresponding to the different cis-regulating elements. Positive selection occurs in species p5. We had ten groups of positively selected segments corresponding to different selection intensities and divergence times, each with 200 repetitions.

1.3. Segments under balancing selection

```
initialize() {//start of the SLiM code template
    //mutation type
    initializeMutationType("m1", 0.5, "f", 0.0);// neutral
    initializeMutationType("m2", 2, "f", 0.002);// under balancing selection, h=2
    // genome element
```

```
    initializeGenomicElementType("g1", m1, 1.0);
    //set recombination rate
    initializeRecombinationRate(1e-8);
    initializeMutationRate(2.5e-8);
    initializeGenomicElement(g1, 0, 9999);
}
// Create the ancestral African population
1 {
// save this run's identifier, used to save and restore
    defineConstant("simID", getSeed());
    sim.addSubpop("p1", 10000);
}
// the first 100000 generations are burn in steps.
100000 late() {
    sim.addSubpopSplit("p2", 10000, p1);
    sim.addSubpopSplit("p3", 10000, p1);
    p1.setSubpopulationSize(0);
}
220000 late() {
    sim.addSubpopSplit("p4", 20000, p2);
    sim.addSubpopSplit("p5", 10000, p2);
    p2.setSubpopulationSize(0);
}
260000 late() {
    // save the state of the simulation
    sim.outputFull("filePath/slim_" + simID + ".txt");
    // introduce the sweep mutation
    target = sample(p5.genomes, 1);
    target.addNewDrawnMutation(m2, 4999);
```

```
}
260000:420000 late() {
    if (sim.countOfMutationsOfType(m2) == 0)
{
    // go back to generation 10000
    sim.readFromPopulationFile("filePath/slim_" + simID + ".txt");
    // start a newly seeded run
    setSeed(rdunif(1, 0, asInteger(2 ∧ 62) - 1));
    // re-introduce the sweep mutation
    target = sample(p5.genomes, 1);
    target.addNewDrawnMutation(m2, 4999);
    }
}
420000 late() {
    p3.outputMSSample(20,T,"*","simdata_p3",T);
    p4.outputMSSample(20,T,"*","simdata_p4",T);
    p5.outputMSSample(20,T,"*","simdata_p5",T);
    //sample size of n genomes, not n individuals
} //end of the SLiM code template
```

For balancing selection, we simulate segments of length 10 kb. We introduce the allele under balancing selection at the center of the segment with the different onset times of 80,000, 160,000 (occurred in the p5 lineage), 240,000, and 280,000 (occurred in the p2 lineage) generations ago (backward from sampling time). The heterozygous effect is set to $h = 2$. If the selected allele dies out, the simulation process is restarted when the allele under balancing selection is first introduced. We set different selection intensities of 0.001, 0.002, and 0.004. We simulate 12 groups of balancing selected segments corresponding to various selection onset times and selection intensities, each with 200 repetitions.

2. Box-Cox transformation of $\boldsymbol{\lambda}$

The original distributions of λ_h and λ_c are skewed (Fig. S1). We correct the skewness with the Box-Cox transformation method. The normalized transformed λ fits well with the standard normal distribution (cyan curves in Fig. S2).

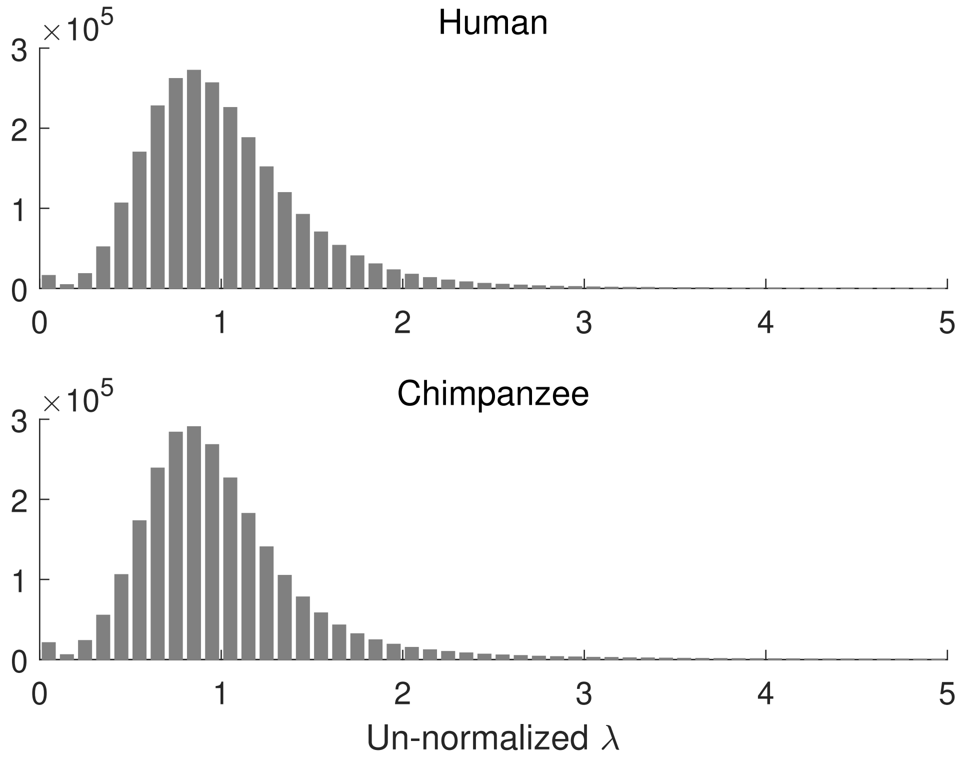

Fig. S1. The distributions of un-normalized λ

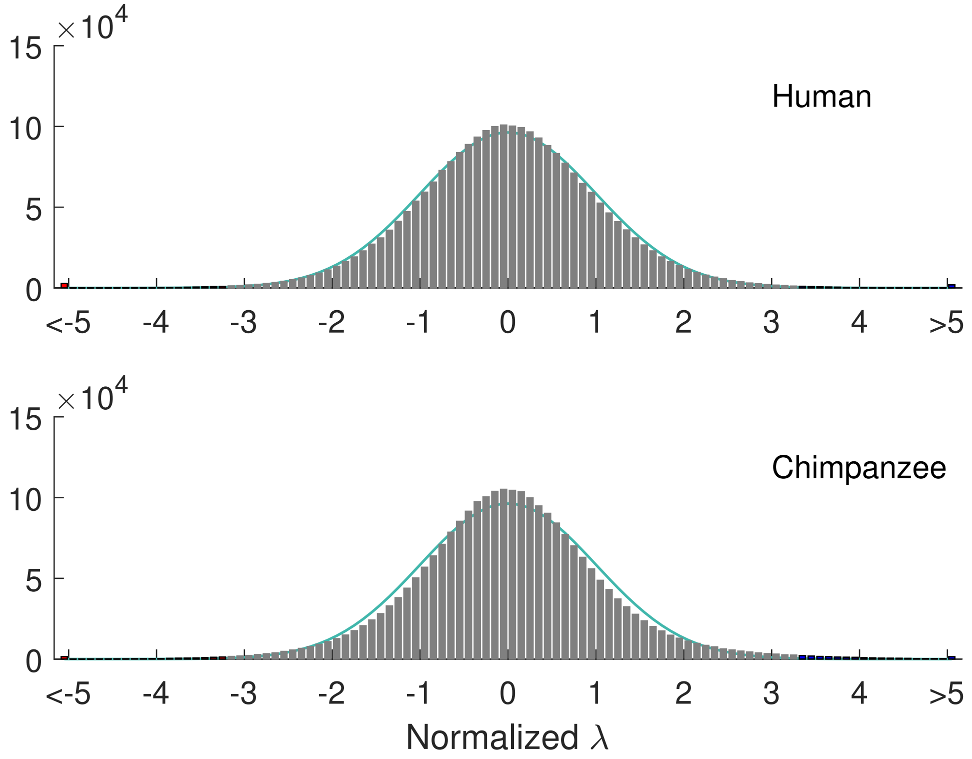

Fig. S2. The distributions of normalized λ after the Box-Cox transformation. The cyan curves are the standard normal distributions.

3. Likelihood ratio test

The null hypothesis for the likelihood ratio test assumes fixed values of λ_1^l and λ_2^l, both set to 1, and a free variable of the local mutation rate μ^l. The alternative hypothesis 1 assumes λ_1^l a free variable; a fixed value of $\lambda_2^l = 1$; and a free variable of the local mutation rate μ^l. The alternative hypothesis 2 assumes λ_2^l a free variable; $\lambda_1^l = 1$; and the local mutation rate μ^l a free variable as well. Denote the likelihood under the null hypothesis as $L(\theta_0)$ and under the alternative hypothesis 1 as $L(\theta_1)$. We found that $2LLR = 2[ln(L(\theta_1)) - ln(L(\theta_0))]$ does not follow the Chi-squared distribution with 1 degree of freedom $\chi^2(1)$. However, $2LLR$ follows the $\chi^2(1)$ distribution after adjustment with an inflation factor

$$k = \frac{ICDF_{\chi^2(1)}(0.5)}{M_e(2LLR)}, \tag{S1}$$

where $ICDF_{\chi^2(1)}(0.5)$ denotes the inverse cumulative distribution function of $\chi^2(1)$ at 0.5, and $M_e(2LLR)$ denotes the median of $2LLR$.

Fig. S3 shows the probability density function of $\chi^2(1)$ (red line) and the distribution of adjusted $2LLR$ from the analysis results of the Human and Chimpanzee genomic data.

We then compared the power of CEGA based on the empirical distribution of λ (CEGA-λ, after Box-Cox transformation, λ_1 and λ_2 follow a normal distribution) and the likelihood ratio test (CEGA-LRT). We applied CEGA to the simulated data sets as described in the main text. As shown in Fig. S4, CEGA-LRT seems slightly more conservative than CEGA-λ. The mean power of CEGA-λ, CEGA-LRT, and MLHKA for data simulated with the divergence time of 200,000 generations are 0.7408, 0.6160, and 0.5010, respectively. For the data simulated with the divergence time of 40,000 generations, the mean power of CEGA-λ, CEGA-LRT, and MLHKA are 0.7692, 0.5520, and 0.4483, respectively.

For balancing selection, we also compared the two methods using simulated data as described in the main text. CEGA-λ also performs better than CEGA-LRT (Fig. S5).

In summary, CEGA-λ outperforms CEGA-LRT in detecting positive selection and balancing selection. However, we still provide in the implementation of CEGA the option -LRT for users to conduct a likelihood ratio test.

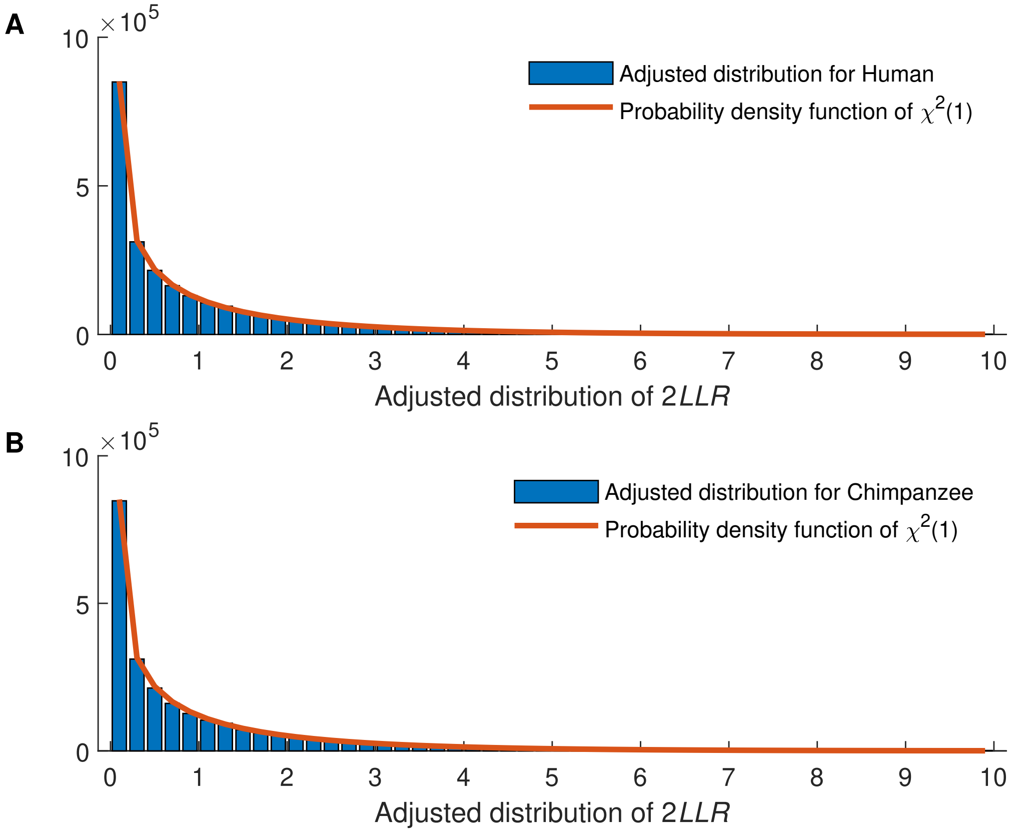

Fig. S3. Distributions of $2LLR$ after adjustment. **A** The distribution of adjusted $2LLR$ from genomic data of Human. **B** The distribution of adjusted $2LLR$ from genomic data of Chimpanzee.

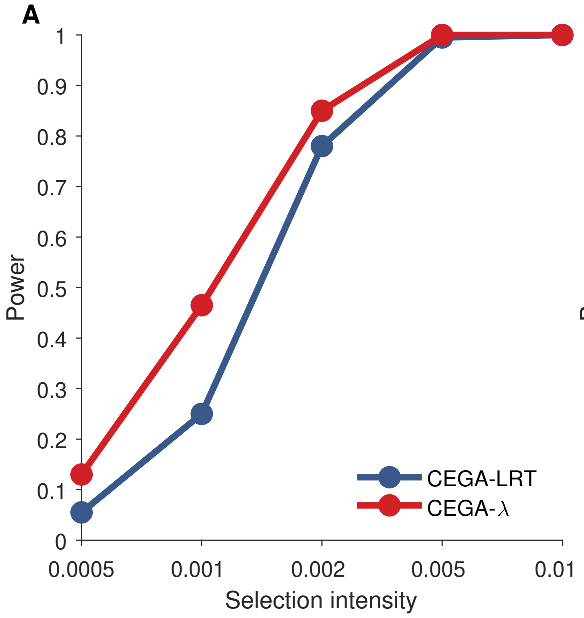

Fig. S4. Comparison of power to detect positive selection using the likelihood ratio test (blue line) and the adjusted λ empirical distribution (red line). **A** and **B** are for data simulated with a divergence time of 200,000 and 40,000 generations respectively.

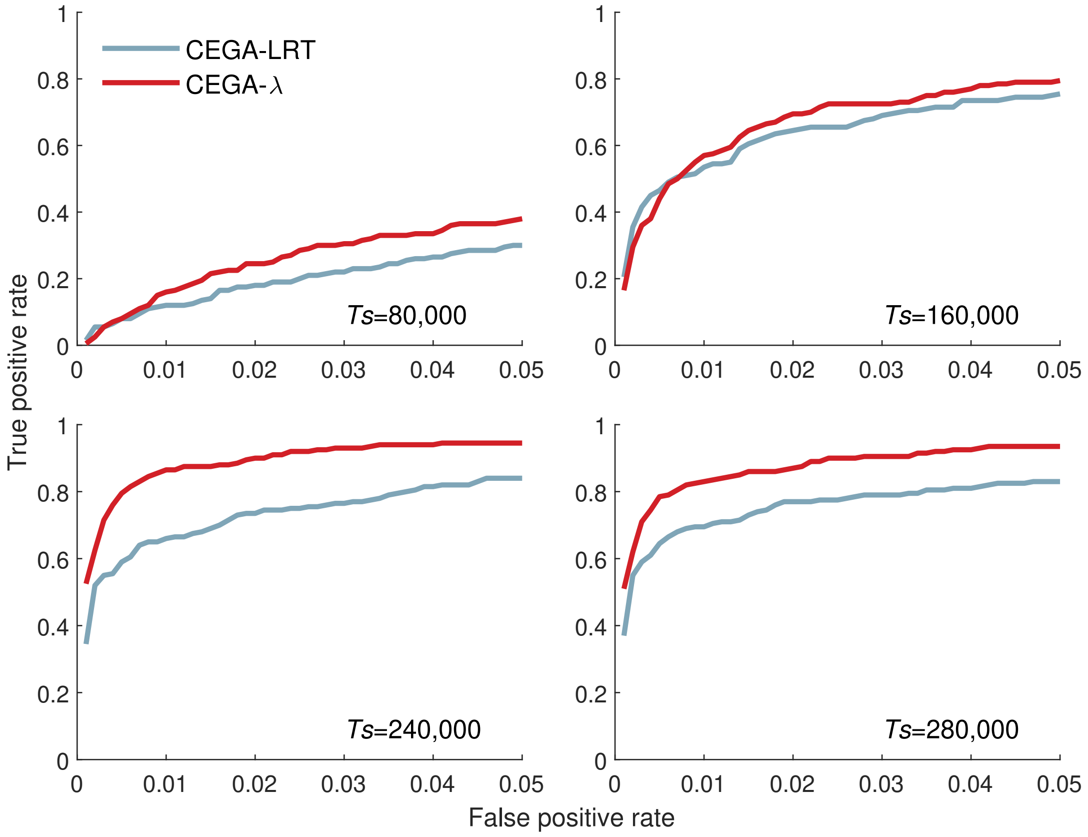

Fig. S5. Comparison of performance of detecting balancing selection using likelihood ratio test (blue line) and the adjusted λ empirical distribution (red line).

4. The accuracy of the estimation of $\boldsymbol{\lambda}$

In the forward simulations, we did not set values of λ as a parameter. Instead, we set the values of selection intensity. To directly test the accuracy of the estimates of λ, we carried out a new set of simulations using the coalescent simulator MSMS [96]. For a target population under selection, we set its effective population size to be $\lambda \times N_e$, with λ =0.1, 0.3, 0.5, 0.7, 0.9, 1.5, 2.0, and 3.0. The values of $\lambda < 1$ correspond to positive selection scenarios, and the values of $\lambda > 1$ correspond to balancing selection scenarios. As shown in Fig. S6, the estimations of λ are unbiased.

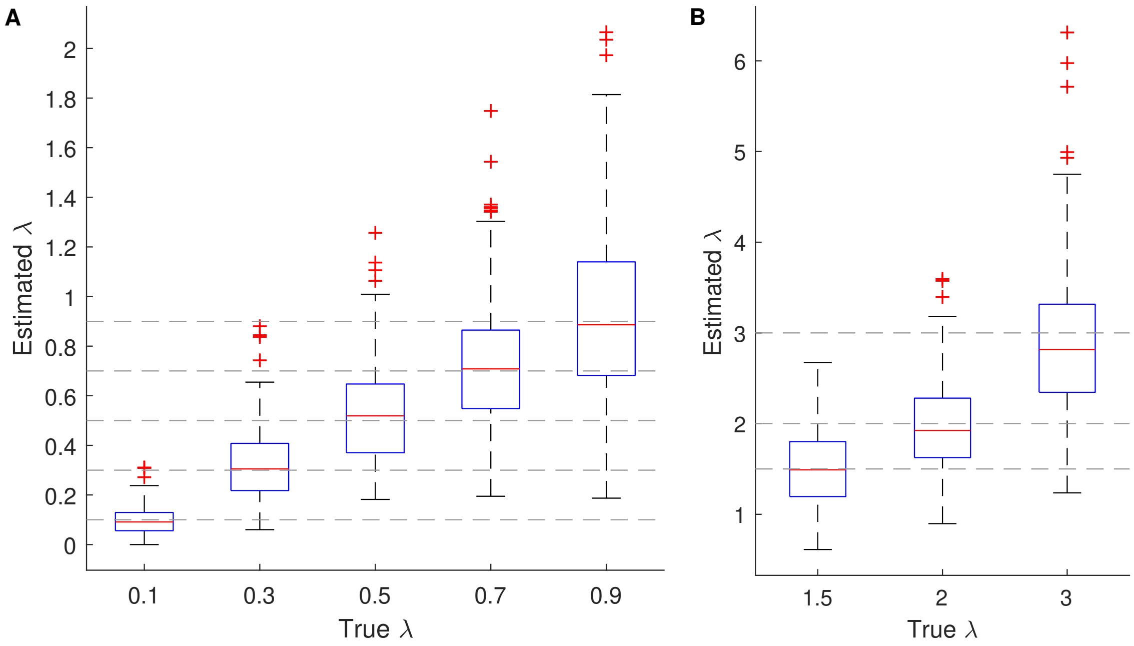

Fig. S6. Performance of CEGA on the estimation of λ. **A** $\lambda < 1$ (positive selection) **B** $\lambda > 1$ (balancing selection).

5. The performance of CEGA on detecting balancing selection

We test the performance of CEGA on detecting balancing selection under different selection onset times, window sizes, and selection intensities. The mean power of detecting balancing selection under different window sizes of 0.5kb, 1kb, 2kb, 4kb, 6kb, and 10kb are 0.2988, 0.4669, 0.5256, 0.4631, 0.4169, and 0.3294 respectively (Fig. S7 **A**). CEGA has the best performance for detecting balancing selection when the window size is around 2kb. CEGA has higher power for detecting long-term balancing selection than recent selection. The power of identifying balancing selection also increases with the selection intensity (Fig. S7 **B**).

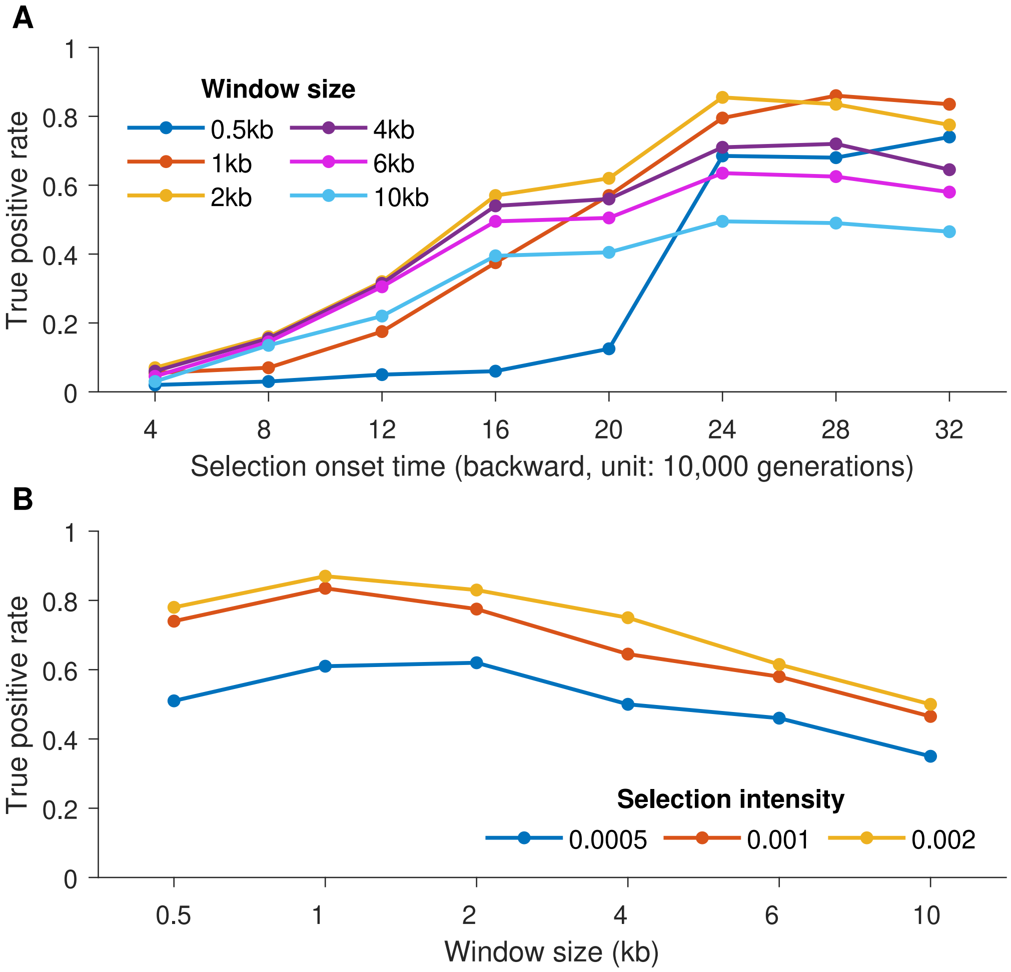

Fig. S7. Performance of CEGA on detecting balancing selection signals. **A** The power of CEGA to detect balancing selection signals under different selection onset times and window sizes. The selection intensity was set to $s = 0.001$. **B** The power of CEGA to detect balancing selection signals with different window sizes and selection intensities. The selection onset time was set to 320,000 generations before the sampling date.

6. Robustness to different demographic models

We tested the robustness of CEGA to different demographic models. For comparison, we first simulate data under a constant population model with $Ne = 10,000$ (Constant model), and the other parameter settings are the same as the simulations

with the long-term divergence time in former sections. Five non-equilibrium demographic models are further considered with the population size changes in Population 1 (parameters of the models can be set using the SLiM code template shown in Section 1.1): (1) Ancient severe bottleneck (A-S-BN): occurred 30,600-30,000 generations ago, with an effective population size of 0.1*Ne during the bottleneck stage; (2) Ancient mild bottleneck (A-M-BN): occurred 30,200-30,000 generations ago, with an effective population size of 0.1*Ne; (3) Recent severe bottleneck (R-S-BN): occurred 4,000-3,400 generations ago, with an effective population size of 0.1*Ne during the bottleneck stage; (4) Recent mild bottleneck (R-M-BN): occurred 4,000-3,800 generations ago, with an effective population size of 0.1*Ne during the bottleneck stage; and (5) Exponential growth (EG): The population started growth 923 generations ago, with a growth rate of 0.5% per generation, and the effective population size increased from 10,000 to 1,000,000.

As expected, ancient bottlenecks only slightly affect the observed polymorphic sites compared with the constant demographic model. For R-S-BN, R-M-BN, A-S-BN, and A-M-BN, the polymorphic sites are reduced to 79.86%, 91.25%, 96.07%, 98.58% of that of constant demographic model with $Ne = 10,000$. For the EG, the polymorphic sites increased to 108.62% of the constant model (Fig. S8 **A**). All demographic models have a slight effect on divergence sites (Fig. S8 **B**). We then applied CEGA to the simulated data by assuming constant population sizes. For R-S-BN, R-M-BN, A-S-BN, A-M-BN, and EG, the inferred population sizes are 78.46%, 90.43%, 95.19%, 98.34%, and 109.25% of $10,000$ (Fig. S9). We presented the number of polymorphism sites in Population 1 together with the theoretical expectation predicted by CEGA model with the approximated constant population sizes (Eqn. (4)-(7) in the main text) in Fig. S10. The two numbers are very close to each other. The other three summary statistics show similar pattern. The results indicate that the observed four statistics in the bottleneck and exponential growth model are approximately equivalent to the constant population size model with an effective population size estimated by CEGA.

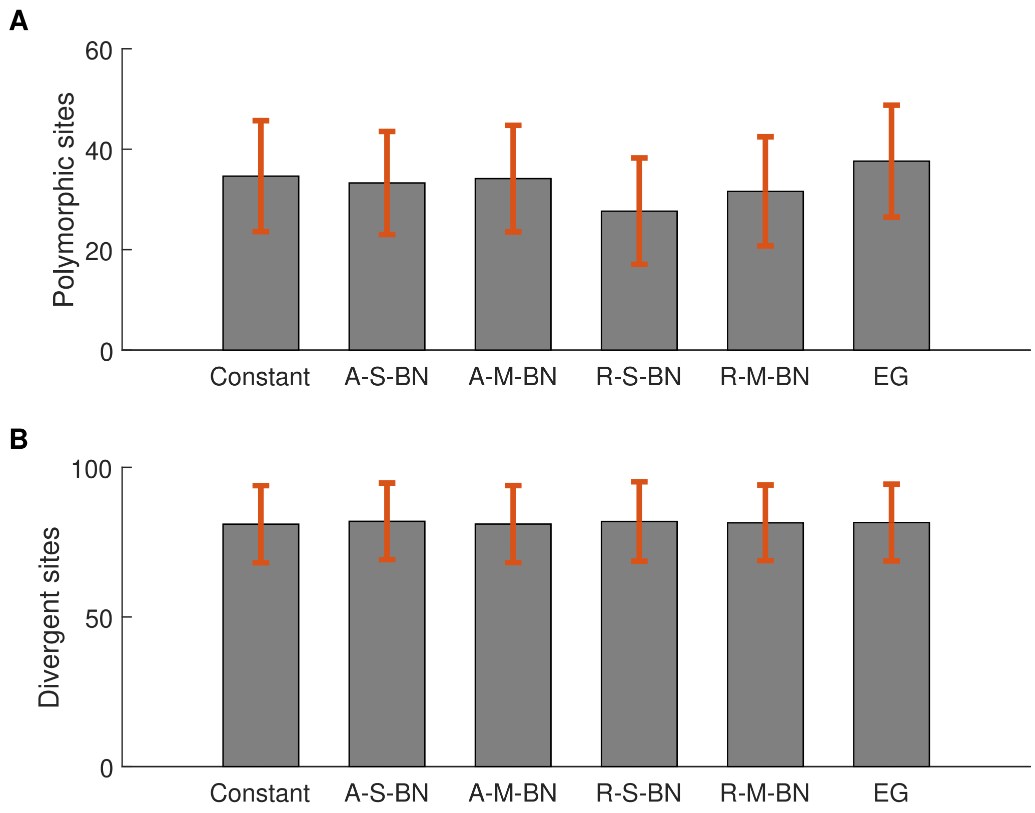

Fig. S8. The observed polymorphic sites **A** and divergence sites **B** in the simulation data sets.

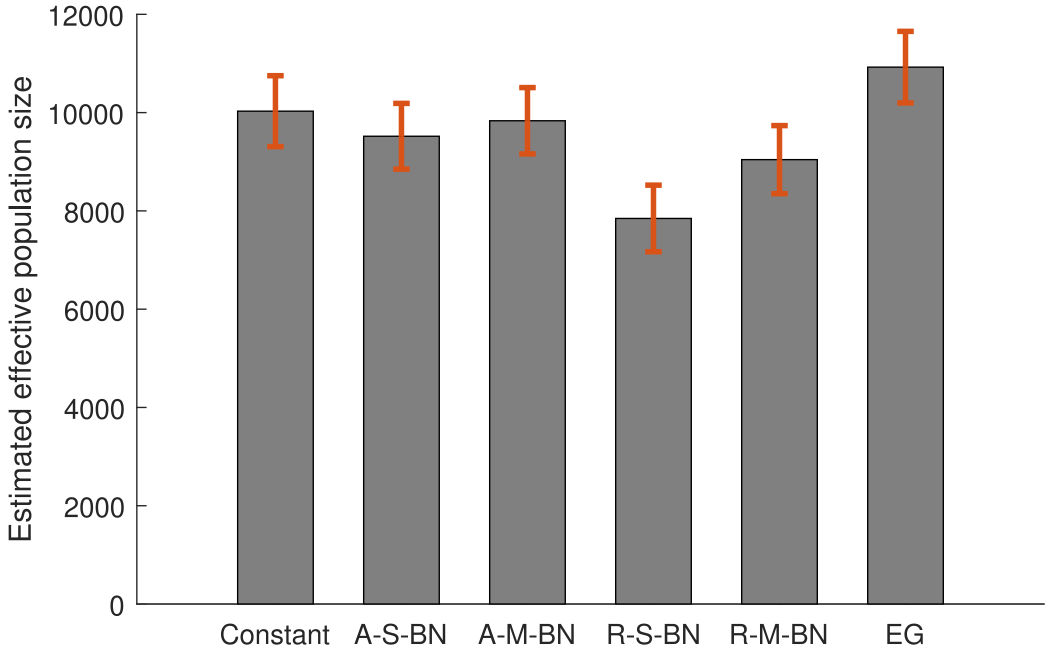

Fig. S9. The estimated effective population size by CEGA for different demographic models.

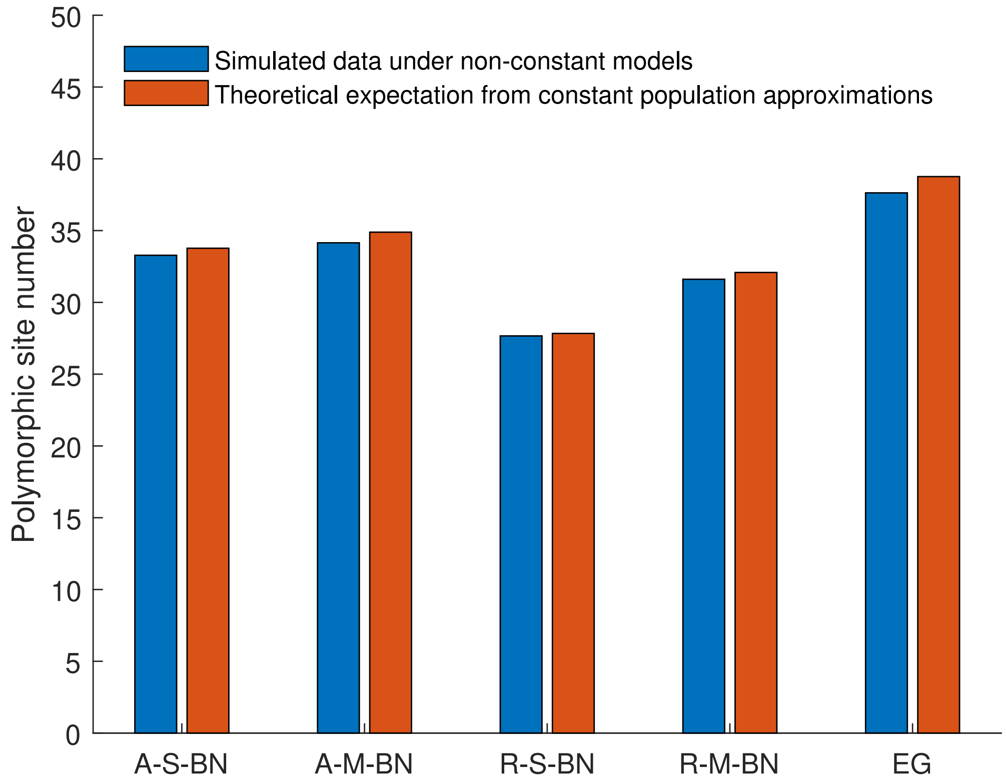

Fig. S10. The comparison of polymorphic site numbers from simulated data under the five non-equilibrium models and the theoretical expectation from approximated population history with constant sizes.

7. Selection signals detected by LRT

We present two methods to evaluate the significance of a test, including the normalized λ (CEGA-λ) and the likelihood ratio test (CEGA-LRT). CEGA-λ demonstrates a bit higher power to detect selection. We then checked the p-values of CEGA-LRT around genes identified by CRGA-λ. The p-values of CEGA-LRT around these genes all show signal patterns similar to CEGA-λ (Figs. S11 and S12).

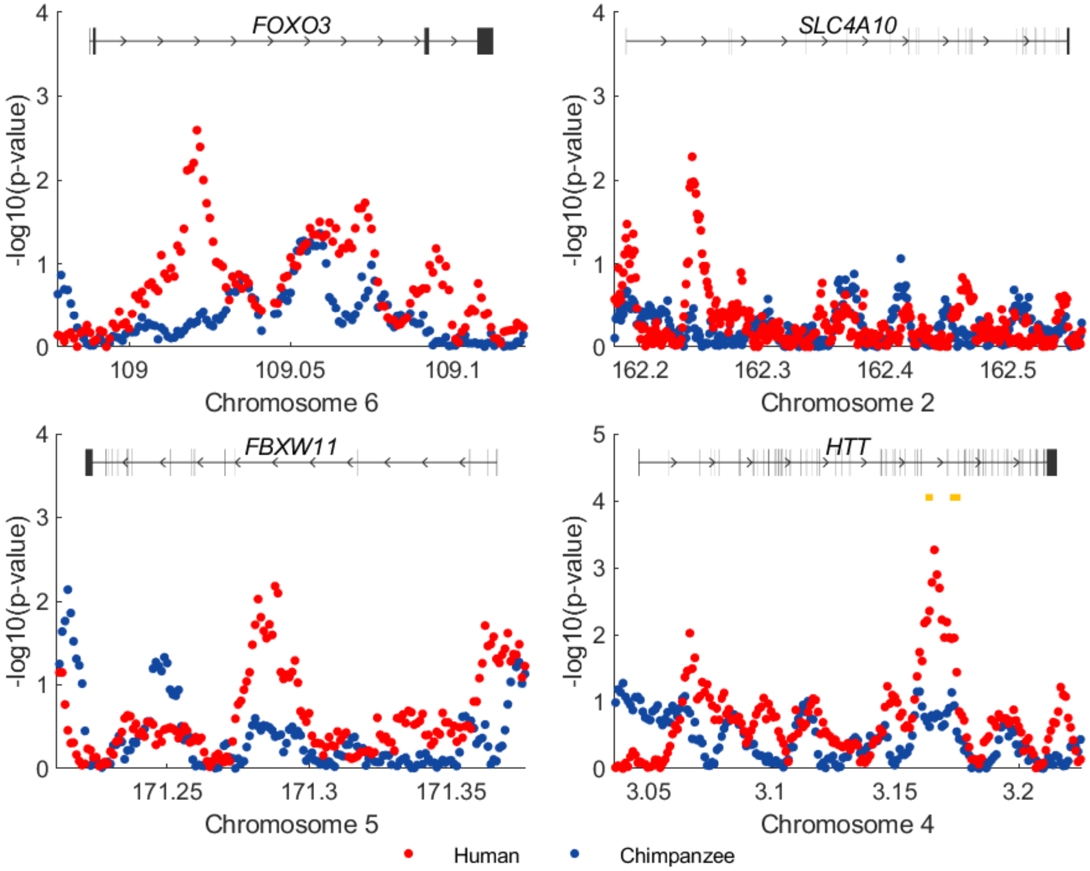

Fig. S11. Four genes of the brain morphogenesis pathway show signals of positive selection only in noncoding regions in the human lineage (detected by CEGA-LRT).

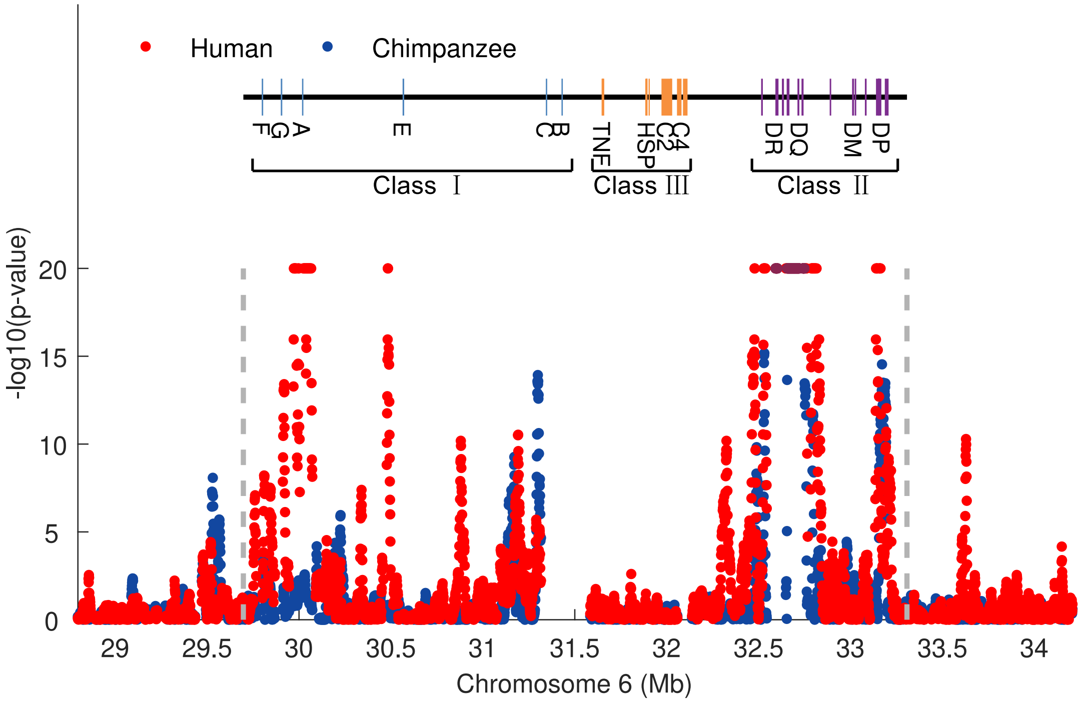

Fig. S12. Balancing selection signals around the MHC region of humans and chimpanzees (detected by CEGA-LRT).

8. Genes under selection

The $-\log p$ values of the normalized λ of a subset of genes identified under positive selection are shown in Figs. S13-S37. A subset of genes identified under balancing selection are shown in Figs. S38-S39.

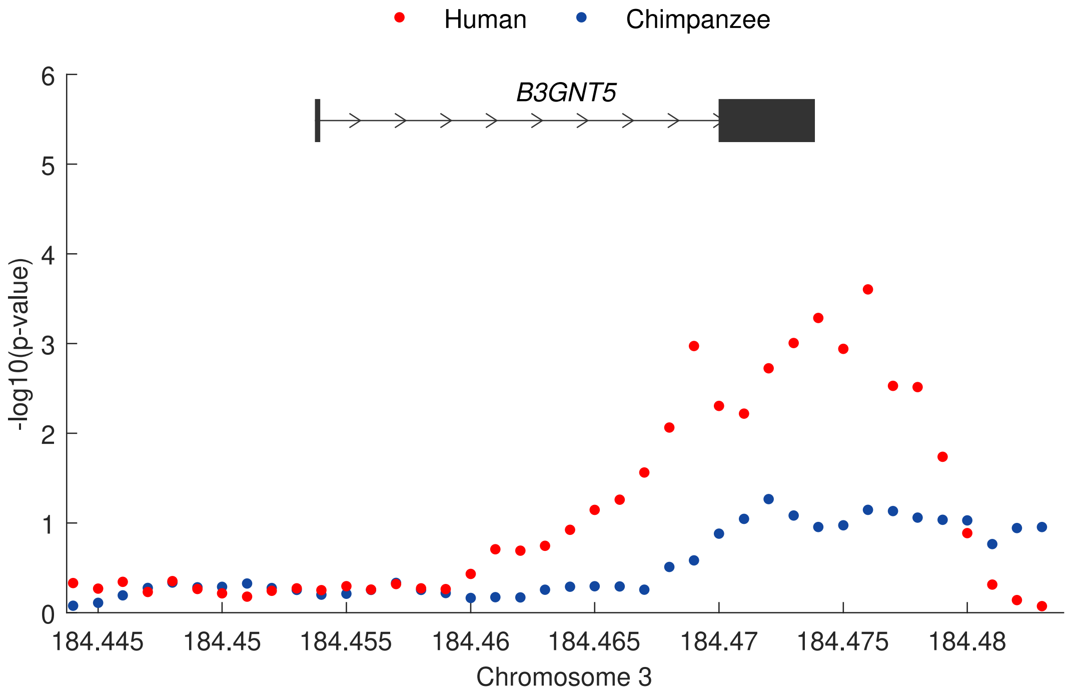

Fig. S13. -log10(p-value) of normalized λ around the positively selected gene *B3GNT5*.

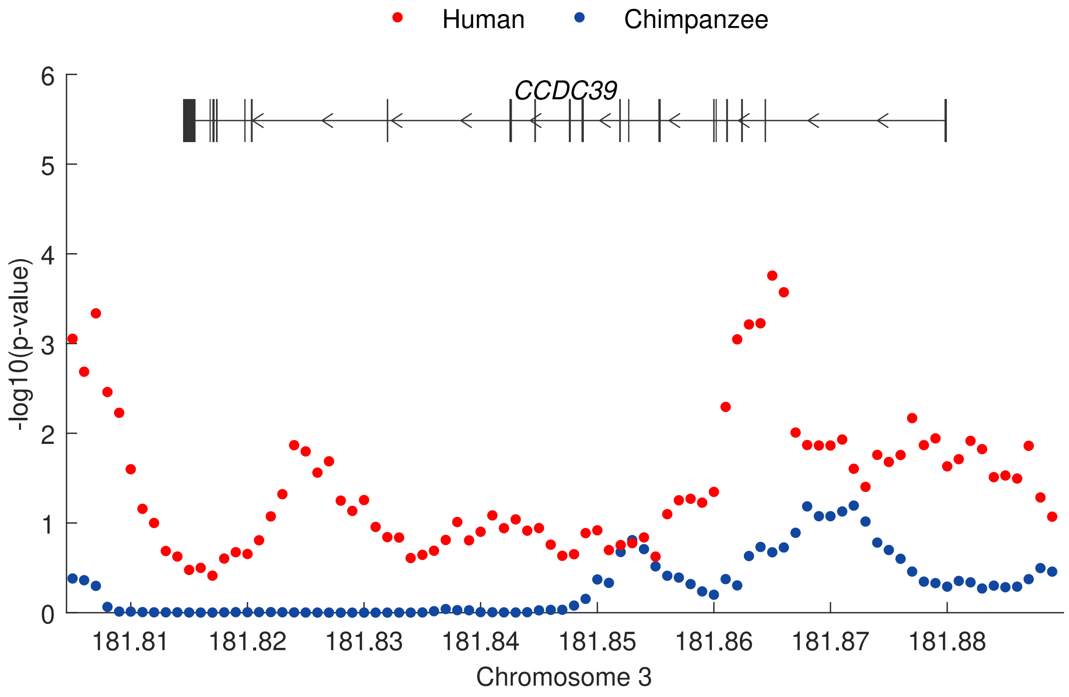

Fig. S14. -log10(p-value) of normalized λ around the positively selected gene *CCDC39*.

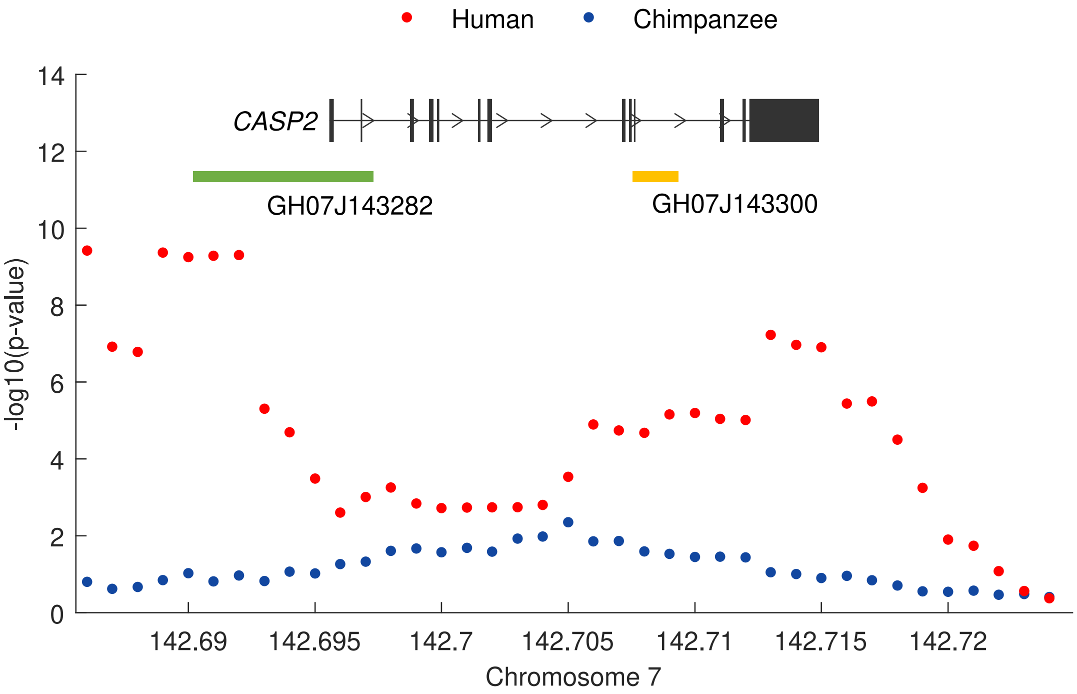

Fig. S15. -log10(p-value) of normalized λ around the positively selected gene *CASP2*.

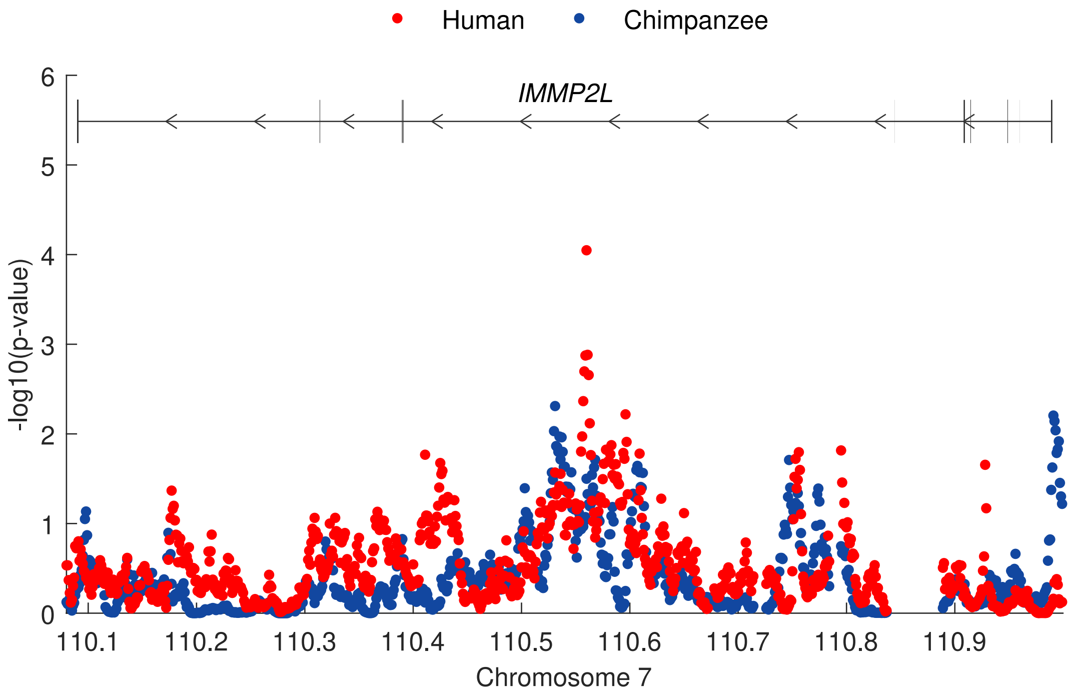

Fig. S16. -log10(p-value) of normalized λ around the positively selected gene *IMMP2L*.

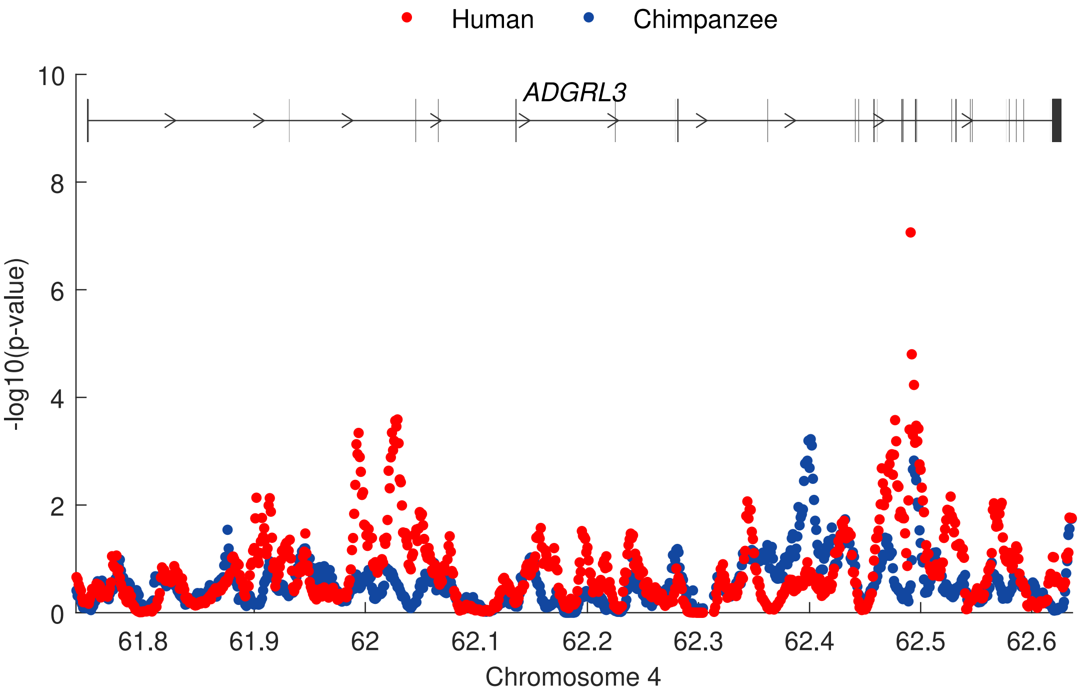

Fig. S17. -log10(p-value) of normalized λ around the positively selected gene *ADGRL3*.

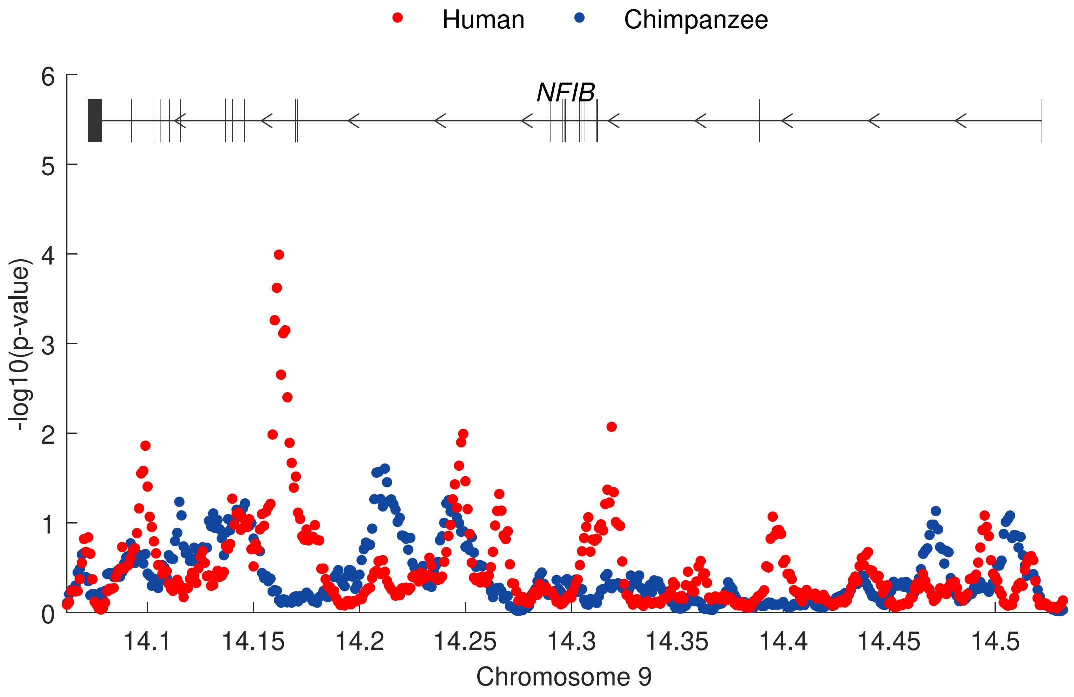

Fig. S18. -log10(p-value) of normalized λ around the positively selected gene *NFIB*.

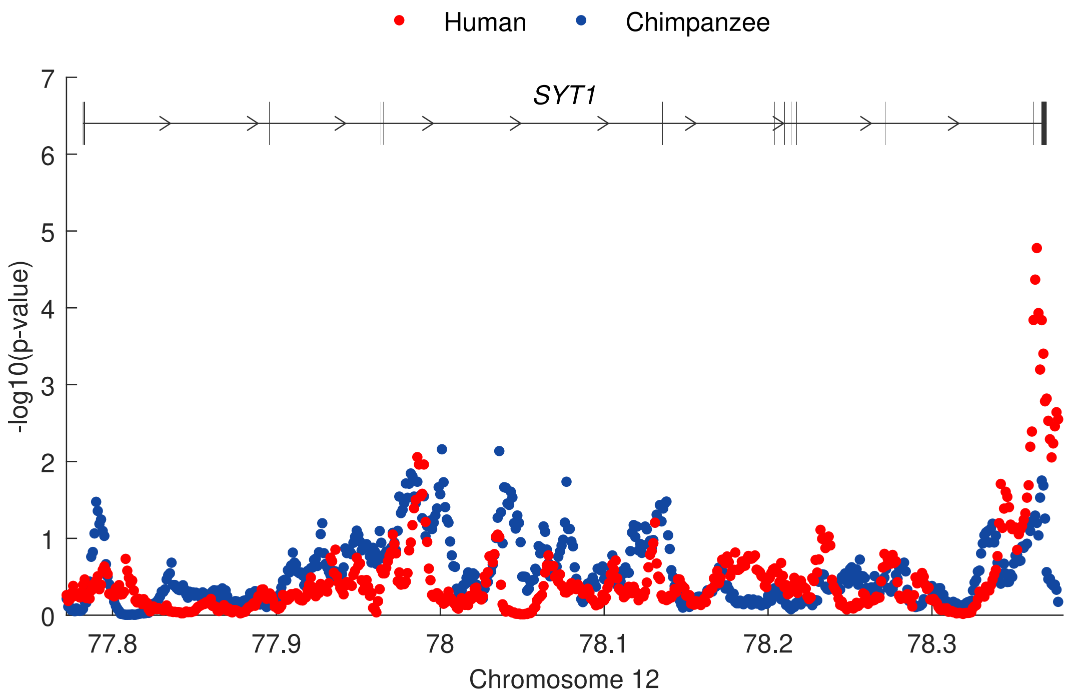

Fig. S19. -log10(p-value) of normalized λ around the positively selected gene *SYT1*.

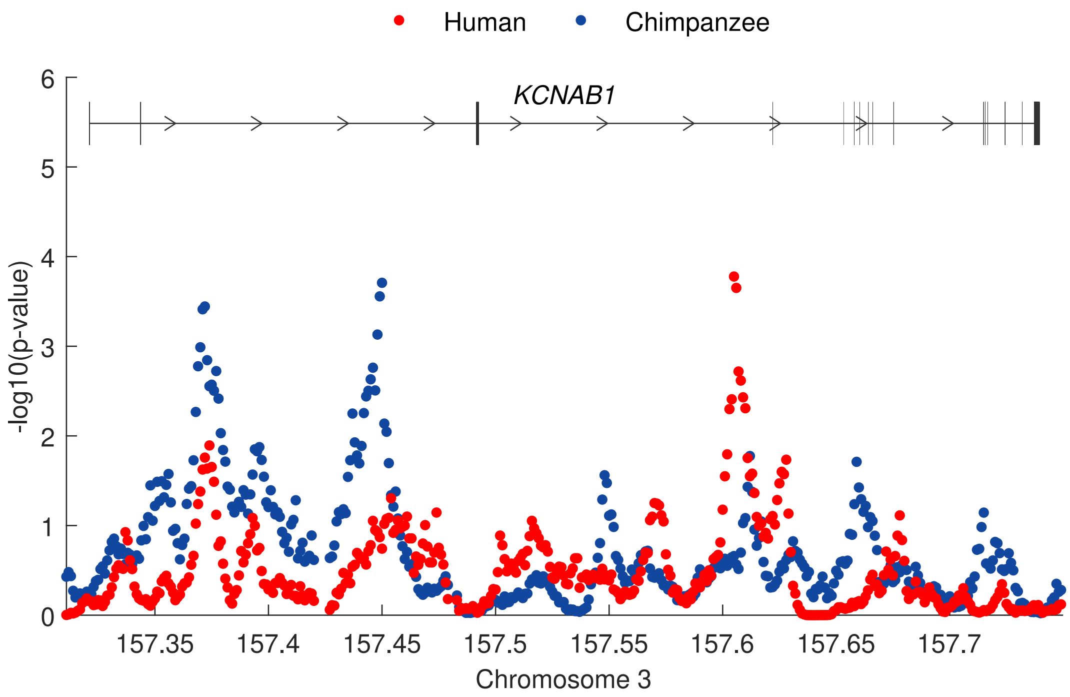

Fig. S20. -log10(p-value) of normalized λ around the positively selected gene *KCNAB1*.

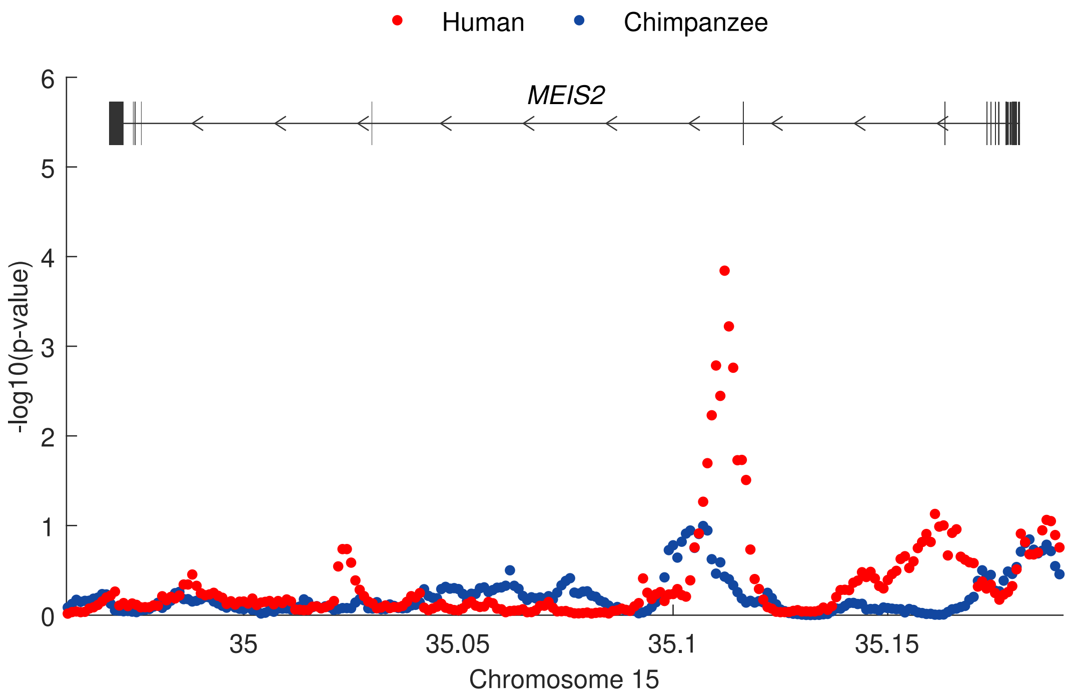

Fig. S21. -log10(p-value) of normalized λ around the positively selected gene *MEIS2*.

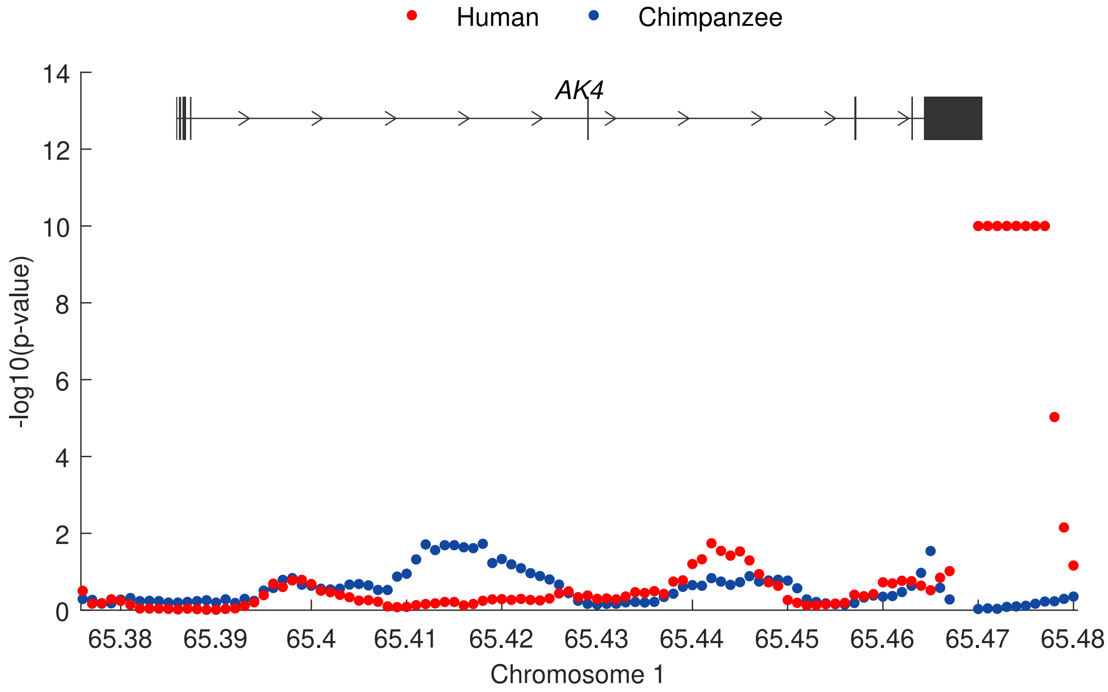

Fig. S22. -log10(p-value) of normalized λ around the positively selected gene *AK4*.

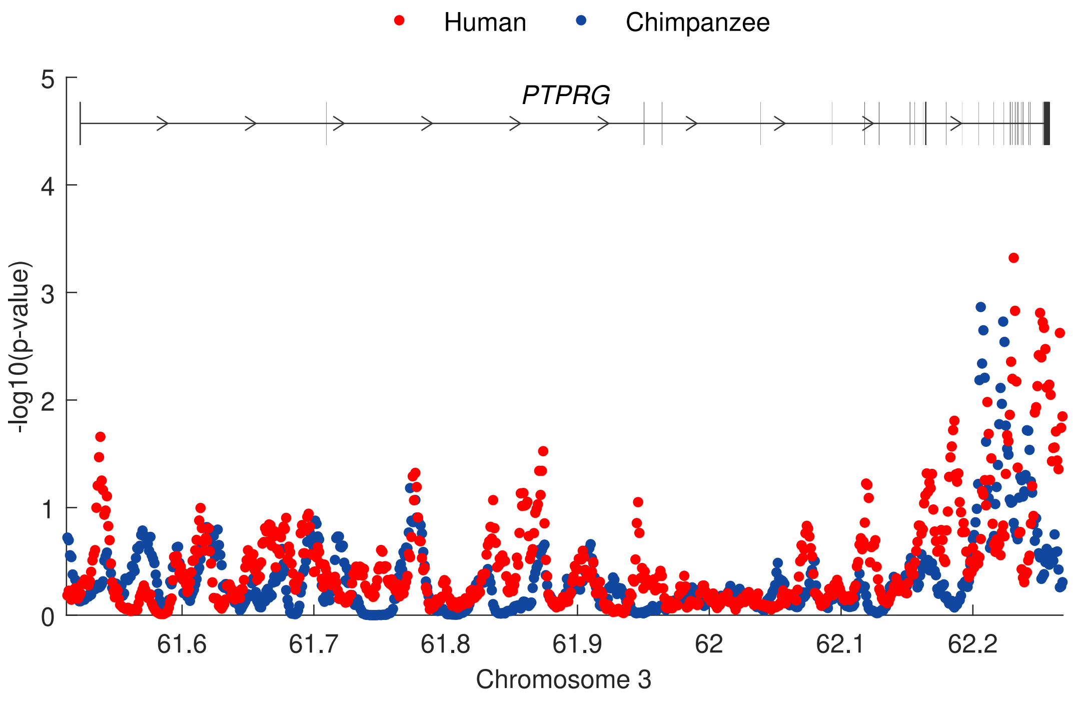

Fig. S23. -log10(p-value) of normalized λ around the positively selected gene *PTPRG*.

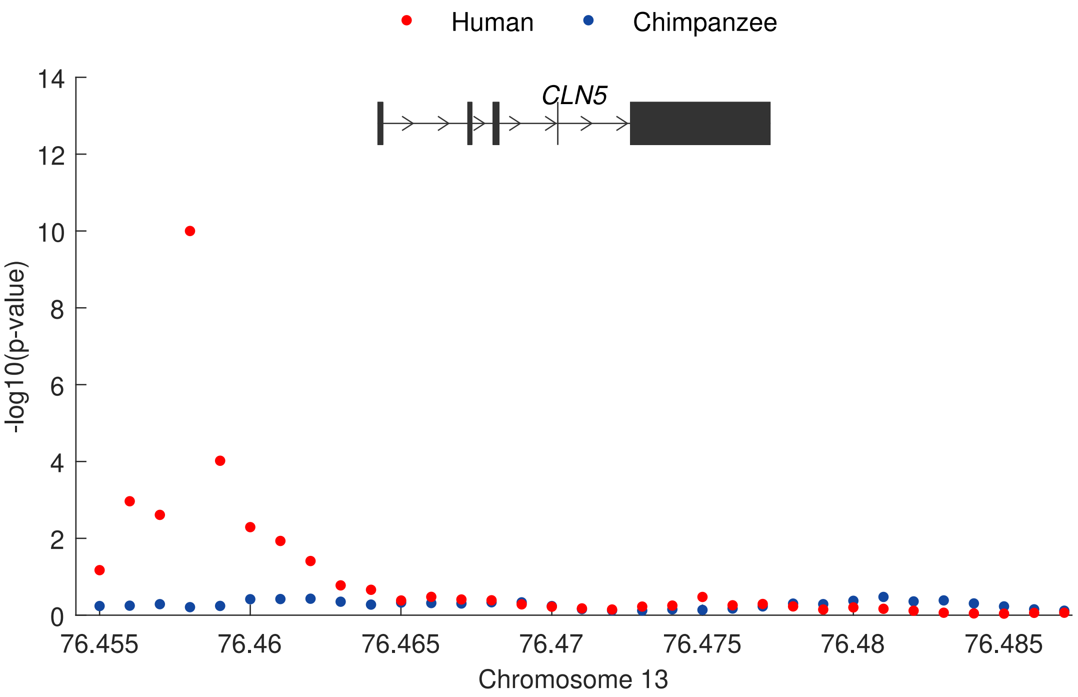

Fig. S24. -log10(p-value) of normalized λ around the positively selected gene *CLN5*.

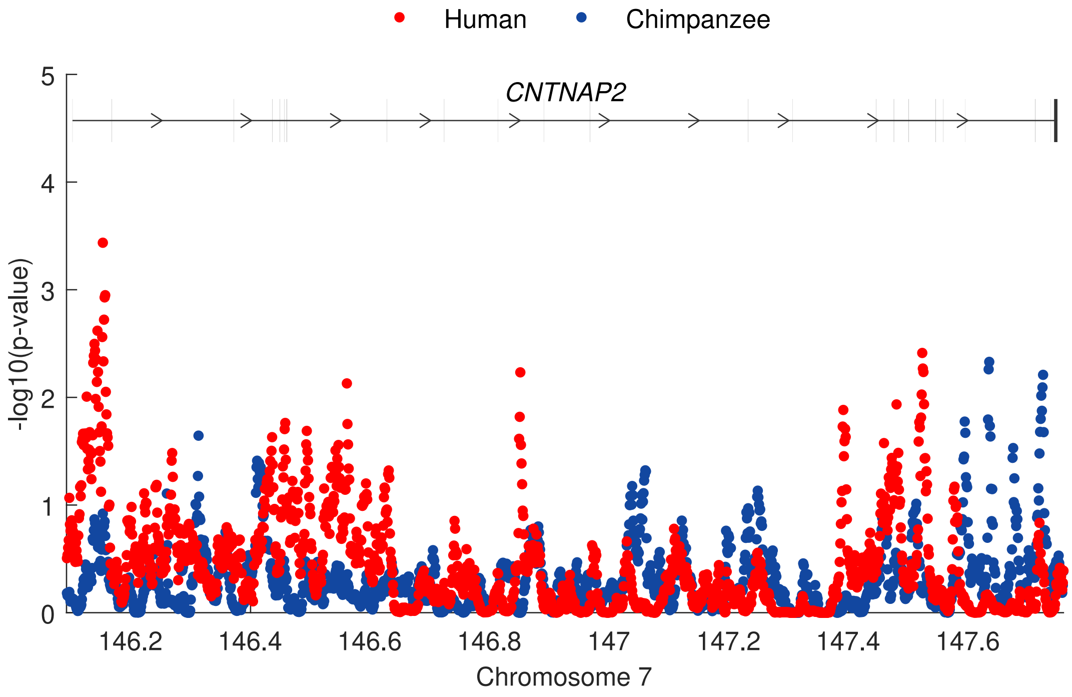

Fig. S25. -log10(p-value) of normalized λ around the positively selected gene *CNTNAP2*.

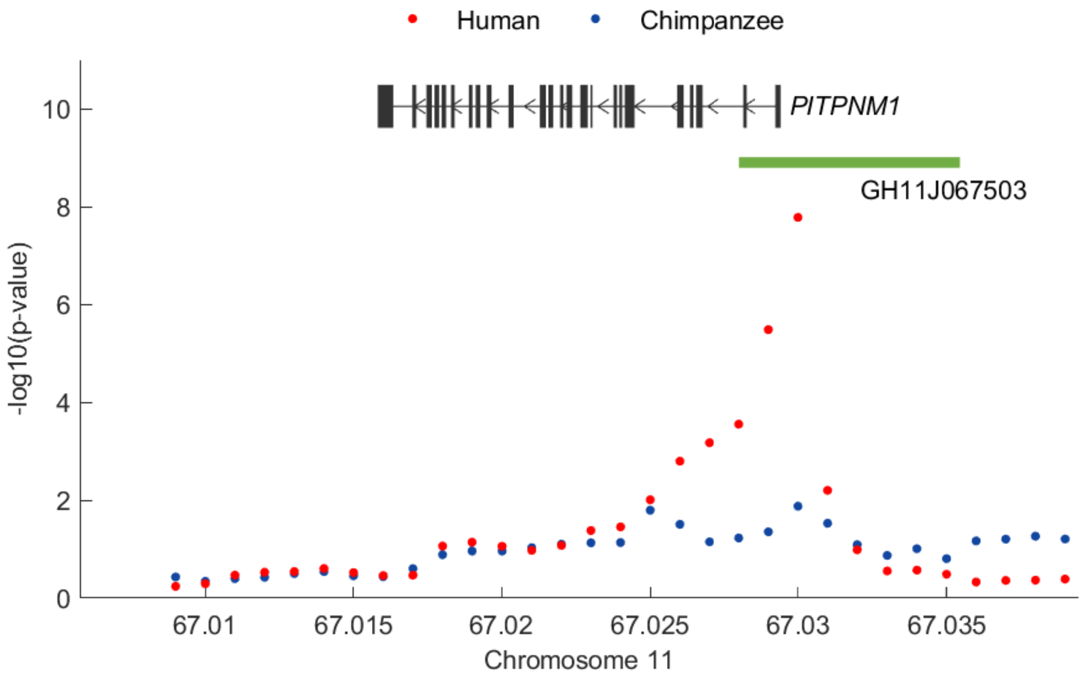

Fig. S26. -log10(p-value) of normalized λ around the positively selected gene *PITPNM1*.

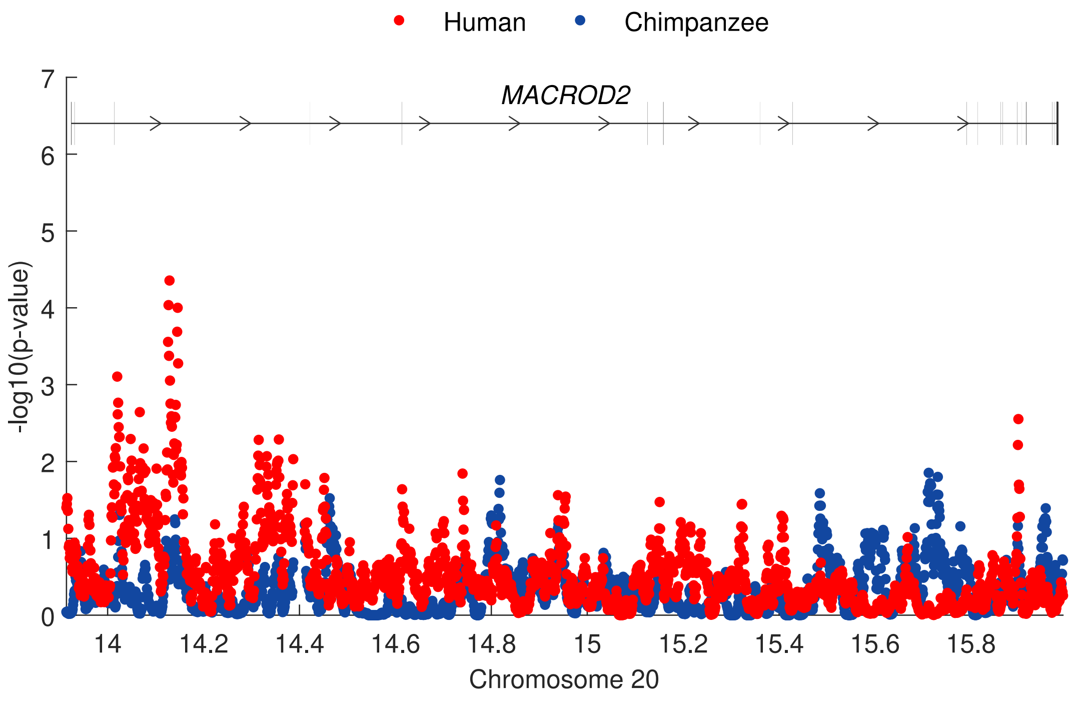

Fig. S27. -log10(p-value) of normalized λ around the positively selected gene *MACROD2*.

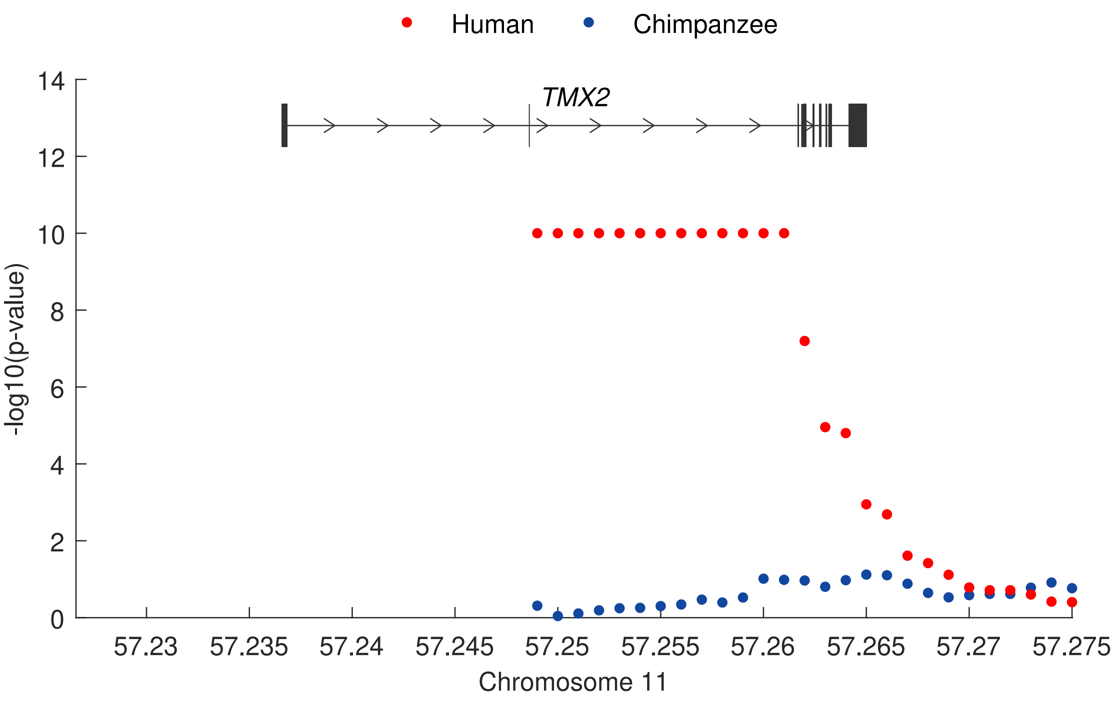

Fig. S28. -log10(p-value) of normalized λ around the positively selected gene *TMX2*.

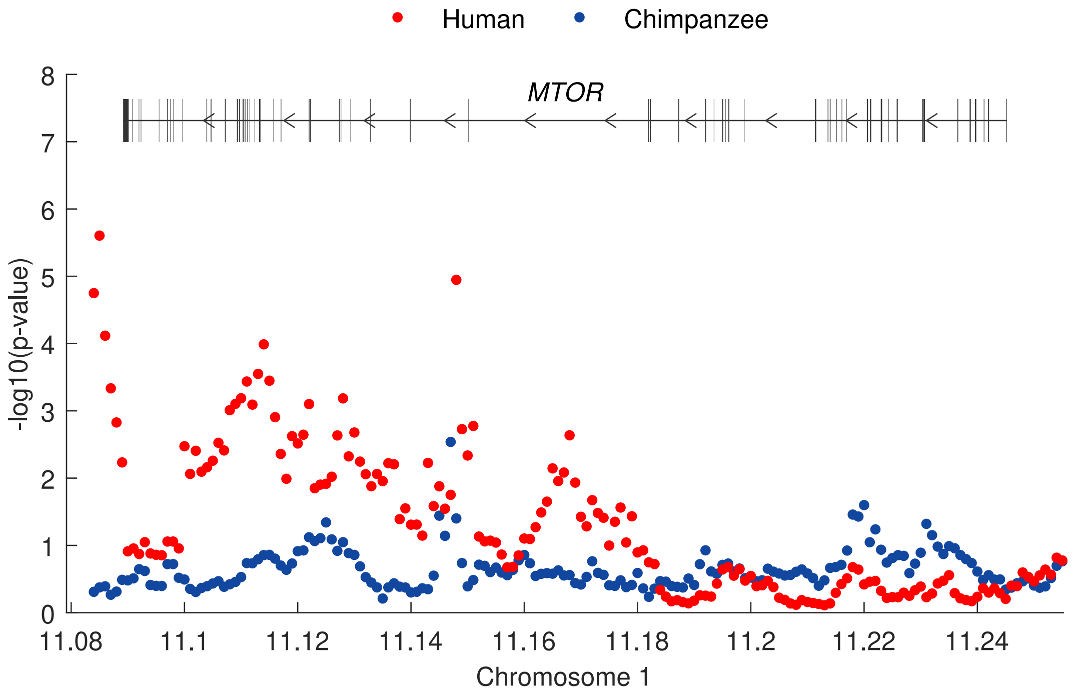

Fig. S29. -log10(p-value) of normalized λ around the positively selected gene *MTOR*.

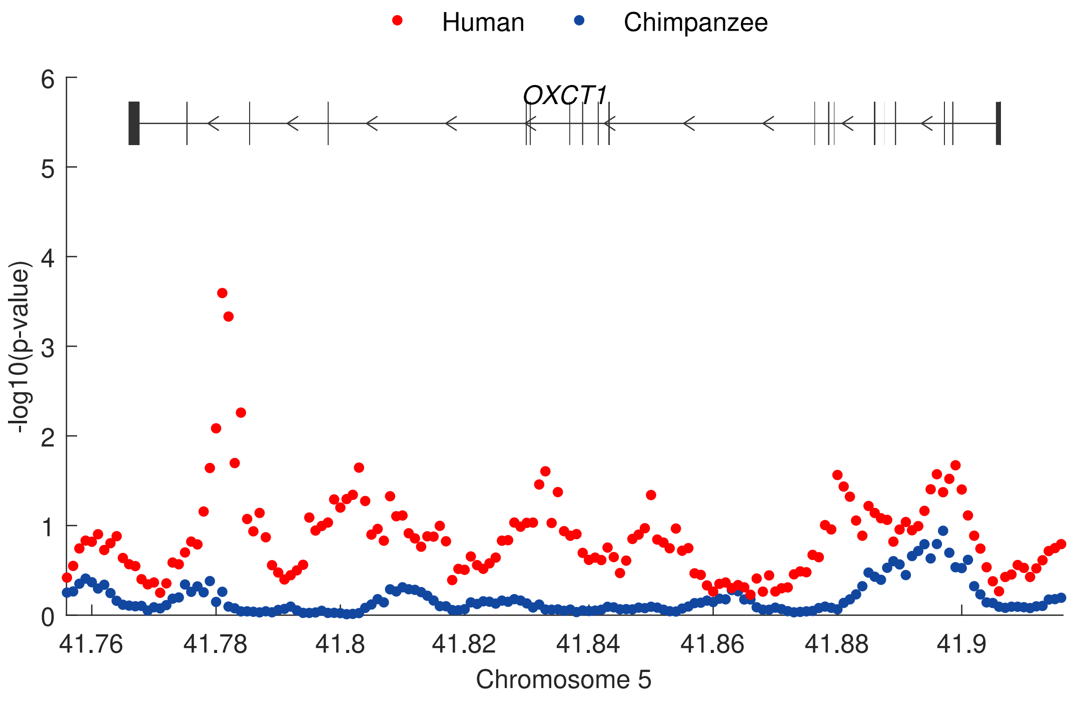

Fig. S30. -log10(p-value) of normalized λ around the positively selected gene *OXCT1*.

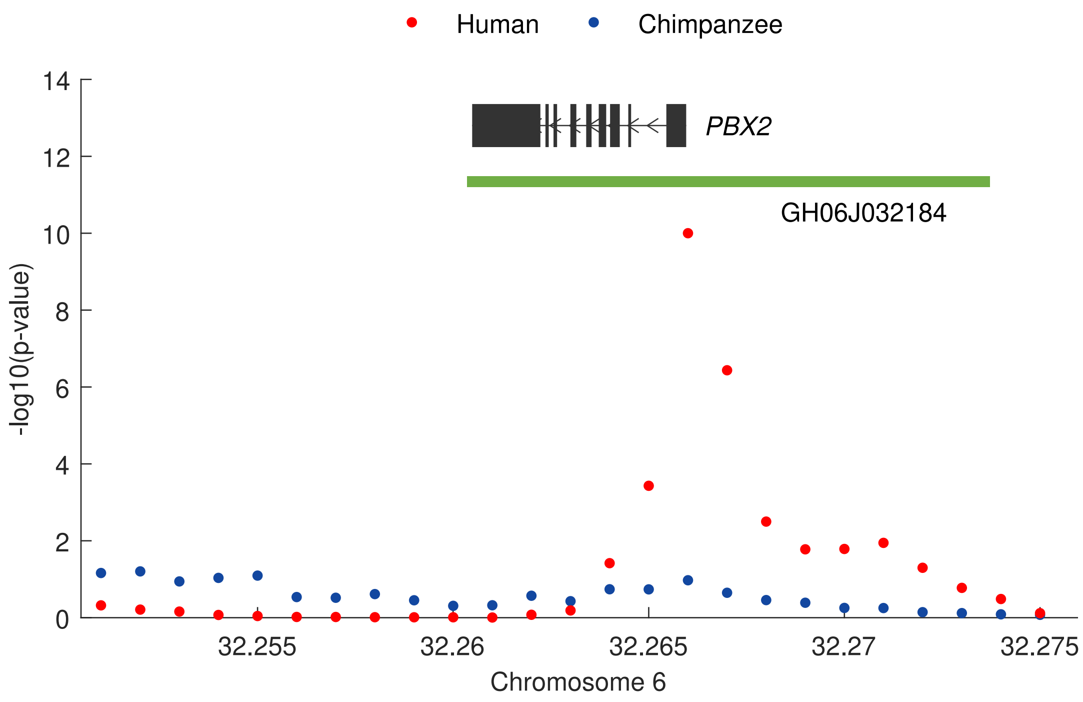

Fig. S31. -log10(p-value) of normalized λ around the positively selected gene *PBX2*.

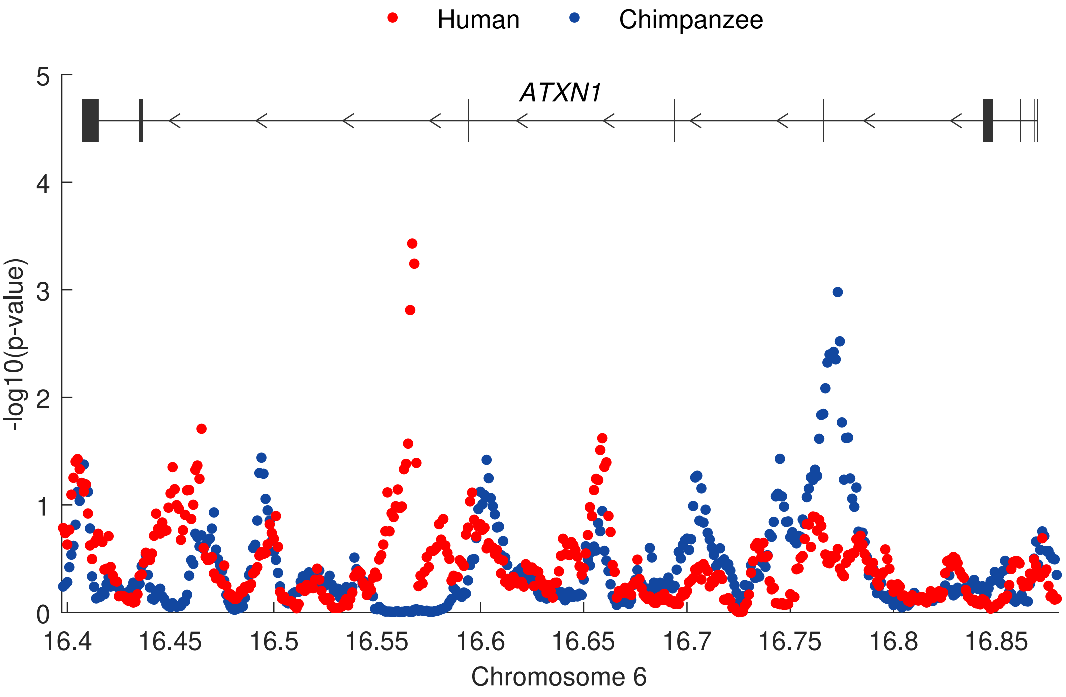

Fig. S32. -log10(p-value) of normalized λ around the positively selected gene *ATXN1*.

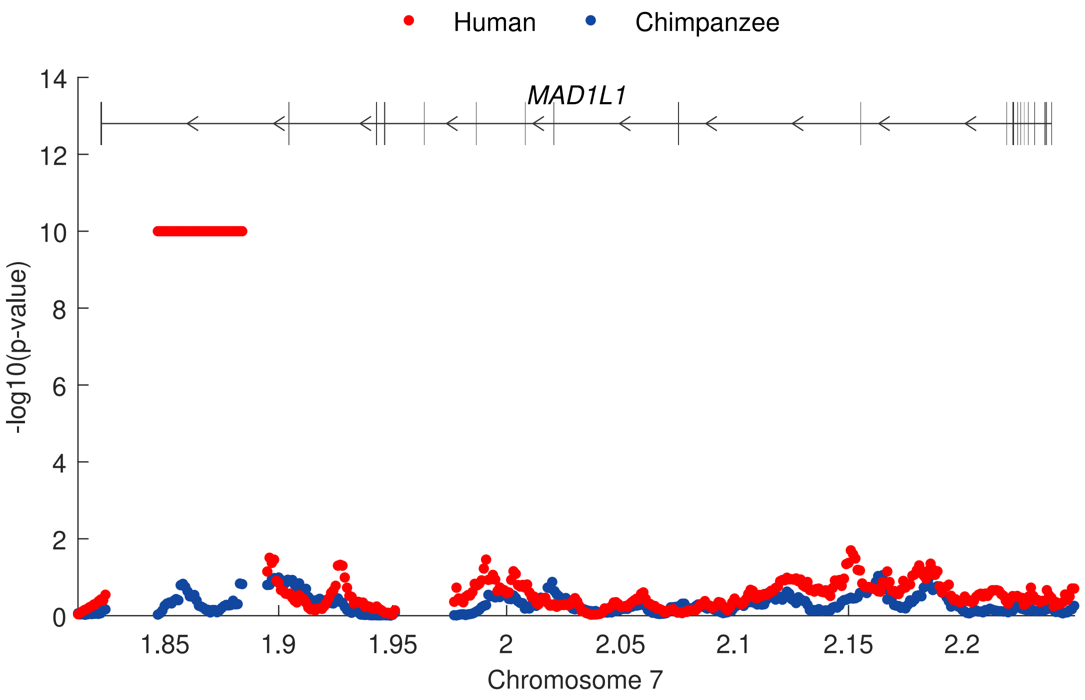

Fig. S33. -log10(p-value) of normalized λ around the positively selected gene *MAD1L1*.

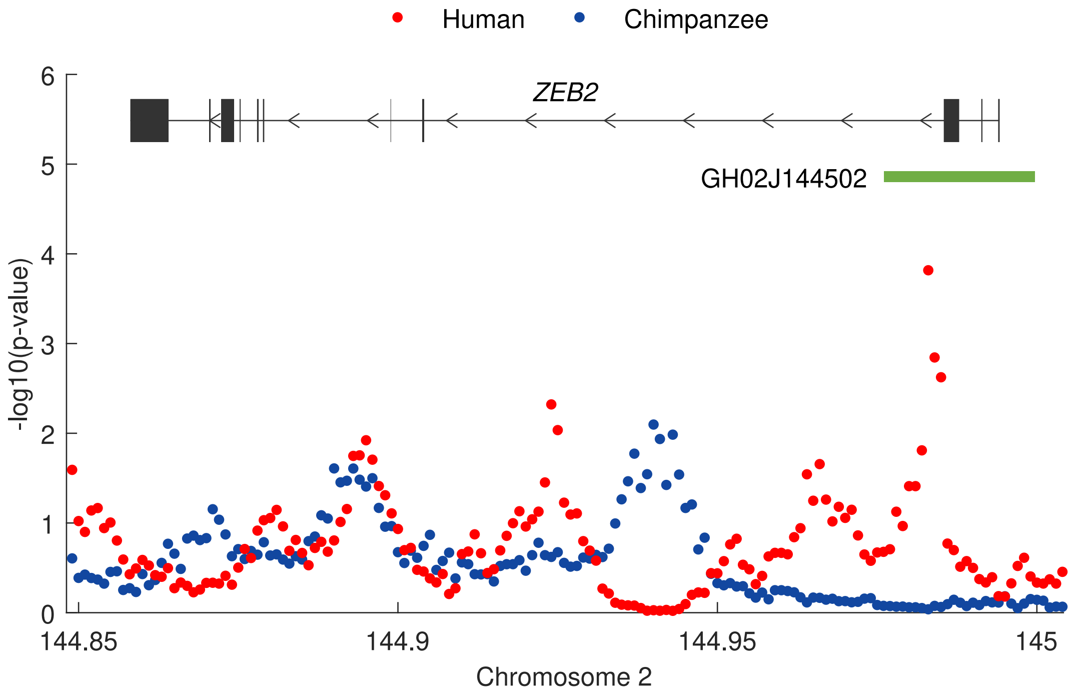

Fig. S34. -log10(p-value) of normalized λ around the positively selected gene *ZEB2*.

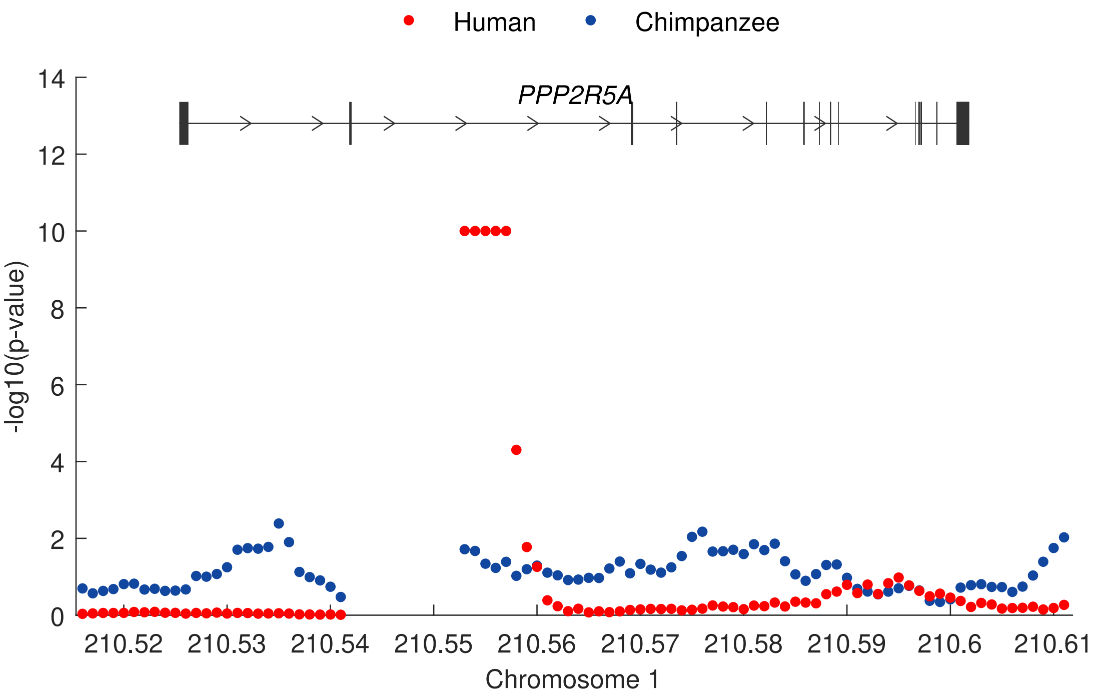

Fig. S35. -log10(p-value) of normalized λ around the positively selected gene *PPP2R5A*.

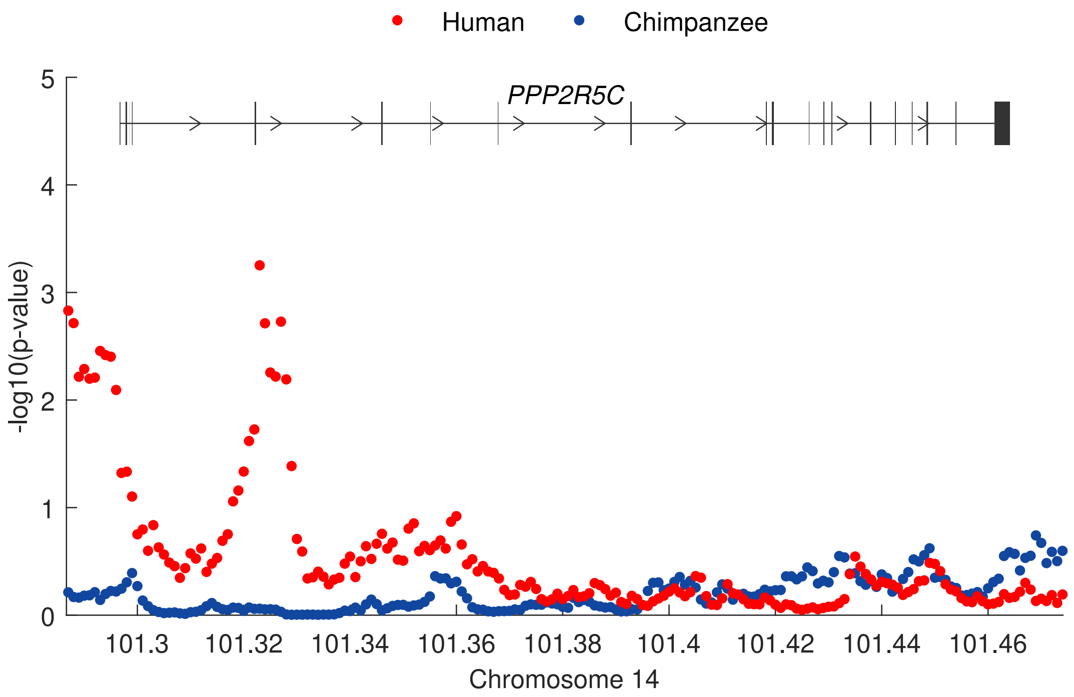

Fig. S36. -log10(p-value) of normalized λ around the positively selected gene *PPP2R5C*.

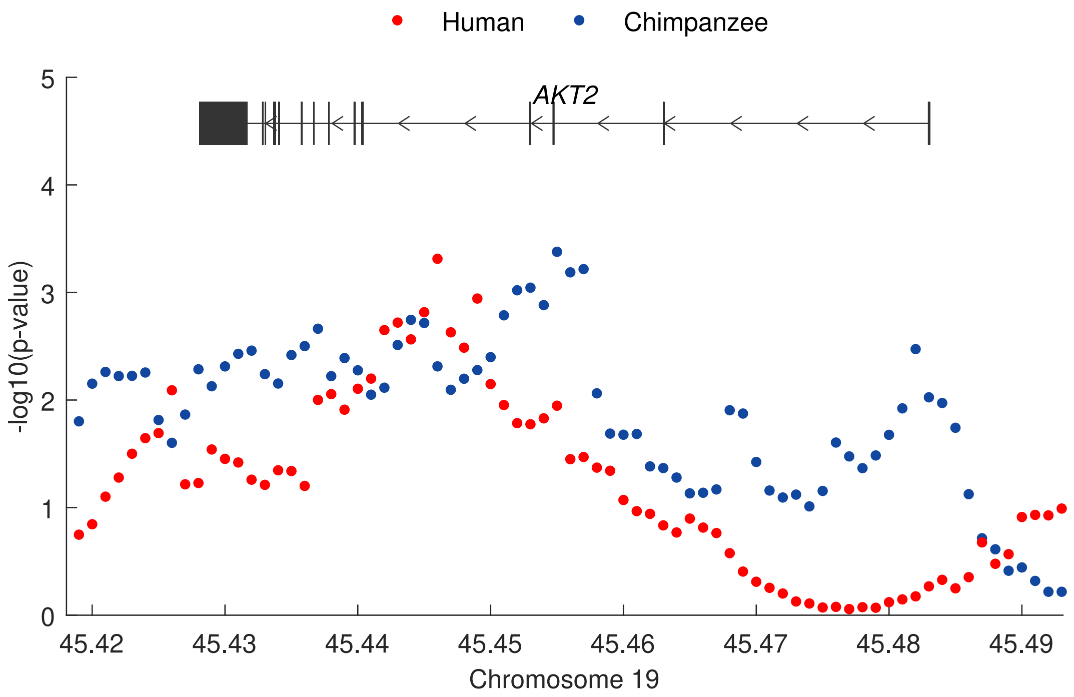

Fig. S37. -log10(p-value) of normalized λ around the positively selected gene *AKT2*.

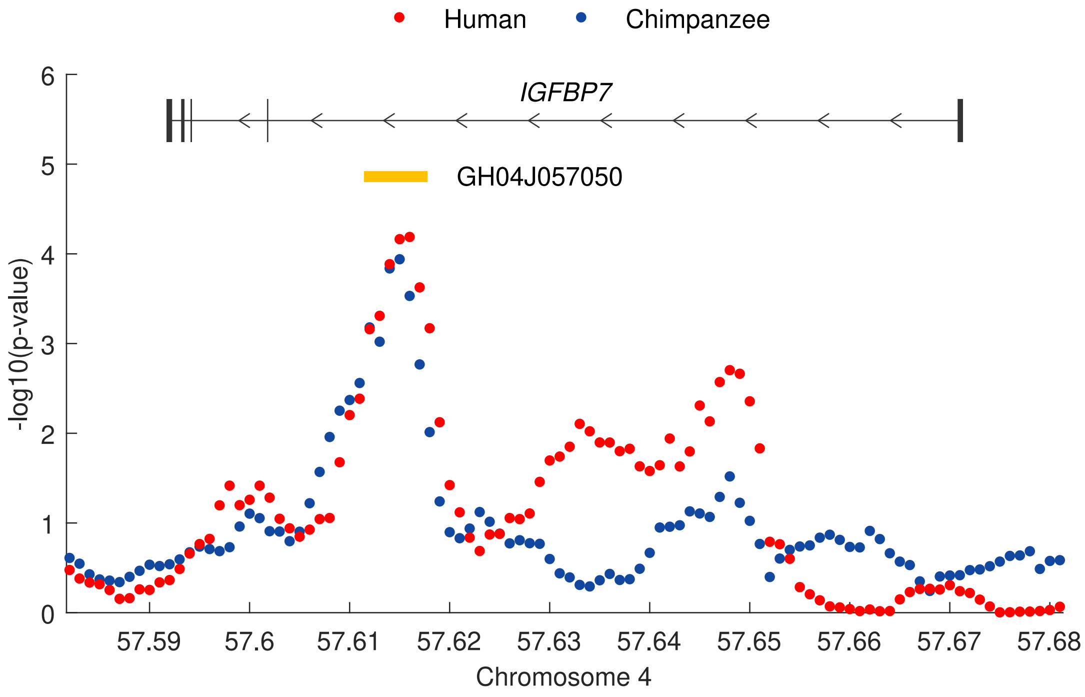

Fig. S38. -log10(p-value) of normalized λ around the *IGFBP7* gene under Balancing selection.

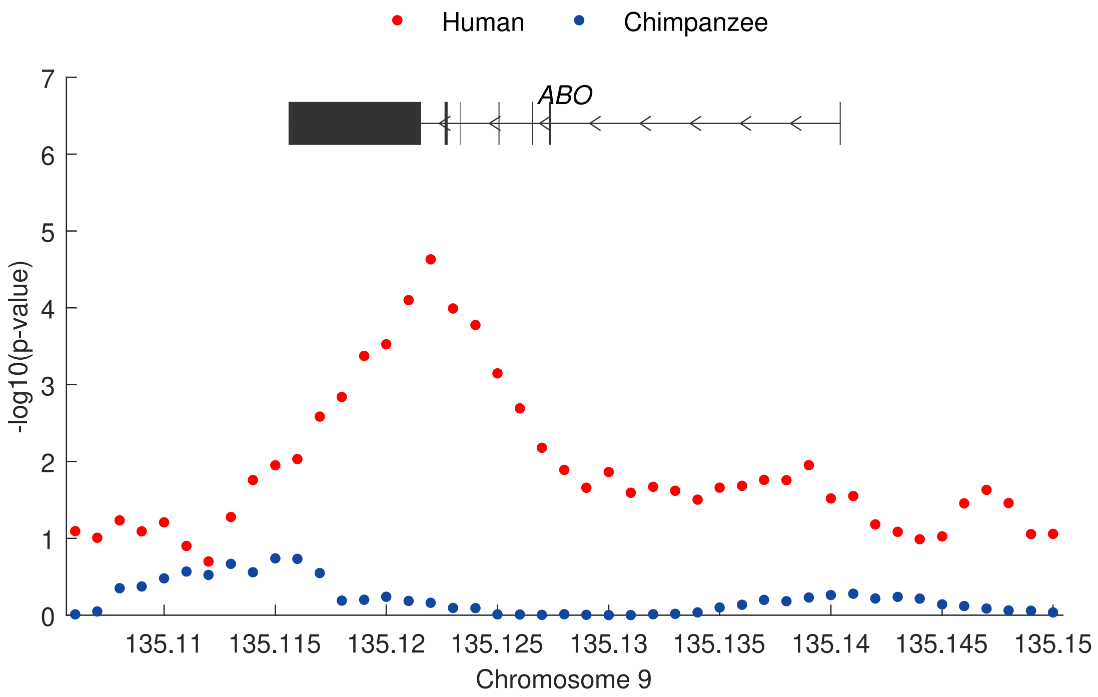

Fig. S39. -log10(p-value) of normalized λ around the *ABO* gene under Balancing selection.
