# Supplementary material for: CEGA: a method for inferring natural selection by comparative population genomic analysis across species
Source: Genome Biol. 2023 Oct 3;24:219. doi: 10.1186/s13059-023-03068-8 (PMC10548728; doi:10.1186/s13059-023-03068-8)
Supplement: Supplementary file 3 — Additional file 3. Peer review history [file 13059_2023_3068_MOESM3_ESM.docx]

**Review history**

**First round of review**

**Reviewer 1**

The manuscript by Zhao et al. presents a new method to infer positive selection as well as balancing selection using patterns of polymorphism and divergence between a pair of species. A key advance over existing approaches is that the new method considers polymorphism from both species simultaneously. The authors show how their new method has improved performance for detecting weaker positive selection as well as recent balancing selection. Overall, I think this method is an important contribution as more and more studies will generate polymorphism data from multiple species. However, the methods to analyze these data and make reliable inferences about selection are still in their infancy. Further, though the HKA test was one of the first major tests for selection and has been shown to have reasonable power, it hasn't been as widely utilized in modern comparative population genetics as much as it should be, in my view. Thus, for these reasons, I view this manuscript favorably. I believe it should be of interest to a wide range of biologists studying genomes and genetic variation.

However, that said, there are a number of technical points that I have major concerns about. I will outline them in more detail below:

1) Background, second paragraph: It should be noted that the HKA and MK tests do not have to use this chi-square approximation. For example, Wright and Charlesworth (2004) consider an explicit likelihood framework for their version of the HKA test. Also, the MK test could use some other sort of contingency table test. Please revise here for accuracy.

2) Background: It would be helpful when discussing the different methods to distinguish between parameter inference genome-wide vs. identification of specific regions of the genome undergoing selection. The field has focused on both questions. The authors should mention this. I also think it presents a nice opportunity to highlight that their present method is geared toward addressing both questions.

3) Background: There are other newer methods that use an MK-like approach to infer parameters pertaining to positive selection. These include:

doi: 10.1101/gr.6691007
doi: 10.1038/s41559-019-0890-6
doi: 10.1093/g3journal/jkac206.
doi: 10.1101/gr.256636.119
doi: 10.1534/g3.120.401052
It would be good to cite these.

4) There has been additional work done on the HKA test by Hideki Innan. I would suggest citing: doi: 10.1534/genetics.106.056242.

5) I wonder if the authors should reconsider the naming of CEGA-InSel, given that it sounds similar to the word "Incel"? I know this is a coincidence, but I don't think the authors wish for their program to even potentially be loosely connected.

6) The authors focus on inferring the lambda parameters as the key parameters in their selection model. Can lambda be connected to the selection or dominance coefficients in the traditional selection models used in population genetics? Doing so would make the parameter estimates so much more meaningful.

7) "Modeling lineage-specific positive selection and balancing selection: "….on a gene locus in species j, j=1,2," reads a bit awkwardly. Please rephrase.

8) The authors use the genome-wide distribution of lambda values to obtain a p-value. Wouldn't a likelihood ratio test of lambda=1 vs. lambda!=1 be a more powerful test here? It would also allow for an explicit model comparison, rather than just picking outlier regions just based on their rank in the empirical distribution.

9) Figure 2: There is a focus on power of the new test for positive selection. How good are the estimates of lambda? As one of the main goals of this paper is parameter inference, rather than just testing for the presence of selection, there needs to be some evaluation of the quality of the estimates of the parameter.

10) The simulations assume that the correct form of the demographic model is being fit to the data. What if in truth the demographic model involves a population bottleneck or expansion in one of the modern populations since the split? How would such a population size change that was unaccounted for affect inference?

11) There was no mention of recombination in the model or the simulations. How is that handled in the method? Is the method sensitive to the presence/absence of recombination? This is a critical area that seems to have been overlooked in the manuscript.

12) What significance level/cutoffs were used to identify targets of selection in the human genome? Do the human candidates for selection have unusually high/low recombination rate? How do you interpret that pattern, if there is one?

13) Methods: How you optimize the likelihood function? How do you compute the probabilities of the different numbers of segregating sites in equations 4-7? Is that done analytically? More detail should be provided.

14) In the SLiM simulations of balancing selection, is the one variant under selection placed in the segment? Or, do mutations occur with some probability and then you sample a run when a mutation occurs? Do any mutations become lost? What do you do with those replicates?

15) Tables 1 & 2, enrichment analyses: More details should be given about how the enrichment analyses were performed. How many genes were in the background set used for comparison? The P-values in Table 1 seem very small. More clarification is need as to how they were generated.

**Reviewer 2**

This manuscript proposes a new population genetic/statistical method (CEGA-InSel) to infer natural selection, both directional and balancing, from multilocus divergence and polymorphism data. Its strength over previous similar methods (HKA, MKPRF or HDMKPRF) is that (1) it can be applied to non-coding regions and (2) it outperforms existing methods for both types of selection. As proof of its performance, the methods when applied to whole genome data from humans and chimpanzees find a host of genes and pathways through human evolution (342 novel genes with selection signals in regulatory and/or non-coding regions), paving the way for further research.
The ms is a well-written, well-argued. It is a piece of sound research. The reason why the CEGA-InSel method outperforms other approaches is clearly established. It explicitly models shared genetic polymorphism among closely related species, which empower the detection of selection.
I have not verified the demonstrations or replicated the results, but previous related results and publications by the authors, mainly Dr. Chen, allow us to trust that they are serious and reliable.
The discussion is brief, unbalanced when compared with other sections. It could go more into assessing the limitations of the method, for example, when there is demographic fluctuations or population structure. In addition, it can be discussed how this software should be applied to other species with very different genome or demographic properties.
The software that implements CEGA-InSel is briefly explained in a pdf manual at GitHub. Information about run time required for different amounts of genome data would be useful, as well as for which data set it would be appropriate to use cluster computing.

Specific points:
Page 15, lines 52:56. I miss a reference or reasoning supporting the inferred Ne for Chimps and Humans
Page 15, line 58:59. Reference should be substituted by the reference number
Reference section is repeated twice in the ms

**Authors’ response to reviewers**

Reviewer #1: The manuscript by Zhao et al. presents a new method to infer positive selection as well as balancing selection using patterns of polymorphism and divergence between a pair of species. A key advance over existing approaches is that the new method considers polymorphism from both species simultaneously. The authors show how their new method has improved performance for detecting weaker positive selection as well as recent balancing selection. Overall, I think this method is an important contribution as more and more studies will generate polymorphism data from multiple species. However, the methods to analyze these data and make reliable inferences about selection are still in their infancy. Further, though the HKA test was one of the first major tests for selection and has been shown to have reasonable power, it hasn't been as widely utilized in modern comparative population genetics as much as it should be, in my view. Thus, for these reasons, I view this manuscript favorably. I believe it should be of interest to a wide range of biologists studying genomes and genetic variation.

Response: We thank the reviewer for being positive of our work.

However, that said, there are a number of technical points that I have major concerns about. I will outline them in more detail below:
1) Background, second paragraph: It should be noted that the HKA and MK tests do not have to use this chi-square approximation. For example, Wright and Charlesworth (2004) consider an explicit likelihood framework for their version of the HKA test. Also, the MK test could use some other sort of contingency table test. Please revise here for accuracy.

Response: We thank the reviewer for the suggestions. We have changed the sentence into “Both the HKA and MK tests use the chi-squared test or Fisher exact test” in Line 53. The method by Wright and Charlesworth (2004) was cited in line 65-68. We have stated that “Analogously, MLHKA [5] is a model-based method for HKA. It explicitly models the numbers of divergent sites between two species and polymorphic sites in a population of a single species and applies a maximum likelihood ratio test to detect directional selection.”

2) Background: It would be helpful when discussing the different methods to distinguish between parameter inference genome-wide vs. identification of specific regions of the genome undergoing selection. The field has focused on both questions. The authors should mention this. I also think it presents a nice opportunity to highlight that their present method is geared toward addressing both questions.

Response: We thank the reviewer for the suggestion. We have added the content in the Introduction section. Please refer to
Line 72-78: “Parametric methods explicitly model the effect of selection on the genetic polymorphism pattern, and thus, in addition to being significant tests, these methods are useful for inferring parameters of the selective sweep processes. Numerous studies have concentrated on applying these methods to analyze genomic data to characterize the essential parameters of natural selection, such as, the distribution of fitness effects and the rate of adaptation [10-16].”
Line 109-111 in the revised manuscript: “furthermore, the method can accurately infer evolutionary parameters, including the selection intensity, providing more insights into the selection process.”

3) Background: There are other newer methods that use an MK-like approach to infer parameters pertaining to positive selection. These include:
doi: 10.1101/gr.6691007
doi: 10.1038/s41559-019-0890-6
doi: 10.1093/g3journal/jkac206.
doi: 10.1101/gr.256636.119
doi: 10.1534/g3.120.401052
It would be good to cite these.

Response：We thank the reviewer for suggesting these related papers, we have cited them in the revised manuscript (Line 78).

4) There has been additional work done on the HKA test by Hideki Innan. I would suggest citing: doi: 10.1534/genetics.106.056242.

Response: We have cited the paper in the revised manuscript. “Multiple methods were proposed to tackle the problem of low power. Some methods modify the HKA test and include more summary statistics [4]; and other more complicated parametric methods model the data pattern by incorporating the evolutionary processes.” Please refer to Lines 56-59 in the revised manuscript.

5) I wonder if the authors should reconsider the naming of CEGA-InSel, given that it sounds similar to the word "Incel"? I know this is a coincidence, but I don't think the authors wish for their program to even potentially be loosely connected.

Response: We thank the reviewer for the suggestion. We have now changed the name of the software to “CEGA”.

6) The authors focus on inferring the lambda parameters as the key parameters in their selection model. Can lambda be connected to the selection or dominance coefficients in the traditional selection models used in population genetics? Doing so would make the parameter estimates so much more meaningful.

Response: We thank the reviewer for the suggestion. We agree with the reviewer that connecting selection intensity to lambda, and inferring the parameter are very meaningful. In the revised manuscript, we add a new section on it. We spend much effort constructing the connection between the value of λ and selection coefficients.

The existing theoretical studies provide a foundation for the method (Stephan, Wiehe, and Lenz, Theoretical Population Biology (1992); Wiehe and Stephan, Molecular Biology and Evolution (1993), and others). λ, the ratio of reduced effective population size attributed to selection and the effective population size under neutrality, can be approximated with the ratio of genetic heterogeneity: λ≈H/H_neu=N/N_neu. Following former studies, we can easily derive λ as a function of selection intensity, and recombination rate of the local region.

The details can be found in “Parametric inference of selection intensity of recurrent selective sweeps” section in Methods.

We carried out a new set of simulations to evaluate the performance of parameter inference on the selection intensity of recurrent sweeps. Surprisingly, we found that the inference of selection intensity is unbiased and quite accurate (see Fig 2(E-F) in the main text). The results indicate that the theoretical model characterizes well the hitchhiking effects of recurrent selective sweeps on nearby regions; the reduction of genetic heterogeneity is indeed informative for inferring selection intensity.

Thus, following the reviewer’s suggestion, in the revised manuscript we boost the performance of CEGA by providing an additional functionality for inference of selection intensity. CEGA is useful not only for serving as a powerful test for selection, but also providing parameter inference. On the other hand, the theoretical part developed so far is still in some respects simplified, we will explore more on this question and improve the performance of parameter inference in the future work.

7) "Modeling lineage-specific positive selection and balancing selection: "….on a gene locus in species j, j=1,2," reads a bit awkwardly. Please rephrase.

Response: The sentence has been rephrased as below:
“When recurrent positive selection acts on a gene locus in species j, the polymorphism level within species j is reduced due to the hitchhiking effect…”. Please refer to Lines 202-206 in the revised manuscript.

8) The authors use the genome-wide distribution of lambda values to obtain a p-value. Wouldn't a likelihood ratio test of lambda=1 vs. lambda!=1 be a more powerful test here? It would also allow for an explicit model comparison, rather than just picking outlier regions just based on their rank in the empirical distribution.

Response: Thank you for the advice. We have conducted the likelihood ratio test. We set the null hypothesis: lambda1=1, lambda2=1, and local mutation rate is free variable. The alternative hypothesis 1 is: lambda1 is free, while lambda2=1, local mutation rate is free. The alternative hypothesis 2 is: lambda2 is free, while lambda1=1, local mutation rate is free. In the analysis of human and Chimpanzee genome data, we found that the LRT score does not follow a chi-squared distribution with one degree of freedom . However, it is under the distribution after adjustment with an inflation factor.

Figure R1 (in the pdf version of the cover letter) shows the probability density function of (red line) and the distribution of adjusted LRT scores from the analysis results of Human and Chimpanzee data.


We then compared the power of CEGA based on the empirical distribution of λ (after box-cox transformation, lambda1 and lambda2 are under normal distribution, the method is denoted as CEGA-λ) and the likelihood ratio test (CEGA-LRT). We applied CEGA to simulation data described in the main text. As shown in Figure R2, CEGA-LRT seems slightly more conservative than CEGA-λ. The mean power of CEGA-λ, CEGA-LRT, and MLHKA for data simulated with the divergence time of 200,000 generations are 0.7408, 0.6160, and 0.5010, respectively. For data simulated with the divergence time of 40,000 generations, the mean power of CEGA - λ, CEGA - LRT, and MLHKA are 0.7692, 0.5520, and 0.4483, respectively.

For balancing selection, we compared the two ways using simulation data sets described in the main text. CEGA-λ still performs better than CEGA - LRT (Figure R3).

We further checked the -logp values of the LRT scores around genes detected by CEGA-λ shown in the main text. The pattern of -logp values around all genes are similar between CEGA - LRT and CEGA-λ (Figures R4 and R5).

In summary, CEGA-λ outperforms CEGA-LRT in detecting positive selection and balancing selection. Thus, in the section of “Real data analysis” in the main text, we still present the results from CEGA-λ as in the former manuscript. We provide a new option -LRT in the software command line for users to conduct a likelihood ratio test in addition to the previous strategy to calculate the p-value from the adjusted distribution of λ. The above contents are listed in the Supplementary information (Section 7) and main text (Line 232-239).

9) Figure 2: There is a focus on power of the new test for positive selection. How good are the estimates of lambda? As one of the main goals of this paper is parameter inference, rather than just testing for the presence of selection, there needs to be some evaluation of the quality of the estimates of the parameter.

Response: In the forward simulations, we did not directly set values of λ. Instead, we set the values of selection intensity (see details of simulations in Methods section). The data generated from these simulations cannot be used to evaluate the accuracy of inference of λ. Thus, to directly test how good the estimates of λ, we carried out a new set of simulations by directly setting λ values using the coalescent simulation software MSMS.

For the population under selection, we set its effective population size to be λNe. We used a wide range of λ=0.1, 0.3, 0.5, 0.7, 0.9, 1.5, 2, and 3. The values of λ<1 correspond to positive selection, and the values of λ>1 correspond to balancing selection. We then applied CEGA to the 200 simulated samples. As shown in Figure R6, the estimations of λ are overall unbiased. The details of the MSMS simulations and results are presented in Section 4 of Supplementary information.

Following the reviewer’s suggestion, we added a section for parameter inference of selection intensity. Under the population genetic model, λ has biologically meaningful connection with the selection intensity s. Please see Response to Question 6. And as we show in Fig 2(E-F), the inference of selection intensity is also unbiased and relatively accurate.


10) The simulations assume that the correct form of the demographic model is being fit to the data. What if in truth the demographic model involves a population bottleneck or expansion in one of the modern populations since the split? How would such a population size change that was unaccounted for affect inference?

Response: We thank the reviewer for the suggestion. We address this question using simulation. We considered six demographic models:
Constant effective population size (Model A): Ne=10,000 individuals.
Ancient severe bottleneck (A-S-BN): bottleneck duration: 30600-30000 generations ago; bottleneck population size: 0.1Ne.
Ancient mild bottleneck (A-M-BN): bottleneck duration: 30200-30000 generations ago; bottleneck population size: 0.1Ne.
Recent severe bottleneck (R-S-BN): bottleneck duration: 4000-3400 generations ago; bottleneck population size: 0.1Ne.
Recent mild bottleneck (R-M-BN): bottleneck duration: 4000-3800 generations ago; bottleneck population size: 0.1Ne.
Exponential growth (EG): duration: 923-0 generations ago; growth rate: 0.5% per generation; size change: from Ne=10000 to Ne=1000,000.

As expected, ancient bottlenecks have only a slightly effect on the observed polymorphic sites compared with the constant demographic model. For R-S-BN, R-M-BN, A-S-BN, and A-M-BN, the polymorphic sites are reduced to 79.86%, 91.25%, 96.07%, 98.58% of that of Model A. For the exponential growth model, the polymorphic sites increased to 108.62% of the Model A (Figure R7). All demographic models have a minor effect on the number of divergence sites (Figure R7).

We then applied CEGA to the simulated data from the five non-constant population models. In the inference, CEGA still fitted a constant population size. For R-S-BN, R-M-BN, A-S-BN, A-M-BN, and exponential growth model, the estimated constant population sizes are 78.46%, 90.43%, 95.19%, 98.34%, and 109.25% of 10,000 (Figures R8).

However, when we predict the number of polymorphism sites with CEGA by assuming the population size being the inferred population size, we observed that the polymorphism site numbers are very close to the numbers from simulated data (Figure R9). This result demonstrated that, the constant effective population size model implemented in CEGA can approximate the non-constant size models quite well, in terms of the polymorphism pattern, indicating the robustness of the method to simplified demographic history models. Using a constant population approximation is reasonable especially for CEGA, since CEGA aims to detect recurrent sweeps or balancing selection, which last for a long time, and are less affected by population size fluctuation during a short time interval.

On the other hand, in cases that the population experienced recent demographic changes, and the researchers prefer to fit a non-equilibrium model in CEGA. A parametric model with changing population sizes can be fitted using the joint allele frequency spectrum methods (e.g., Excoffier et al, PLoS Genetics 2013), and then the summary statistics under the inferred demographic model can be obtained using theoretical results from former studies (Chen and Chen, Genetics 2013; Chen, Genomics Proteomics Bioinformatics 2019).

Please see Supplemental section 6 and Line 559-575 for more details.

11) There was no mention of recombination in the model or the simulations. How is that handled in the method? Is the method sensitive to the presence/absence of recombination? This is a critical area that seems to have been overlooked in the manuscript.

Response: When simulating the data, we set a recombination rate of 10-8 per base pair per generation. In the model of CEGA, we assume no recombination within a segment, while free recombination among segments. This is the assumption adopted by HKA and MLHKA, and many other methods. By the assumption, it is easy to derive theoretical results for the four summary statistics. This assumption was proved to be reasonable in building the methods, since recombination rate only affects the variance of the joint allele frequency spectrum and related summary statistics. It does not affect the mean values of the summary statistics (see Wakeley and Hey, Genetics (1997), Chen, Theoretical Population Biology (2012), and others).

12) What significance level/cutoffs were used to identify targets of selection in the human genome? Do the human candidates for selection have unusually high/low recombination rate? How do you interpret that pattern, if there is one?

Response: The details of significance level/cutoffs are provided in the Method section (Genomic data analysis). Windows with a normalized value <-3.2905 were identified as under positive selection, and windows with a normalized value >3.2905 were identified as under balancing selection. The cutoff is equivalent to a total false positive rate of positive selection and balancing selection of 0.001.

We calculated the mean recombination rate of regions identified as being under positive selection and balancing selection in humans. We found that these regions indeed show a lower recombination rate than neutral regions (0.60×10-8 of genes under positive selection, 0.97×10-8 of genes under balancing selection, and 1.35×10-8 of neutral regions). The phenomenon can be interpreted as below:
The fine-scale recombination map of human genome was developed by Donnelly, McVean, Myers and colleagues (Science 2005) using population genetic methods. Positive selection or balancing selection acting on a region can result in higher linkage disequilibrium level, and lead to an underestimate of recombination rates in the region by the recombination-rate inference methods.
Regions with low recombination rates when being under positive selection or balancing selection are easier to be detected by population genetic methods, including CEGA.
Neither of the above two cases cause false-positive results in data analysis.

13) Methods: How you optimize the likelihood function? How do you compute the probabilities of the different numbers of segregating sites in equations 4-7? Is that done analytically? More detail should be provided.

Response: The details of how we optimize the likelihood function was shown in the Method section (Two-step maximum likelihood estimation of parameters). Briefly, we construct the likelihood function as the probabilities of observed numbers of segregating sites with theoretically expected values provided in equations 4-7. We optimize the likelihood function using a two-step scheme: first, inferring the global parameters under the genome-wide data, and set λ_1^l=1 and λ_2^l=1; Second, fix the global parameters to the values inferred in the first step, and then infer the local parameters for each local region. Please refer to Lines 615-630 in the revised manuscript.

Equation 4-7 provides the expectations of the four summary statistics from the joint allele frequency spectrum (JAFS), and JAFS is calculated analytically following former study (Chen, Theoretical Population Biology 2012). The likelihood function (probability of the data) is constructed by assuming Poisson distributions for the four summary statistics. The likelihood function and the details can be found in “Two-step maximum likelihood estimation of parameters” of the Method section.

14) In the SLiM simulations of balancing selection, is the one variant under selection placed in the segment? Or, do mutations occur with some probability and then you sample a run when a mutation occurs? Do any mutations become lost? What do you do with those replicates?

Response: In the SLiM simulations of balancing selection, we placed one variant under selection in the middle of the segment at a pre-set time using the SLiM build-in function addNewDrawnMutation. Most of the mutations become lost. We discarded those samples since no expected balancing selection happens. For efficiency, once detecting the loss of the mutation, we start a newly seeded run from the recorded running state of the SLiM at time we introduce the mutation. We provided the details of the SLiM simulation code of balancing selection and positive selection in the Supplementary information.

15) Tables 1 & 2, enrichment analyses: More details should be given about how the enrichment analyses were performed. How many genes were in the background set used for comparison? The P-values in Table 1 seem very small. More clarification is need as to how they were generated.

Response: In the enrichment analyses of genes in humans (Tables 1 & 2), all human genes were used as background gene sets for comparison. To do this, we implemented the gene-list enrichment module of KOBAS-i (http://kobas.cbi.pku.edu.cn/genelist/), which is based on the ORA (overrepresentation analysis) method. We choose Homo sapiens (human) as the background species. The statistics of Homo sapiens provided in KOBAS-i include: 39244 total genes, 7868 genes with KEGG annotation, 10519 genes with Reactome annotation, 1149 genes with BioCyc annotation, 2262 genes with Panther annotation, 19010 genes with GO annotation (These statistics can be found in http://kobas.cbi.pku.edu.cn/download/). Please refer to Lines 346-349 in the revised manuscript.

Reviewer #2: ===
This manuscript proposes a new population genetic/statistical method (CEGA-InSel) to infer natural selection, both directional and balancing, from multilocus divergence and polymorphism data. Its strength over previous similar methods (HKA, MKPRF or HDMKPRF) is that (1) it can be applied to non-coding regions and (2) it outperforms existing methods for both types of selection. As proof of its performance, the methods when applied to whole genome data from humans and chimpanzees find a host of genes and pathways through human evolution (342 novel genes with selection signals in regulatory and/or non-coding regions), paving the way for further research.
The ms is a well-written, well-argued. It is a piece of sound research. The reason why the CEGA-InSel method outperforms other approaches is clearly established. It explicitly models shared genetic polymorphism among closely related species, which empower the detection of selection.
I have not verified the demonstrations or replicated the results, but previous related results and publications by the authors, mainly Dr. Chen, allow us to trust that they are serious and reliable.

Response：We are grateful to the reviewer for being positive of our work.

The discussion is brief, unbalanced when compared with other sections. It could go more into assessing the limitations of the method, for example, when there is demographic fluctuations or population structure. In addition, it can be discussed how this software should be applied to other species with very different genome or demographic properties.

Response: We thank the reviewer for the suggestion. We added more sentences in the Discussion section, discussing about the limitations of the methods, including the effects of demographic fluctuations, the computational efficiency and the applicability to species with different genomic properties.

In Lines 559-575, we added: “CEGA approximates the demographic history of different species with constant effective population sizes. The approximation is reasonable since CEGA focuses on recurrent selective sweeps or balancing selection over a relatively long-term period. Simulations of five non-equilibrium demographic scenarios were carried out to evaluate the simplified model of CEGA, including ancient severe bottleneck, ancient mild bottleneck, recent severe bottleneck, recent mild bottleneck and recent exponential growth (see Supplementary Section 6 for detailed parameter settings). As shown in Figs. S8-S10, the polymorphic and divergent site patterns are approximately equivalent to those from constant-size model with the effective population size inferred using CEGA. This suggests the robustness of the method to simplified demographic history models. While approximating with constant effective population sizes works well for non-equilibrium demographic histories, CEGA is capable of accommodating more complex demographic models if it is necessary. A parametric model with changing population sizes can be fitted using the joint allele frequency spectrum methods ([20, 23, 87] and others), and then the expected values of the four summary statistics under the inferred demographic model can be obtained using former theoretical results and incorporated into the likelihood function of CEGA [21, 88].”

In lines 577-580, we added: “Another practical issue arises from the fact that the method was developed based on the model assumption of random mating species and recombinant genomes. For certain species with distinct breeding histories, such as a selfing species, or species exhibiting very low or zero recombination rates, the method is not applicable.”

The software that implements CEGA-InSel is briefly explained in a pdf manual at GitHub. Information about run time required for different amounts of genome data would be useful, as well as for which data set it would be appropriate to use cluster computing.

Response: We added a paragraph in Discussion section to discuss about the computational efficiency: “CEGA is computationally efficient, making it feasible to apply to genome-wide data analysis. Additionally, CEGA provides multi-threaded mode, which allows for parallel processing and further improves the efficiency. As an example, it takes 8 hours for CEGA to analyze the whole genome of nine Homo sapiens and nine Pan troglodytes with 2,416,717 sliding windows of 10 kb (using 40 threads, Intel(R) Xeon(R) Gold 6230 CPU @ 2.10GH).”

Specific points:
Page 15, lines 52:56. I miss a reference or reasoning supporting the inferred Ne for Chimps and Humans

Response: In line 340, the reference supporting the inferred Ne for Chimps and Humans is listed in the end of the sentence. For clarity, we revised the sentence as “The ratio of Nc/Nh=1.3787 is close to 1.3978 estimated by Zhao et al. (2019) [7]”.

Page 15, line 58:59. Reference should be substituted by the reference number
Reference section is repeated twice in the ms

Response: Thank you for pointing it out. We have deleted the repeated references.

**Second round of review**

**Reviewer 1**

The authors have done a fantastic job revising the manuscript to address all of my previous comments. The revision is thorough and comprehensive. I do not have any additional comments.
